# Endovascular EEG device prospective multicenter single-arm clinical trial to confirm efficacy and safety performance on patients with Intractable Epilepsy: The EPSILON IE trial protocol

**DOI:** 10.1371/journal.pone.0332387

**Published:** 2025-10-16

**Authors:** Yuji Matsumaru, Ayataka Fujimoto, Yosuke Masuda, Hisayuki Hosoo, Kota Araki, Aiki Marushima, Kensuke Kawai, Hiroki Ishida, Koichi Hashimoto, Ryota Ishii, Bryan J. Mathis, Eiichi Ishikawa

**Affiliations:** 1 Department of Neurosurgery, Institute of Medicine, University of Tsukuba, Tsukuba, Japan; 2 Epsilon Medical Inc., Tokyo, Japan; 3 Epilepsy Center, Dokkyo Medical University School of Medicine, Tochigi, Japan; 4 School of Rehabilitation Sciences, Seirei Christopher University, Shizuoka, Japan; 5 Seirei Hamamatsu General Hospital, Hamamatsu, Shizuoka, Japan; 6 Department of Neurosurgery, Jichi Medical University, Shimotsuke, Tochigi, Japan; 7 Tsukuba Clinical Research and Development Organization (T-CReDo), University of Tsukuba, Tsukuba, Japan; 8 Department of Biostatistics, Institute of Medicine, University of Tsukuba, Tsukuba, Japan; 9 Clinical Research Manuscript Elevation Service (CReME), University of Tsukuba, Tsukuba, Japan; Jikei University School of Medicine, JAPAN

## Abstract

**Introduction:**

Epilepsy affects approximately 70 million people globally, with around 20−30% of these individuals experiencing drug-resistant epilepsy in which seizures remain uncontrolled despite prolonged treatment with anti-seizure medications (ASMs). Such refractory epilepsy significantly impairs quality of life, often necessitating surgical resection of the epileptic focus when ASMs fail. Accurate localization of the epileptic focus is crucial for successful surgery and typically requires invasive intracranial monitoring through subdural electrodes (SDE) or stereotactic electroencephalography (SEEG). Despite their effectiveness, the invasiveness of these methods poses significant risks. In response to these challenges, the EP-01 device has been developed to measure intracranial electroencephalogram (EEG) via the cerebral veins, offering a less invasive alternative. The Endovascular EEG Device Prospective Multicenter Single-arm clinical trial to confirm efficacy and safety performance on patients with Intractable Epilepsy (EPSILON IE) trial aims to evaluate the efficacy and safety of EP-01 in diagnosing the lateralization of epileptic foci in patients with focal epilepsy. The hypothesis is that EP-01, when equipped with multiple endovascular EEG electrodes, can accurately diagnose lateralization, reducing the need for more invasive procedures like SDE and SEEG.

**Methods and analysis:**

This multicenter, prospective, single-arm validation clinical trial is set to take place from March 2024 to August 2025, with follow-up extending to August 2026. The study will enroll 37 patients with refractory focal epilepsy across several Japanese medical institutions. Eligibility criteria include age 15–70 years and a vascular anatomy that allows the EP-01 to be guided into cerebral veins close to the epileptic focus. The EP-01 device will be inserted via the jugular veins, with electrodes positioned in target cerebral veins to record intracranial EEG data. The primary endpoint is the percentage agreement in lateralization diagnosis between EP-01 and conventional intracranial electrodes. Secondary endpoints include the diagnostic performance of EP-01, safety assessments, and seizure outcomes one year after resection surgery. Participants will undergo a screening period of 30 days, followed by the clinical trial period of up to two weeks, during which EP-01 will be inserted and monitored. A post-observation period of one week will follow device removal to assess potential adverse events. Data collection will involve EEG recordings, imaging studies, and safety evaluations, with results analyzed to determine the efficacy and safety of the device compared to traditional methods. This trial aims to provide critical data on the potential for EP-01 to serve as a less-invasive, effective alternative for diagnosing epileptic focus lateralization, potentially reducing the need for traditional invasive monitoring methods.

## 1. Introduction

### 1.1 Background and Rationale

Approximately 20–30% of patients with epilepsy worldwide are non-responsive to drug treatments and experience refractory seizures [1]. Refractory epilepsy is defined as a condition in which seizures remain uncontrolled for at least one year and interfere with daily life, even after at least two years of treatment with a single, primary anti-seizure medication (ASM) or two or more ASMs in combination with sufficient doses.

Approximately 70 million people around the world suffer from epilepsy [2], of whom approximately 20–30% have intractable epilepsy that remains unresponsive to pharmacotherapy [1]. Since ASMs fail to control seizures in these patients, surgical resection of the epileptic focus is considered the method of last resort. Currently, epileptic foci are first non-invasively identified using magnetic resonance imaging (MRI), computed tomography (CT), single-photon emission CT (SPECT), positron emission tomography (PET), and scalp electroencephalography (EEG) [3]. However, in numerous cases, determination of the resection area ultimately rests in invasive procedures such as placement of subdural electrodes (SDE) or stereotactic EEG (SEEG) before proceeding to focal resection [3,4]. Accurate estimation of the epileptic focus by invasive monitoring followed by successful resection surgery is key to increasing the chances of better seizure control in patients with intractable epilepsy.

Intracranial electrodes have therefore taken a lead role in determining the resection area. These electrodes are used for estimating the potential epilepsy focus and functional mapping [4,5]. The two types of intracranial electrode placement procedures are SDE and SEEG [3,4]. SDE covers the brain surface “as a plane” and implants electrodes subdurally by craniotomy. SDE is more invasive than SEEG, but allows the performance of functional mapping. By virtue of its reduced invasiveness, SEEG becomes more applicable in re-operative cases where SDE placement is difficult or where electrode placement in both hemispheres is desirable to determine the lateralization of the epileptic focus [3,4]. These intracranial electrodes are sometimes used alone in SDE or SEEG, and sometimes are used in combination, and are considered the gold standard for identifying the epileptic focus to decide the extent of resection [1]. Even though these procedures are invasive, false lateralization from scalp EEG that differs from intracranial electrodes can result in failed surgical isolation of the focus [6], so appropriate lateralization diagnosis by detecting signals directly from close to the epileptic focus in the brain parenchyma is very important.

To avoid signal attenuation by the skull and scalp, and to reduce the invasiveness of obtaining intracranial EEG compared to SDE and SEEG, we have considered the cerebral vasculature as a site from which to record intracranial EEG. We are developing EP-01 to measure intracranial EEG from within the cerebral veins by approaching from inside the blood vessels. The two main objectives of SDE electrodes are “intracranial EEG measurement” and “functional mapping”, but EP-01 is currently monopolar, making functional mapping impractical. However, we believe that the objective of “intracranial EEG measurement“by lateralization diagnosis of the epileptogenic zone can be achieved with endovascular EEG.

Mesial temporal lobe epilepsy is particularly effective for lateralization diagnosis because once lateralization is determined, amygdalohippocampectomy is performed on the determined side, so more precise identification of the resection area is unnecessary. Among focal resection procedures, amygdalohippocampectomy is known to be highly effective, with over 90% of patients successfully treated in terms of seizure control [7,8]. However, as mentioned above, accuracy in preoperative focal diagnosis is extremely important when performing other resection surgeries, since inaccurate preoperative focus diagnosis or lateralization will render the surgery ineffective. Intracranial EEG recording is therefore regarded as one of the most accurate methods of focal diagnosis since ictal EEG changes can be directly recorded from the site of seizure onset [9].

Since the 1960s, lateralization diagnosis has been performed using SEEG, and lateralization can be diagnosed even with a small number of electrodes. We therefore believe that lateralization diagnosis is also possible with EP-01. In addition, a case report in which a wire-like endovascular EEG electrode was placed in the cerebral artery showed that simultaneous placement of intracranial electrodes and endovascular EEG electrodes in a patient with temporal lobe epilepsy yielded similar epileptic spikes from both [10]. This endovascular EEG measurement was performed with only one electrode, but measurement from a single location is suboptimal for determining lateralization. Insertion of multiple electrodes into both cerebral hemispheres is thus needed to improve endovascular EEG.

### 1.2 Objectives and hypothesis

To assess the efficacy and safety of cerebral endovascular EEG for determining epileptic focus laterality in focal epilepsy, based on the hypothesis that simultaneous placement of multiple endovascular EEG electrodes in both hemispheres enables accurate lateralization.

### 1.3 Trial design

This multicenter, prospective, single-arm, validation clinical trial will be conducted on the study device from March 2024 to August 2025. The follow-up period for seizure evaluation will extend to August 2026. Evaluations of EP-01insertion performance will be determined by a Central Evaluation Committee comprising expert clinicians.

**Fig 1:**
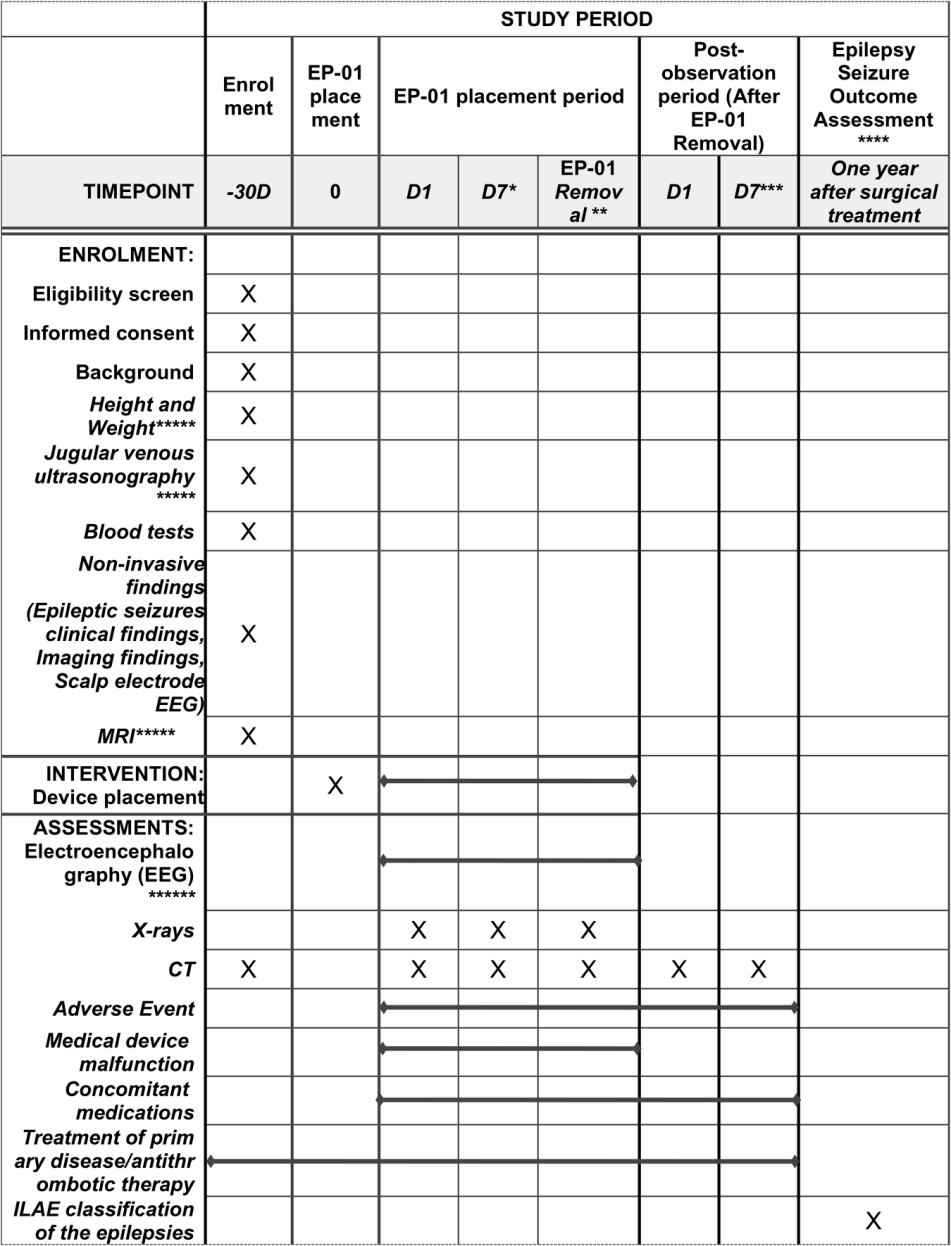
Study schedule of EPSILON IE. Essentially, the screening period is set at 30 days and the investigational testing period after insertion is a maximum of 2 weeks to facilitate testing and provide sufficient time for non-provoked spontaneous seizures to occur. The post-observation period (1 week) is judged as sufficient for monitoring potential adverse events associated with endovascular and intracranial implantation of electrodes. *Not required if epileptic seizures occur within one week of placement of the test device. ** EP-01 will be left in place until the onset of epileptic seizures or for a maximum of 14 days (plus 3 days). ***If the trial is to be stopped during the clinical trial inspection period, the tests specified at the time of removal of the EP-01 will be carried out as much as possible depending on the subject’s condition, the EP-01 will be removed, and the trial will move to the post-observation period. Suppose the trial is to be stopped during the post-observation period. In that case, the tests specified one week after removal of the test device will be carried out as much as possible depending on the subject’s condition, and the trial will then end. **** Only subjects who underwent epilepsy resection were included. ***** If the measurement is conducted within 90 days before obtaining consent, there is no need to remeasure, and data from before consent was obtained will be collected. ****** When only EP-01 or both EP-01 and intracranial electrodes are indwelled, EEG data from EP-01 and intracranial electrodes will be recorded under video surveillance. EEG data during eyes opening and closing (the investigator, etc. will decide the timing of EEG data acquisition during eyes opening and closing) and EEG data during epileptic seizures will be submitted to the Central Evaluation Committee.

Essentially, the screening period is set at 30 days and the investigational testing period after insertion is a maximum of 2 weeks to facilitate testing and provide sufficient time for non-provoked spontaneous seizures to occur. The post-observation period (1 week) is judged as sufficient for monitoring potential adverse events associated with endovascular and intracranial implantation of electrodes. A summary of the processes for enrollment, conduct of the clinical trial, surgical treatment, and follow-up is shown in the Figure (Fig 1).

This protocol has been described in accordance with the SPRIT checklist (Standard Protocol Items: Recommendations for Interventional Trials [11].

## 2. Methods

### 2.1 Participants, interventions, and outcomes

#### 2.1.1 *Study setting.*

All facilities participating in this multi-center trial are located in Japan. Study locations are the University of Tsukuba, Sapporo Medical University, Jichi Medical University, Tokyo Medical and Dental University, Yokohama City University, Seirei Hamamatsu General Hospital, and Osaka University.

#### 2.1.2 *Eligibility criteria.*

Inclusion criteria:

(1) Patients with refractory epilepsy, including temporal lobe epilepsy, frontal lobe epilepsy, parietal lobe epilepsy, occipital lobe epilepsy, and insular gyrus epilepsy;
(2) Patients with intracranial electrode implantation;
(3) Patients with vascular anatomy that allows the EP-01 to be guided into the brain from the right and left jugular veins to vessels close to the presumed epileptic focus, such as the cavernous sinus, transverse sinus, superior sagittal sinus, and inferior petrosal sinus [the vessel into which the EP-01 will be placed will be determined by the physician, but the EP-01 must first be confirmed as able to be guided to a symmetrical position on both sides of the cerebral vein.]; and
(4) Patient age 15–70 years (at the time of obtaining consent)

Exclusion criteria:

Potential enrollees who display any of the following criteria or enrollees who have provided consent and started participation but display any of the following criteria are to be excluded:

(1) Patients with epilepsy who have generalized seizures only;
(2) Patients with obstruction or thrombus/thrombi in the internal jugular vein;
(3) Patients with a predisposition to bleeding and coagulation abnormalities;
(4) Patients with difficulty remaining still during procedures and/or measurements;
(5) Patients with severe allergies to contrast media;
(6) Patients with metal allergies;
(7) Patients who are pregnant, become pregnant, or are of willful childbearing intent, including patients who fail to use the required birth control methods as outlined in the study protocol;
(8) Patients participating in other clinical trials; or
(9) Patients whom the investigator or sub-investigator determines at any time to be inappropriate to participate in this study

#### 2.1.3 *Planned intervention.*

The planned intervention is the insertion of the EP-01 endovascular EEG (EP-01 main unit and EP-01 accessories; Epsilon Medical Inc., Tokyo, Japan) and the use of devices to evaluate the efficacy and safety of the EP-01; namely, the EX-01 extension wire (Epsilon Medical Inc.) and EEG1200 series EEG (Neurofax/Nippon Kohden Corporation, Tokyo, Japan) that will be used to monitor data from the inserted EP-01.

The vessels for EP-01 placement would be determined by the principal investigator or sub-investigator depending on case circumstances from the following target areas: cavernous sinus, transverse sinus, superior sagittal sinus, and inferior petrosal sinus. In this protocol, placement of EP-01 in the cavernous sinus is considered particularly useful for determining lateralization in mesial temporal lobe epilepsy, as it is located immediately medial to the hippocampal head and amygdala [10]. In contrast, placement in the superior sagittal sinus (SSS), either in the anterior or posterior segments, is not primarily intended for determining lateralization. Instead, such placement is expected to be useful for comparison with other electrode sites and for evaluating the characteristics of epileptiform discharges. For these reasons, symmetrical anterior–posterior placement, as well as bilateral symmetry, is recommended.

The sheath to place the EP-01 will be implanted through the left and right jugular veins with the tip of the sheath pointing toward the cranial side. The microcatheters will then be inserted through the sheath to the target cerebral venous vessels. After verifying positioning (i.e., the exact vessel location), the EP-01 will be inserted into the microcatheter and delivered to the target site. After confirming successful delivery of the EP-01 to the target site, the microcatheter will be withdrawn, using an extension wire if necessary. After microcatheter removal, EEG data acquired at the electrode will be fed into the EEG measuring device via the lead wire. After confirming that the EEG is displayed on the measuring device, implantation of the EP-01 will be considered complete. After insertion is completed, a Tuohy Borst valve or silicone tubing will be used for proper fixation. After implanting a maximum of six wires, the wires protruding from the patient’s neck will be secured to complete the procedure.

To prevent thrombosis of the sheath during implantation of the EP-01 body, continuous perfusion of heparinized saline solution should be performed through the side port of the Tuohy Borst valve attached to the sheath as a rule. If the investigator determines that continuous irrigation with heparinized saline is difficult due to the condition of the subject, flush and lock will be performed at least once a day (heparinized saline is recommended for flushing and locking). During the period of EP-01 implantation, the position of the tip electrode should be confirmed by X-ray examination, and if the position of the tip electrode is found to be significantly misaligned, the safety of the subject will be given highest priority. In addition, the presence or absence of adverse events will be confirmed by CT. If adverse events are confirmed, appropriate treatments will be administered based on the judgment of the investigator.

Note that, because of the complex nature of the study, any therapy that, in the clinical judgment of the investigator or other associated care providers, is necessary for the benefit of the subject may be administered.

Prescribed drugs and therapies used from enrollment to the end of the post-observation period will be noted in the case report as concomitant medications and therapies. Concomitant medications and therapies used for intracranial electrode implantation (including additional implantation) and removal will not be recorded. If focal resection surgery is performed before the end of the post-observation period, concomitant medications and concomitant therapies used for these procedures are also excluded from recording. Antithrombotic therapy, however, will be documented in the case report as antithrombotic therapy from 30 days prior to obtaining consent until the end of the post-observation period.

The placement of intracranial electrodes (such as SDE or SEEG) will follow the insertion of EP-01s, but during the placement of intracranial electrodes, monopolar cautery is prohibited to minimize electrical interference, and head MRI is also not allowed after placement of EP-01s and intracranial electrodes.

#### 2.1.4 Post-insertion examinations.

(1) EP-01 Placement Confirmation: The exact anatomical location (i.e., name of blood vessel) of the EP-01 tip electrode will be confirmed by X-ray examination (frontal and lateral) immediately after insertion, 1 day after insertion, 7 days after insertion, and immediately before removal. However, if the catheter is removed before 7 days, the observation timepoints after 7 days will be omitted
(2) Adverse Event Check by CT: The presence or absence of adverse events from the EP-01 body insertion site will be confirmed by CT. Cone-beam CT (CBCT) is acceptable, as well as multislice CT, and three-dimensional (3D) data with slice thicknesses of 1 mm or less and 3–5 mm will be obtained
(3) Electroencephalography: EEG data during eye opening and closing will be recorded. The timing of obtaining EEG data during open/closed eye movements will be at the discretion of the investigator. During a seizure that displays clinical symptoms, EP-01 EEG and intracranial electrode EEG will both be clearly recorded. The first seizure occurring after implantation will be analyzed. In this protocol, the use of an extracranial referential electrode at Fpz is recommended; however, each institution may determine the referential electrode to be used, provided that it allows for stable eEEG recording.

#### 2.1.5 Outcomes

***Primary endpoint.*** The primary endpoint is comparison of the lateralization diagnostic capability of EP-01 for epileptic foci with that of intracranial electrodes. Clinical findings, imaging findings obtained within one year prior to the date of consent (non-invasive findings), and EEG findings from scalp electrodes will be provided to a third party by the Central Review Committee. For intracranial electrodes, the determination of the epileptic foci will be based on ictal intracranial EEG findings. The lateralization diagnostic results of EP-01 for epileptic foci will then be compared with those obtained from intracranial electrodes, and the percentage agreement will be calculated.

**Secondary endpoints.**

(1) Diagnostic performance of the EP-01 alone, without reliance on intracranial electrodes, for the diagnosis of lateralization of the epileptic focus. To be obtained by third-party evaluation via the Central Evaluation Committee.
(2) If there is a period during which only EP-01 is placed, comparative evaluation will be conducted between the EEG measurement performance with EP-01 alone and with both EP-01 and intracranial electrodes in place, to ensure that there is no mutual interference.
(3) To determine whether the waveforms detected by EP-01 are brain-generated EEG components, EEG changes with eyes open and closed will be observed. Evaluation will be conducted by a third party appointed by the Central Review Committee.
(4) In the case of intracranial electrodes placed bilaterally, the percentage of agreement on lateralization of the epileptic focus between EP-01 EEG with noninvasive findings and findings from bilateral intracranial electrode EEG with noninvasive findings will be compared.
(5) In the case of intracranial electrodes placed bilaterally, the percentage of agreement on lateralization of the epileptic focus between EP-01 EEG without noninvasive findings and findings from bilateral intracranial electrode EEG without noninvasive findings will be compared.
(6) Diagnostic performance for the lateralization of the epileptic focus by EP-01 (percentage agreement with the results of diagnosis by intracranial electrodes with noninvasive findings) with noninvasive findings as evaluated by the primary investigators.
(7) Epilepsy surgery outcome will be evaluated by the International League Against Epilepsy (ILAE) seizure outcome classification 1 year after focal resection.
(8) Success rate of EP-01 implantation, calculated as the number of EP-01s implanted immediately after implantation/ number of microcatheters implanted x 100. In addition, whether the EP-01 has migrated from the target vessel will be evaluated immediately before removal.
(9) Safety evaluations:
  a Severity and temporal characteristics of adverse events (including medical procedures for implantation and removal of EP-01) and adverse events judged from imaging studies, etc., attributable to the study device (e.g., hemorrhage, midline shift, CSF leakage) (Each Primary Investigator evaluate whether the adverse events are attributable to EP-01 or invasive electrodes and ultimately compare which device is responsible for the adverse events)
  b Incidence and timing of adverse events (period of occurrence: from the start of use of the study device to the end of the post-observation period, including the start of use of the study device and the end of the post-observation period)
  c Associations with all study devices (EP-01 body and EP-01 accessories), medical procedures for EP-01 implantation and removal, and other devices used in the study, judged by extent, severity, treatment, and outcome
(10) Other evaluations:
  a Failure of EP-01 (EP-01 itself and EP-01 accessories)
  b Other failures of investigational devices (EX-01 extension wire/electroencephalograph)

#### 2.1.6 *Sample size determination.*

The sample size is set at 37 using the following rationale: the null hypothesis is p = 0.8 and the alternative hypothesis is p > 0.8 for the statistical analysis of the lateralization diagnosis of EP-01 to intracranial electrode insertion concordance ratio. The hypothesis will be tested using an exact one-tailed test of binomial proportions. Assuming an expected percentage agreement of 0.97, a significance level of 0.025, and a power of 90%, the required number of cases is 34. Considering a 10% dropout rate, the target number of cases will be increased to 37.

#### 2.1.7 *Target study population.*

This trial aims to test the efficacy and safety of the EP-01 in patients with intractable epilepsy. Enrolled patients deemed “EP-01 EEG test-initiated participants” are those who are enrolled in the study and begin EEG testing with the EP-01. “EP-01 EEG test-completed participants” are those who complete both the test AND the extended study period. “Discontinued participants” are those who discontinue during the EEG test period or post-observation period for any reason. Cases that have undergone partial resection and have completed the evaluation at 1 year after resection are defined as “epileptic seizure outcome evaluation completed” and cases that are discontinued before the evaluation of the first year after completion of the study period are defined as “discontinued cases during the epileptic seizure outcome evaluation period”. Patients who fail to enroll after consent is obtained are referred to as “ineligible cases,” and patients who fail to start EEG testing with the EP-01 for any reason after enrollment are defined as “dropout cases”.

Enrollment will be primarily based on pre-trial medical evaluations and the requirements of the insertion procedure. Essentially, patients diagnosed with focal-onset seizures (based on clinical and MRI findings, and scalp electrode results) and a vascular anatomy that allows the EP-01 to be guided to a vessel close to the epileptic focus (such as the cavernous sinus, transverse sinus, superior sagittal sinus, or inferior petrosal sinus) are considered eligible for EP-01 and intracranial electrode implantation. For safety reasons, these patients must have a vascular anatomy that allows the EP-01 to be guided to a vessel close to the epileptic focus, such as the cavernous sinus or inferior petrosal sinus. In consideration of the treatment burden on the participant, the examination associated with the decision to implant these EP-01s may be determined based on data obtained up to 90 days prior to obtaining consent.

### 2.2 Data collection, management, and analysis

#### 2.2.1 *Participant backgrounds.*

After obtaining consent, between 30 days and 1 day prior to the insertion of EP-01 electrode study device, the following items will be investigated at the time of registration, after confirming that the selection criteria have been met and that no exclusion criteria are violated. Participant identification codes and background information will be included in the case report form.

(1) Observables: Sex, age, blood pressure, pulse, current medical history (epilepsy classification, seizure-type classification, duration of epilepsy, etiology), complications, other medical history, previous treatment, smoking history, and alcohol consumption history
(2) Complications: Diseases that are complicating factors at the time of obtaining consent
(3) History: Diseases that occurred before consent was obtained, plus information up to 90 days prior to the date on which consent was obtained, should be investigated. However, the investigator or sub-investigator will not ask the patient to specify the period of any important illnesses such as perinatal anomalies, febrile convulsions, head trauma, psychiatric disorders, malignant tumors, etc.
(4) Previous treatments: The history of treatment (including surgical procedures) for the primary disease prior to the time of enrollment.

#### 2.2.2 *Participant pre-insertion examination information.*

To be performed 30 days to 1 day prior to the implantation of the study device. However, if the examination is performed within 90 days prior to obtaining consent, the examination is not required and the data from prior to obtaining consent should be used.

(1) Height and Weight
(2) Cervical Echocardiography
(3) Blood Tests: Platelet count, activated partial thromboplastin time (APTT), prothrombin time-international normalized ratio (PT-INR)
(4) Non-Invasive Tests: Defined as a neuropsychological examination (presence or absence of dysfunction of the left and right frontal, temporal, occipital, and parietal lobes) and an epilepsy interview. According to guidelines, the interview will be conducted to gather information on seizure frequency, seizure status, and triggers, symptoms before and during seizures, persistence of symptoms, symptoms following seizures, presence of trauma, tongue bite, urinary incontinence, post-seizure headache and post-seizure muscle pain, age at first seizure, change and transition of seizures and seizure type, last seizure, seizure and arousal relationship to sleep, post-ictal behavior, and any other pertinent details of the condition
(5) MRI and Imaging: MRI and other imaging data (including CT, ^18^F-fluorodeoxyglucose PET, ^123^I-iomazenil-SPECT, magnetoencephalogram, etc., if performed in the usual practice) for lateralization diagnosis of the epileptic focus should be obtained. Obtaining images from the same modalities that were conducted within 1 year prior to obtaining consent will be preferable, but re-imaging for study purposes will not be required
(6) Scalp Electrode Testing: Scalp electrode examination data for the lateralization diagnosis of the epileptic focus should be obtained. Testing in the same manner conducted within 1 year prior to obtaining consent will be preferable, but re-testing for study purposes will not be required
(7) MRI for Anatomical Evaluation: Simple 3D T1-weighted imaging (3D-T1WI), MR venography, or contrast-enhanced 3D-T1WI should be performed to confirm that the patient has vascular anatomy that allows the main body of the EP-01 to be guided to vessels close to the epileptic focus, such as the cavernous sinus, transverse sinus, superior sagittal sinus, or inferior petrosal sinus. If such imaging has been previously performed within 90 days prior to obtaining consent, no re-imaging will be required and the data should be collected prior to obtaining consent.

#### 2.2.3 *Follow-up.*

The EP-01 main unit is to stay implanted for up to 2 weeks (plus up to 3 days allowance) and then be removed. Intracranial electrodes will be removed according to clinical judgment and may be removed at the same time as the EP-01 unit. A follow-up period of 1 year will be conducted to evaluate the seizure-free rate after focal resection using data obtained from the EP-01 unit.

#### 2.2.4 *Statistical methods.*

The percentage agreement between the lateralization diagnosis of the epileptic focus by EP-01 or intracranial electrodes, plus the 95% confidence interval of the percentage agreement by the Clopper–Pearson method will be determined. Full Analysis Set will be the primary dataset for this analysis.

Regarding missing data, cases where measurements are successfully obtained using intracranial electrodes but not with EP-01 will be considered as discrepancies. If both the intracranial electrodes and EP-01 cannot be measured, the case will be excluded from analysis. If only one of them is measurable, it will be conservatively considered as discordant. With respect to potential confounders, since both devices will be used for all participants, we do not anticipate any confounding concerns. Therefore, baseline characteristics will not be incorporated into the primary analysis. However, if necessary, secondary analyses that account for baseline characteristics will be conducted. As for multiple comparisons, we consider this issue irrelevant as there is only one primary outcome measure. Comparisons for secondary outcomes will also include the calculation of a 95% confidence interval using the Clopper-Pearson method, similar to the primary outcome.

### 2.3 Monitoring

#### 2.3.1 *Data monitoring.*

The analysis of EEG data and device-related adverse events in this trial will be evaluated impartially by an independent review committee. The efficacy evaluation committee comprises four epilepsy specialists. Two will evaluate the EP-01 EEG, and two will assess intracranial EEG, ensuring the study remains blinded. The safety evaluation committee consists of three specialists: an epilepsy specialist, an endovascular neurosurgeon, and a radiation oncologist. Each will evaluate device-related adverse events from the perspective of their respective fields. None of the committee members have any conflicts of interest with the sponsor company.

#### 2.3.2 *Interim analysis and monitoring.*

Because of the relatively short duration of the study, no interim analyses or reports are planned. However, if critical safety information is obtained, the continuation of the trial will be reviewed.

#### 2.3.3 *Safety assessment and adverse events.*

The presence or absence of adverse events from the EP-01 body implantation site will be confirmed by CT and 3D data for slice thicknesses of 1 mm or less and 3–5 mm will be obtained. Acquired data will be submitted to the Safety Evaluation Committee. Scans will be conducted immediately after implantation, 1 day after implantation, 7 days after implantation, immediately before removal (however, if removal is performed before 7 days, the scan 7 days after implantation should be omitted), 1 day after removal, and 7 days after removal.

#### 2.3.4 *Adverse events.*

An adverse event will be deemed as any unfavorable sign, symptom, or illness (including abnormal changes in laboratory values) observed during the study examination and post-observation periods after use of the EP-01 (main unit and accessories) or other investigational device (EX-01 Extension Wire/EEG 1200 Series Neurofax Electroencephalograph). Adverse events are also defined to include abnormal fluctuations in clinical laboratory values observed during the investigational testing and post-observation periods after use of the EEG1200 Series Neurofax, regardless of the causal relationship to the investigational device. If an adverse event is observed, the investigator shall take appropriate measures. In addition, regardless of the presence or absence of a causal relationship with the investigational device, follow-up investigations shall be conducted until, in principle, the event normalizes or recovers to a level where it is no longer considered an adverse event. If an irreversible adverse event is observed due to an organic disorder, follow-up investigations should be conducted until the symptoms are stabilized or fixed. Symptoms within the range expected for the primary disease will not be treated as adverse events. If focal epilepsy resection is performed during the study period, symptoms resulting from this procedure will not be treated as an adverse event. The numbers of adverse events, adverse events attributable to the study device, serious adverse events, and serious adverse events attributable to the study device will be tabulated in terms of the number of occurrences, number of cases, and percentage of cases with adverse events.

Safety assessments will also involve the operation of the implanted device. In this study, “failure” is defined as any malfunction of the investigational device, adverse effect of the investigational device on the human body, damage, malfunction, or other general unwellness, whether due to manufacturing, delivery, storage, or use.

### 2.4 Ethics and dissemination

#### 2.4.1 *Regulatory approval.*

This clinical trial will be conducted in accordance with ethical principles based on or equivalent to the Declaration of Helsinki (latest revision), the Japanese Pharmaceuticals and Medical Devices Law, the Ministerial Ordinance on Standards for Conducting Clinical Trials of Medical Devices (Ministerial Ordinance No. 36 of the Japanese Ministry of Health, Labour and Welfare, dated March 23, 2005), and ISO 14155, and in compliance with this Clinical Trial Implementation Plan. This clinical trial will also comply with laws, regulations, and guidelines related to clinical trials. This trial has been registered in the Japan Registry of Clinical Trials (jRCT2032230720).

Institutional review board approval was obtained from all participating institutions as follows: the University of Tsukuba (approval no. 医-55), Sapporo Medical University (approval no. 36−10), Jichi Medical University (approval no. TBD), Tokyo Medical and Dental University (approval no. 2024−1001), Yokohama City University (approval no. TBD), Seirei Hamamatsu General Hospital (approval no. 医-003), and Osaka University (approval no. TBD).

#### 2.4.2 *Dissemination.*

To disseminate the findings of this eEEG study both socially and academically, we plan to present them at conferences and publish them in peer-reviewed journals. In addition, beyond determining laterality alone, it will be necessary to explore whether eEEG can also be applied to multiple foci in clinical practice. We therefore intend to pursue data sharing and validation of research outcomes through multicenter and global collaboration.

## Supporting information

S1 Protocol Protocol. (PDF)

S2 Checklist SPILIT checklist. (PDF)

S3 File Summary of IC. (DOCX)

S4 File Instructions for primary outcome measure assessment. (DOCX)
