# Supplementary material for: Endovascular EEG device prospective multicenter single-arm clinical trial to confirm efficacy and safety performance on patients with Intractable Epilepsy: The EPSILON IE trial protocol
Source: PLoS One. 2025 Oct 16;20(10):e0332387. doi: 10.1371/journal.pone.0332387 (PMC12530528; doi:10.1371/journal.pone.0332387)
Supplement: S1 Protocol — (PDF) [file pone.0332387.s001.pdf]

Endovascular EEG Device Prospective Multicenter Single-arm clinical trial to confirm efficacy and safety performance on Intractable Epilepsy patients
-EPSILON IE-

Clinical Trial Protocol

Coordinating Investigator:
Yuji Matsumaru
Department of Stroke,
University of Tsukuba Hospital

Protocol No.: EPSILON IE
Version No.: Version 2.1
Date of Preparation: May 23, 2024
Information Disclosure Number:
jRCT2032230720

[Regarding the handling of this document]
This protocol is provided only to those directly involved in the study (e.g., head of the medical institution, principal investigator, sub-investigators, collaborators, study device administrators, specified study device administrators, Investigational Review Board [IRB], and Central Evaluation Committee). Readers are therefore asked to handle the contents described in this protocol as confidential information to prevent disclosure to third parties.

This study will be conducted in accordance with the ethical principles based on the Declaration of Helsinki, the Act on Pharmaceuticals and Medical Devices, the Ministerial Ordinance on Good Clinical Practice for Medical Devices (Ministerial Ordinance No. 36; Ministry of Health, Labour and Welfare; March 23, 2005), and ISO 14155, and in compliance with this protocol.
This study will also comply with laws, regulations, and guidelines concerning clinical trials.

Additionally, the protocol will be revised if deemed necessary for the safe conduct of the study.

Preparation and Revision History

Date of Preparation	Version Number	Details of Revision
December 26, 2023	Version 1.0	First version
January 26, 2024	Version 1.1	Revision of errors in writing<br>Addition of details on data submitted to the Central Evaluation Committee
March 22, 2024	Version 2.0	Revision of errors in writing, maintenance of descriptions<br>Addition of funders, maintenance of descriptions on case handling, addition of details on concomitant medications and therapies, addition of details on adverse event handling, and addition of details on data submitted to the Central Evaluation Committee and the Safety Monitoring Committee
May 23, 2024	Version 2.1	Revision of errors in writing, maintenance of descriptions, maintenance of descriptions in accordance with ISO 14155, addition of information in accordance with ISO 14155 (risks and benefits associated with participation in the study, method for confirming accuracy management of specified study devices, and rationale for setting of endpoints)

1 PROTOCOL SYNOPSIS

Title of Study	Endovascular EEG Device Prospective Multicenter Single-arm clinical trial to confirm efficacy and safety performance on Intractable Epilepsy patients
Objectives	To evaluate the efficacy of the EP-01 endovascular EEG device in lateralization diagnosis of epileptic foci in patients with intractable epilepsy. Additionally, to evaluate safety when using EP-01.
Study Design	A multicenter, prospective, single-arm study<br>This study will be conducted at multiple sites. The study will evaluate the investigational device in a structure that includes a screening period of up to 30 days, observation period of up to 2 weeks, and post-observation period of 1 week. Additionally, patients who have undergone focal resection will be evaluated for epileptic seizure resolution rate approximately 1 year after epileptic resection.
Development Phase	Validation study
Investigation Device Identifier	Endovascular EEG device<br>Investigational device identifier: EP-01<br>The investigational device EP-01 is comprised of the EP-01 main unit and EP-01 accessories (connector and sheath set, wire winder, inserter, long sheath, wire wing, and dispenser). Per each subject, a maximum of 6 EP-01 main units and connectors depending on the case; 2 sheath sets; and a maximum of 2 wire winders, inserters, long sheaths, and wire wings as needed will be used.
Other Specified Study Devices	・ EX-01 extension wire<br>Used to temporarily extend the overall length of the EP-01 main unit when removing the microcatheter from the EP-01 main unit.<br>Device provided by: Epsilon Medical Inc.<br>・ Neurofax EEG-1200 Electroencephalograph series<br>Certification number: 218AHBZX00013000<br>Manufactured and distributed by: Nihon Kohden Corporation<br>Connected to EP-01 for EEG measurement
Subjects	Patients with intractable epilepsy undergoing intracranial electrode placement
Inclusion Criteria	At enrollment, patients who meet all of the following criteria will be selected.<br>(1) Among patients with intractable epilepsy, those with temporal lobe epilepsy, frontal lobe epilepsy, parietal lobe epilepsy, occipital lobe epilepsy, and insular gyrus epilepsy<br>(2) Patients undergoing intracranial electrode placement<br>(3) Patients with vascular anatomy that allows EP-01 to be guided into the brain from the right and left jugular veins to vessels close to the epileptic focus, such as cavernous sinus, transverse sinus, superior sagittal sinus, and inferior petrosol sinus. [The vessel into which the EP-01 main unit will be placed will be determined by the physician, but the EP-01 must be confirmed as able to be guided to a symmetrical position on both sides of the cerebral vein.]<br>(4) Patients age 15–70 years (at the time of obtaining consent)

Exclusion Criteria	At time enrollment, patients who meet any of the following criteria will be excluded.<br>(1) Patients with epilepsy who have generalized seizures only<br>(2) Patients with obstruction or thrombus/thrombi in the internal jugular vein<br>(3) Patients with a predisposition to bleeding and coagulation abnormalities<br>(4) Patients with difficulty remaining still during surgeries<br>(5) Patients with severe allergies to contrast media<br>(6) Patients with metal allergies<br>(7) Patients who are pregnant or potentially pregnant<br>(8) Patients participating in other studies<br>(9) Patients whom the principal investigator or sub-investigators determine to be inappropriate to participate in this study
Endpoints	[Primary Endpoint]<br>(1) Diagnostic performance of EP-01 for lateralization diagnosis of epileptic focus (percentage agreement with intracranial electrode-based diagnosis results): third-party evaluation by the Central Evaluation Committee.<br>• Diagnostic results on epileptic focus lateralization ("lateralization"), based on clinical findings, imaging findings obtained within 1 year prior to the date of consent, and scalp electrode-based EEG findings ("non-invasive findings") combined with EP-01-based EEG findings, will be compared with the diagnostic results on the lateralization based on non-invasive findings combined with intracranial electrode EEG findings, and the percentage agreement will be calculated.<br>[Secondary Endpoints]<br>(1) Diagnostic performance of EP-01 alone for lateralization diagnosis of epileptic focus: a third-party evaluation by the Central Evaluation Committee.<br>• Lateralization diagnosis results on epileptic focus based on EP-01 EEG findings alone compared with the lateralization diagnosis results based on intracranial electrode EEG findings, and the percentage agreement.<br>(2) EEG Identification of EP-01 Waveforms<br>• Evaluation of whether waveforms detected by EP-01 are due to EEG, comparing with EEG findings when the eyes are opened and closed: third-party evaluation by the Central Evaluation Committee.<br>• If EP-01 alone was placed for a period, comparative evaluation of EEG measurement performance of EP-01 alone and EP-01 combined with inserted intracranial electrodes: evaluation by the principal investigator.<br>(3) Diagnostic performance of EP-01 for lateralization diagnosis of epileptic focus (percentage agreement with diagnostic results of intracranial electrodes placed on both sides): third-party evaluation by the Central Evaluation Committee.<br>• Lateralization diagnosis results on epileptic focus based on EP-01 EEG findings and non-invasive findings compared with the lateralization diagnosis results based on bilaterally-placed intracranial electrode EEG findings and non-invasive findings, and the percentage agreement.<br>• Lateralization diagnosis results for epileptic focus based on EP-01 EEG findings alone compared with the lateralization diagnosis results based on bilaterally-placed intracranial electrode EEG findings only, and the percentage agreement.<br>(4) Diagnostic performance of EP-01 for lateralization diagnosis of epileptic focus (percentage agreement with diagnostic results of intracranial electrodes): evaluation by the principal investigator.<br>• Lateralization diagnosis results based non-invasive findings combined with EP-01 EEG findings compared with lateralization diagnosis results based on non-invasive findings combined with intracranial electrode EEG findings, and the percentage agreement.<br>(5) Resolution rate of epileptic seizures 1 year after focal resection.<br>• Evaluated using the International League Against Epilepsy (ILAE)

	classification.<br>(6) Success rate of EP-01 placement (%).<br>• The number of placed EP-01 main units immediately after placement / number of placed microcatheters multiplied by 100 is calculated.<br>• Assessment of any migration of EP-01 evaluates from the intended vessel of placement immediately prior to removal.<br>(7) Safety Evaluation<br>• Serious adverse events attributable to the investigational device (including medical acts for placement and removal of EP-01) and adverse events as determined by imaging testing*: third-party evaluation by the Safety Monitoring Committee<br>*Adverse events determined by imaging testing: hemorrhage, midline shift, cerebral spinal fluid leak<br>• Occurrence of adverse events [Incidence period: from the start of the use of the investigational device to the end of the post-observation period. Relation to all investigational devices (EP-01 main unit and EP-01 accessories), relation to medical acts for EP-01 placement and removal, relation to other specified devices, extent, seriousness, treatment, and outcome].<br>(8) Other Evaluations<br>• Failures of the investigational device EP-01 (failures of EP-01 main unit and EP-01 accessories)<br>• Failures of other specified study devices (EX-01 extension wire / electroencephalograph)
Test Method	As a general rule, EP-01 is placed at the same time as the Wada test or intracranial electrode placement. Note that if the placement of the EP-01 main unit into the cerebral vein and the placement of intracranial electrodes are performed on separate days, they will be performed within 3 days. Subjects are observed for up to 2 weeks until seizure symptoms occur, and the EP-01 main unit is removed after the foci lateralization is confirmed. Lateralization the epileptic focus is diagnosed based on EEG waveforms obtained from intracranial electrodes and EP-01.
Restrictions on concomitant use, prohibitions on concomitant drugs and medications	In line with the intracranial electrode placement, drugs are discontinued or reduced as is usually done in regular clinical practice at the physician's discretion on an as-needed basis.
Planned Number of Subjects	37
Planned Study Period	Investigational device evaluation period<br>March 2024 to August 2025<br>Period including evaluation period of epileptic seizure outcomes following epilepsy focal resection<br>March 2024 to August 2026

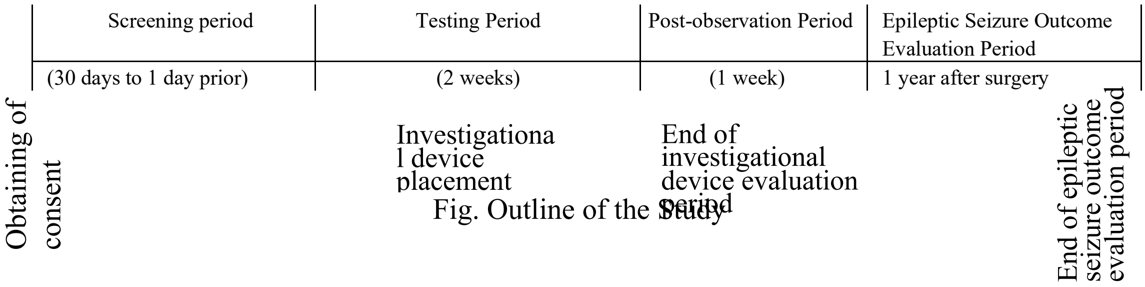

Fig. Outline of the Study

Table. Test/Observation Items and Schedule

Item	Screening Period	Investigational Device Evaluation Period								Epileptic Seizure Outcome Evaluation
		Testing Period						Post-observation Period		
Timepoint	Screening		Investigational device placement[10]	1 day after investigational device placement	Intracranial electrode placement	1 week after investigational device placement	Investigational device removal[11]/ discontinuation[15]	1 day after investigational device removal	1 week investigational device removal / discontinuation[15]	1 year after surgical treatment[12]
Acceptable Range	30 days to 1 day prior to investigational device placement				Within ±3 days from investigational device placement		Within 14 days after investigational device placement[13]	+3 days	+3 days	±60 days
Obtaining of consent	●									
Enrollment		●								
Confirmation of eligibility	●									
Examination of Subject Background[1]	●									
Height/weight[2]	●									
Cervical ultrasonography[3]	●									
Blood test[4]	●									
Non-invasive findings[5]: Clinical findings on epileptic seizures	●									
Non-invasive findings[5]: Image findings	●									
Non-invasive findings[5]: Scalp electrode	●									
Image test: MRI test[6]	●									
Image test: X-ray test[7]			●	●		●	●			
Image test: CT[8]	○[14]		●	●		●	●	●	●	
EEG test[16]			◄	—	—	—	►			
Adverse events			◄	—	—	—	—	—	►	
Failures			◄	—	—	—	►			
Primary disease treatment / antithrombotic therapy[9]	◄	—	—	—	—	—	—	—	►	
Concomitant medication			◄	—	—	—	—	—	►	
Epileptic seizure evaluation (ILAE classification)										●

●: Data or observation items required in this study ○: Performance not required for the purpose of this study

1) Information on age, sex, blood pressure, pulse, current medical history, complications, other medical history, smoking history, and alcohol consumption history is collected at the time consent is obtained.

2,3) If they have been performed within 90 days prior to obtaining consent, no re-testing is required, and data prior to obtaining consent is collected.

4) If it is performed within 90 days prior to obtaining consent, no repeated performance is required, and data prior to obtaining consent is collected. Platelet count, activated partial thromboplastin time (APTT), and prothrombin time-international normalized ratio (PT-INR) tests are performed.

5) Depending on the subject's condition, only those necessary for lateralization diagnosis of the epileptic focus are investigated at the discretion of the principal investigator.

6) Simple 3D T1-weighted imaging (3D-T1WI) and either magnetic resonance venography (MRV) or contrast-enhanced 3D-T1WI are performed. If they are performed within 90 days prior to obtaining consent, no re-measurements are required, and data prior to obtaining consent is collected.

7) X-ray tests (frontal and lateral) are performed. The tests are performed immediately after placement of the investigational device, the day after placement of the investigational device, 1 week after placement of the investigational device (unnecessary if seizures occur within 1 week from placement of the investigational device), and immediately before removal of the investigational device.

8) Imaging methods immediately after EP-01 placement are not limited to multislice computed tomography (CT) but also include cone-beam computed tomography (CBCT). The CT tests are performed immediately after placement of the investigational device, the day after placement of the investigational device, 1 week after placement of the investigational device (unnecessary if seizures occur within 1 week from placement of the investigational device), and immediately before removal of the investigational device. 3D data of slice thickness of 1 mm or less and 3–5mm is submitted to the Safety Evaluation Committee.

9) Information on treatment (including surgical procedures) and antithrombotic therapy for the primary disease is collected from before consent is obtained and until the end of the post-observation period.

10) As a general rule, the investigational device is placed during the same procedure as the Wada test or intracranial electrode placement.

11) The investigational device is placed until the occurrence of an epileptic seizure or for a maximum of 14 days (+3 days). The investigational device may be removed on the same day as the intracranial electrode removal or surgical treatment. If a shift in the tip position of the investigational device is suspected, an x-ray test is promptly performed to determine whether or not to remove.

12) Performed only on subjects who underwent epileptic resection.

13) If it is difficult to remove within up to 2 weeks due to the system of respective medical institutions, an allowance of +3 days may be set.

14) Only if it is performed within 90 days prior to the placement of the investigational device in regular medical care, it is provided to the Safety Evaluation Committee. If it is not performed, re-imaging is not required.

15) In the case of discontinuation during the testing period, depending on the subject's situation, the investigational device is removed and the subject is moved to the post-observation period after performing, as much as possible, tests specified at investigational device removal. In the case of discontinuation during the post-observation period, depending on the subject's situation, the subject is withdrawn after performing, as much as possible, tests specified at 1 week after investigational device removal.

16) During the period when only EP-01 or both EP-01 and intracranial electrodes are placed, EEG with EP-01 and the intracranial electrodes are measured under video surveillance. EEG data when the eyes are opened and closed (the timing of acquiring EEG data when the eyes are opened and closed is determined by the principal investigator) and EEG data during epileptic seizures are submitted to the Central Evaluation Committee.

2 TABLE OF CONTENTS

1 PROTOCOL SYNOPSIS 3
2 TABLE OF CONTENTS 8
3 List of abbreviations and definitions of terms 12
4 HISTORY OF DEVELOPMENT 13
4.1 Background of Development 13
4.2 Clinical Positioning of the Device 13
4.3 Non-clinical Study Results 15
4.4 Clinical Study Results 15
4.5 Known and Potential Risks and Benefits to Subjects 15
4.5.1 Risks When Using the Investigational Device EP-01 15
4.5.2 Risks Associated with Subjects' Participation in the Study 16
4.5.3 Summary of Known and Potential Risks and Benefits to Subjects 16
5 PURPOSE OF THIS STUDY 16
6 SELECTION OF SUBJECTS 17
6.1 Subjects 17
6.2 Inclusion Criteria 17
6.3 Exclusion Criteria 17
7 SPECIFIED STUDY DEVICES 18
7.1 Investigational Device 18
7.1.1 Name or Identifier of the Investigational Device 18
7.1.2 EP-01 Main Unit 18
7.1.3 EP-01 Accessories 18
7.1.4 Packaging and Labeling of the Investigational Device 19
7.2 Procedures for Delivery, Management, and Release of Investigational Device 19
7.2.1 Precautions for Handling of Investigation Device 19
7.2.2 Disposal of the Investigation Device 19
7.3 Other Specified Study Devices 19
7.3.1 EX-01 Extension Wire (Epsilon Medical Inc.) 19
7.3.2 Neurofax EEG-1200 Electroencephalograph series (Nihon Kohden Corporation) 20
8 SUBJECT ENROLLMENT 21
8.1 Enrollment Procedure 21
8.2 Definitions of Subjects Assigned in the Study 21
9 STUDY METHOD 22
9.1 Study Design 22
9.2 Diagnosis 22
9.3 Application and Placement Duration of the Investigational Device 22
9.3.1 Method of Investigational Device Placement 22
9.3.2 Method of Intracranial Electrode Placement 23
9.3.3 Timing of Investigational Device Placement 23
9.3.4 During the Investigational Device Placement Period 23
9.3.5 Removal of Investigational Device 23
9.3.6 Removal of Intracranial Electrodes 23
10 SUBJECT MANAGEMENT 24
10.1 Prior Treatment Drugs and Therapies 24
10.2 Concomitant Drugs and Therapies 24
10.2.1 Antithrombotic Therapy 24
10.2.2 Prohibited or Restricted Concomitant Drugs and Therapies 24
10.2.3 Concomitant Drugs and Therapies Requiring Caution 24
11 TEST/OBSERVATION PARAMETERS AND SCHEDULE 25

11.1 Observation Parameters 25
11.1.1 Examination of Subject Background 25
11.1.2 Height and Weight 25
11.1.3 Cervical Ultrasonography 25
11.1.4 Blood Test 25
11.1.5 Non-invasive Findings 25
11.1.6 MRI Test 26
11.1.7 Confirmation of Location of Microcatheter Placement 26
11.1.8 Confirmation of Location of EP-01 Placement 26
11.1.9 CT Scan 26
11.1.10 EEG Test 27
11.1.11 Observation of Adverse Events 27
11.1.12 Observation of Failures 28
11.1.13 Concomitant Drugs and Therapies 28
11.1.14 ILAE Classification 28
11.1.15 Evaluation by the Central Evaluation Committee 28
11.1.16 Evaluation by the Safety Evaluation Committee 29
11.2 Test/Observation Parameters and Schedule 29
12 ENDPOINTS 32
12.1 Primary Endpoint 32
12.2 Secondary Endpoints 32
12.2.1 Diagnostic Performance of EP-01 Alone for Lateralization Diagnosis of Epileptic Focus: Third-party Evaluation by the Central Evaluation Committee 32
12.2.2 EEG Identification of EP-01 Waveforms 32
12.2.3 Diagnostic Performance of EP-01 for Lateralization Diagnosis of Epileptic Focus (Percentage Agreement with Intracranial Electrode-based Diagnosis Results): Third-party Evaluation by the Central Evaluation Committee 33
12.2.4 Diagnostic Performance of EP-01 for Lateralization Diagnosis of Epileptic Focus: Evaluation by the Principal Investigator and Others 33
12.2.5 Seizure Resolution Rate 1 year After Focal Resection 33
12.2.6 Success Rate of EP-01 Placement (%) 33
12.2.7 Safety Evaluation 34
12.2.8 Other Evaluations 34
13 HANDLING OF ADVERSE EVENTS AND FAILURES 35
13.1 Response to Subjects at Occurrence of Adverse Events 35
13.2 Reporting of Serious Adverse Events and Failures 35
13.2.1 Definition of Serious Adverse Events and Failures That May Cause Serious Health Hazards. 35
13.2.2 Serious Adverse Events and Failures Subject to Reporting 36
13.2.3 Procedures for Serious Adverse Event and Failure Reporting 36
13.2.4 Reporting by the Coordinating Investigator to the Study Device Provider 36
13.3 Reporting of Other Adverse Events 36
13.4 Case Reports on Failures and Adverse Events of Other Specified Study Devices (EX-01 extension wire and EEG-1200 Electroencephalograph series) 37
14 HANDLING OF DEVIATIONS FROM THE PROTOCOL 38
15 DISCONTINUATION CRITERIA AND PROCEDURE 39
15.1 Termination of the Study 39
15.2 Discontinuation or Suspension of the Study 39
15.3 Policy on Measures for Subjects After Study Discontinuation and Termination 39
15.4 Study Dropout and Discontinuation Criteria for Individual Subjects 39
15.5 Discontinuation of the Study 40
16 STATISTICAL ANALYSIS 41
16.1 Determination of Target Sample Size 41

16.2 Data Handling Standards 41
16.3 Population Subject to Analysis 41
16.3.1 Safety Analysis Set (SAF) 41
16.3.2 Efficacy Analysis Set 41
16.4 Analysis of Subject Background 41
16.5 Analysis of Primary Endpoint 41
16.6 Analysis of Secondary Endpoints 42
16.6.1 Efficacy Secondary Endpoints 42
16.6.2 Safety Secondary Endpoints 42
16.7 Interim Analysis 42
17 COMPLIANCE WITH AND REVISION OF THE PROTOCOL 43
17.1 Compliance With and Deviation or Change From the Protocol 43
17.2 Revision of the Protocol 43
18 QUALITY CONTROL AND QUALITY ASSURANCE OF THE STUDY 44
18.1 Quality Control 44
18.1.1 Direct Viewing of Source Documents 44
18.1.2 Identification of Source Documents 44
18.1.3 Data Management 44
18.1.4 Monitoring 44
18.2 Quality Assurance 44
19 ETHICS 45
19.1 Ethical Conduct of the Study 45
19.2 Protection of Subject Confidentiality 45
19.3 Explanation to and Consent of Subjects and Legal Representatives 45
19.3.1 Consent Form and Other Informational Forms 45
19.3.2 Informational Form for Assent and Other Informational Forms 46
19.3.3 Contents of Informational Form and Assent Form 46
19.3.4 Timing and Method of Obtaining Consent and Assent 46
19.3.5 Considerations Concerning Consent 47
19.3.6 Revision of Informational Form for Consent and Assent 47
19.4 Investigational Review Board 47
19.5 Central Evaluation Committee 47
19.6 Safety Evaluation Committee 48
19.7 Conflict of Interest 48
19.7.1 Provision of Research Funds 48
19.7.2 Provision of Investigational Device 49
19.7.3 Provision of Data Acquired in the Study 49
20 RECORDS RELATED TO THE STUDY AND RETENTION 50
20.1 Case Report Form 50
20.1.1 Format of the Study's Case Report Form 50
20.2 Retention of Records 50
20.2.1 Principal Investigator 50
20.2.2 Medical Institution 50
21 COMPENSATION FOR HEALTH DAMAGE IN SUBJECTS AND REDUCTION OF BURDEN OF STUDY PARTICIPATION 51
21.1 Compensation for Health Hazards 51
21.2 Reduction of Subject Burden of Study Participation 51
22 ARRANGEMENTS ON PUBLIC DISCLOSURE 51
23 PLANNED STUDY PERIOD 51
24 REFERENCE 52
25 APPENDIX 53
25.1 Appendix 1. ILAE Classification 53

<Table of Tables>

Table 7-1. List of EP-01 Accessories 18
Table 11-1. Evaluation Items of the Central Evaluation Committee........ 28
Table 11-2. Evaluation Items of the Safety Evaluation Committee 29
Table 11-3. Test/Observation Parameters and Schedule 30
Table 19-1. Evaluation Items of the Safety Evaluation Committee 48

<Table of Figures>

Fig 9-1. Outline of the Study 22

3 List of abbreviations and definitions of terms

Abbreviation/Term	English	Japanese
CBCT	Cone Beam Computed Tomography	コーンビームコンピュータ断層撮影
CT	Computed Tomography Scan	コンピュータ断層撮影
JIS	Japanese Industrial Standards	日本産業規格
MEG	Magneto Encephalo Graphy	脳磁図記録
MRI	Magnetic Resonance Imaging	磁気共鳴画像診断
MRV	Magnetic Resonance venography	磁気共鳴静脈撮像検査
PET	Positron Emission Tomography	陽電子放出断層撮影
SDE	Subdural Electrodes	硬膜下電極
SEEG	Stereo-Electroencephalography	定位的頭蓋内脳波
IMZ SPECT	^{123}I-iomazenil single photon emission computed tomography	イオマゼニル単一光子放射型<br>コンピュータ断層撮影

4 HISTORY OF DEVELOPMENT

4.1 Background of Development

The EP-01 endovascular EEG is applicable to intractable epilepsy. (*1) EP-01 is a single-use medical device that is inserted into cerebral veins for EEG detection to diagnose focal lateralization, which is important for focal resection performed on patients with intractable epilepsy.

*1 Definition of intractable epilepsy
Intractable epilepsy in the medical sense is defined as a condition in which seizures remain uncontrolled for at least 1 year and interfere with daily life even after at least 2 years of treatment with sufficient doses of a major antiepileptic drug as a monotherapy or 2 to 3 types of drugs as a combination therapy. However, the definition of intractable epilepsy is not absolute and differs according to purpose of use and situation. For example, socially intractable epilepsy in adults under the Act on Road Traffic is defined as conditions that do not achieve suppression of seizures for 2 years or longer despite appropriate pharmacotherapy.[1)]

The number of patients with epilepsy in Japan is estimated to be approximately 1 million,[2)] of whom approximately 20% have intractable epilepsy which is unresponsive to pharmacotherapy.[1)] With intractable epilepsy, since antiepileptic drugs cannot alleviate seizure symptoms, surgical resection of the epileptic foci is considered for patients with seizure symptoms that affect their daily lives. Currently, magnetic resonance imaging (MRI), CT, single-photon emission computed tomography (SPECT), and scalp electrodes are first used to identify the foci.[3)] However, it is often difficult to identify the foci with these, and intracranial electrode placement by craniotomy is therefore used for definitive diagnosis of foci identification.[3),4)] Intractable epilepsy is treated by identifying the epileptic focus and then surgically removing the focus site. In determining the extent of resection, it is important to differentiate between areas that should be resected and areas that should not. It is therefore important to diagnose the focus site based on EEG using intracranial electrodes. The purposes of intracranial electrodes include: 1. estimation of the epileptogenic area, and 2. functional mapping.[4)] The physician appropriately determines the extent of focal resection by leveraging these characteristics of intracranial electrodes. Furthermore, there are two types of intracranial electrodes: subdural electrodes (SDE) and stereo-electroencephalography (SEEG) with deep electrodes.[3),4)] SDE covers the surface of the brain "as a plane" and are placed subdurally by craniotomy. SEEG, on the other hand, uses a stereotactic technique to insert electrodes deep into the brain and records EEG "as specific points." SDE enables easy functional mapping but is highly invasive compared to SEEG. Consequently, SEEG is more applicable in re-operative cases where SDE placement is difficult or where electrode placement in both hemispheres is desirable to determine the lateralization of the epileptic focus.[3),4)] These intracranial electrodes are considered the golden standard for determining the epileptic focus and extent of resection[1)] and are a useful method for determining the extent of resection in epileptic focal resection.

Among epileptic focal resections, hippocampal and amygdalar resection for temporal lobe epilepsy is known to be associated with a high rate of improvement, with a response in 90% or more cases.[7),11),12)] As aforementioned, preoperative focal diagnosis is extremely important when performing resection. Intracranial EEG recording is a highly accurate focal diagnostic method, with the focus being the preceding site of onset of seizure waves when seizures start.[9)] Although SEEG is suitable for lateralization diagnosis, in Japan, SDE is sometimes used for lateralization diagnosis because the dedicated small-diameter electrodes and anchor bolts used in SEEG have not received regulatory approval,[5),6)] causing a delay in clinical use. Meanwhile, efforts to avoid intracranial electrodes are continuing since they are invasive.[7)] It is difficult to perform surgeries in cases where the focal site is unknown and the area requiring craniotomy is too large. Additionally, it is known that lateralization diagnosis results based on EEG from scalp electrode may differ from lateralization diagnosis results based on that from intracranial electrodes,[10)] and it is very important to make a definitive diagnosis on lateralization based on EEG at sites closer to the cerebral parenchyma.

4.2 Clinical Positioning of the Device

The EP-01 is being developed with the aim of making EEG acquisition extremely minimally invasive compared to intracranial electrodes, since it approaches from within blood vessels and measures EEG from within the cerebral veins. At this point in time, since EP-01 is monopolar, it unrealistic to perform functional mapping, one of the two purposes of intracranial electrodes. However, it is anticipated that EP-01 may enable lateralization diagnosis, the other purpose of intracranial electrodes. Cases where lateralization diagnosis is particularly effective is temporal lobe epilepsy cases. Intracranial electrodes have been placed in the past for lateralization diagnosis, with the reportedly less invasive SEEG being considered suitable.[4)]

Since 1960s, lateralization diagnosis has been performed using SEEG[9)], and lateralization can be diagnosed even with a small number of electrodes. It is therefore believed that lateralization diagnosis is also possible with EP-01. In a previous Japanese case where wire-form endovascular EEG electrodes were placed in the cerebral artery,[13)] simultaneous placement of intracranial electrodes and endovascular EEG electrodes in a patient with temporal lobe epilepsy yielded similar seizure spikes from both. In this case, there was only 1 endovascular EEG electrode (number of electrode: 1). Furthermore, in an EP-01 specified clinical research conducted by this study's investigators to investigate the acute-phase efficacy in humans, it was confirmed that EP-01 can acquire EEG and determined that EP-01 can very likely detect epileptic seizure spikes alike intracranial electrodes and diagnose lateralization.
The expected changes in the treatment algorithm with and without EP-01 are shown below with reference to the flow of current epilepsy treatment.[3)]

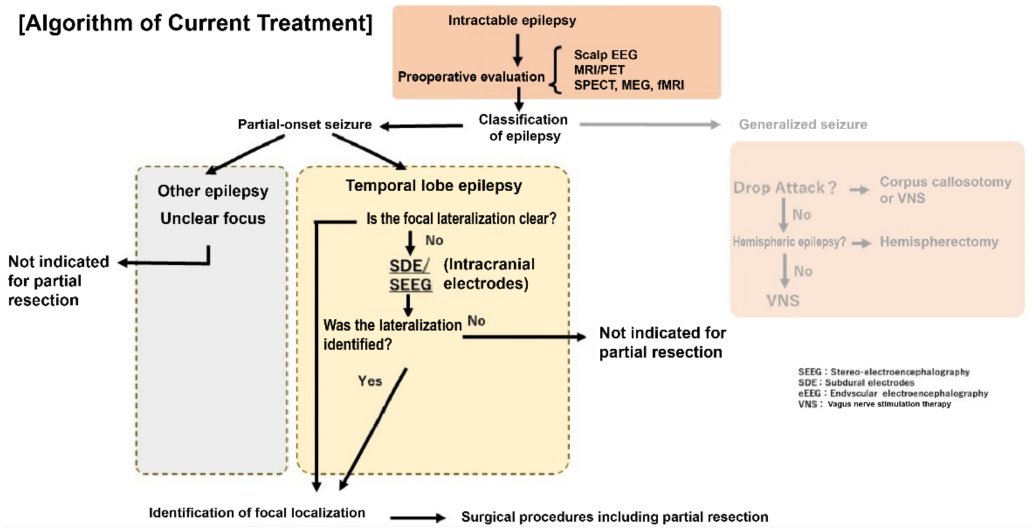

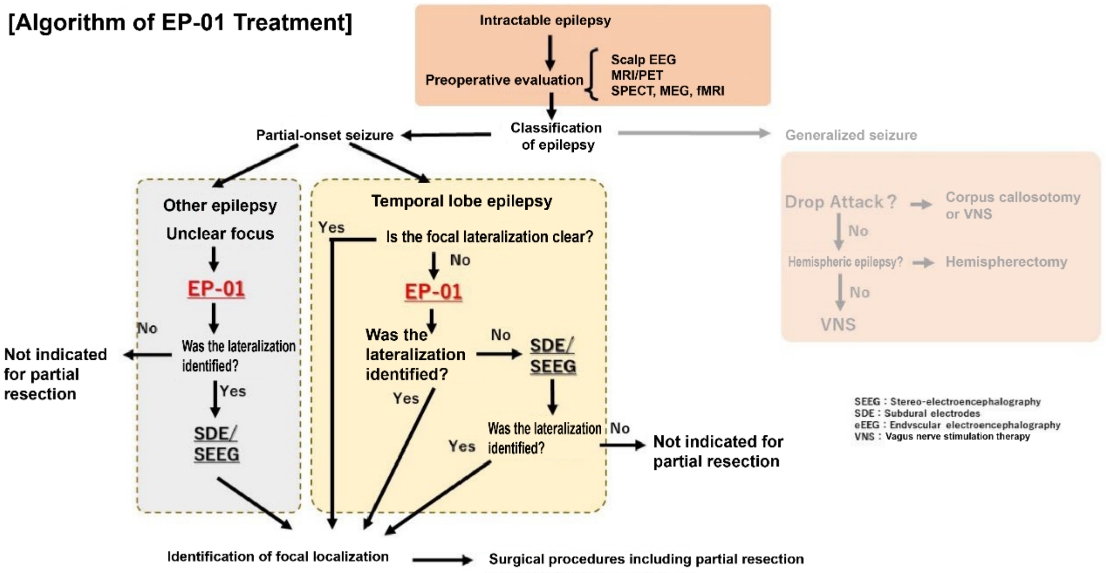

Among intractable epilepsy, those for which partial focal resection is considered is a condition called *partial-onset seizure*, and partial-onset seizures are further divided into two types, *temporal lobe epilepsy* and *other epilepsy*, according to the focal location
In *temporal lobe epilepsy*, if the lateralization of the focus is diagnosed, the extent of resection is predetermined in the form of surgical method[14)], and partial resection can be performed based on the lateralization diagnosis. If EP-01 can be used to diagnose focal lateralization in *temporal lobe epilepsy*, it could replace intracranial electrodes. If it does not achieve lateralization diagnosis, conventional intracranial electrodes can be used for lateralization diagnosis.

On the other hand, in *other epilepsy* (frontal lobe epilepsy, occipital lobe epilepsy, parietal lobe epilepsy, insular gyrus epilepsy), the focal location needs to be identified with some method and then resected. (The extent of resection is not predetermined as in temporal lobe epilepsy.) Thus, when attempting to identify the focus using current intracranial electrodes, an extremely large-scale craniotomy or a large number of SEEG need to be performed. This is highly invasive, and physicians often hesitate to perform it in real clinical practice. If the focal lateralization is diagnosed with EP-01, intracranial electrodes need to be placed only in the hemisphere concerned, thus reducing the invasiveness of the intracranial electrode placement. In intractable epilepsy cases where partial resection is difficult, the use of this product enables partial resection and may lead to the cure of intractable epilepsy.

4.3 Non-clinical Study Results

The following tests were conducted to confirm that the investigational device has characteristics suitable for clinical specifications, and conformity with all criteria standards was confirmed. For details, refer to the summary on the study devices.

- Physical/chemical characteristics
- Electrical safety and electromagnetic compatibility
- Biological safety
- Stability
- In-vitro performance test (simulated use test)
- In-vivo performance testing (animal studies)
- Other

4.4 Clinical Study Results

The investigational device's efficacy and safety in the acute phase was investigated in the Feasibility Clinical trial of endovascular Electroencephalogram Measurement Device ("eEEG feasibility trial") (jRCT: jRCTs032220543). In the eEEG feasibility trial, the investigational device was temporarily placed for 1 hour in 5 patients, and comparison with EEG of scalp electrodes and the occurrence of adverse events up to 1 week after placement were confirmed. All 5 cases were completed, and it was confirmed that the signals acquired were EEG in all of the cases.
Furthermore, no adverse events or failures attributable to the device or procedures during surgeries and up to 1 week after the surgeries have been confirmed, posing no safety concerns at this point in time.

4.5 Known and Potential Risks and Benefits to Subjects

4.5.1 Risks When Using the Investigational Device EP-01

Risk management activities for EP-01 are conducted in accordance with ISO 14971.
The use of EP-01 may cause failures and adverse events including but not limited to the following.

1) Failures

- Damage, deformation, separation in device
- Inability to measure EEG
- Inability to reach intended site
- Corrosion

2) Adverse events

- Hemorrhage
- Vascular dissection
- Intimal peeling
- Angiospasm
- Vascular perforation
- Thrombosis (including pulmonary embolism)
- Remnant of investigational device
- Injury from investigational device
- Difficulty in removal
- Infectious disease
- Metal allergy reaction
- Contrast media allergy reaction
- Hematoma at puncture site

- Pseudoaneurysm
- Cerebral infarction
- Renal dysfunction
- Epileptic seizure
- Restlessness
- Pyrexia

4.5.2 Risks Associated with Subjects' Participation in the Study

The X-ray test performed for the insertion and removal of the study devices may pose risks of skin and eye disorders associated with radiation exposure. However, the duration of radiation exposure due to placement of the study devices is comparable to that of endovascular tests performed in routine medical care, and therefore its risks are thought to be equivalent to that of tests in routine medical care. If the study device is performed at the same time as the Wada test, additional anesthetics are used. The potential risks of anesthetics include nausea, vomiting, headache, and chills/fever. Additionally, while contrast-enhanced X-ray tests are performed as part of routine medical care during Wada tests, participation in this study is expected to increase the amount of contrast media used and prolong the duration of radiation exposure. However, the amount of contrast media used and the duration of radiation exposure in this study are comparable to those of endovascular tests performed in routine medical care, and the risks are similar to those of such tests.
If the study device is inserted at the same time as the placement of the intracranial electrodes, an additional contrast-enhanced x-ray test is performed. Therefore, in addition to the risks associated with the placement of intracranial electrodes, there are risks associated with contrast media and radiation exposure similar to those in endovascular tests. Since anesthetics are also used as part of routine medical care during the placement of intracranial electrodes, the risks associated with anesthetics are comparable to those in routine medical care.
From the above, the risks associated with participation in the study in addition to the risks associated with the use of the investigational device described in 4.5.1 are considered to be comparable to those of tests performed in general clinical practice.

4.5.3 Summary of Known and Potential Risks and Benefits to Subjects

Although participation in the study confers no anticipated direct benefits for the subjects themselves, as described in 4.1 Background of Development, the results obtained from this study may render endovascular EEG testing using the investigational device comparable to intracranial electrode-based EEG testing and may, in the future, enable patients with intractable epilepsy to receive epileptic surgical treatment without intracranial electrode-based EEG testing.

5 PURPOSE OF THIS STUDY

To evaluate the efficacy of the EP-01 endovascular EEG device in lateralization diagnosis of epileptic foci in patients with intractable epilepsy. Additionally, to evaluate safety when using EP-01.

6 SELECTION OF SUBJECTS

6.1 Subjects

Patients with intractable epilepsy undergoing intracranial electrode placement

[Rationale]
To compare the diagnostic performance for lateralization diagnosis of the epileptic focus to that of intracranial electrodes, subjects were defined as those scheduled to receive intracranial electrode placement for partial resection of epileptic foci.

6.2 Inclusion Criteria

Patients who meet all of the following criteria at the time of enrollment will be selected.

(1) Among patients with intractable epilepsy, those with temporal lobe epilepsy, frontal lobe epilepsy, parietal lobe epilepsy, occipital lobe epilepsy, and insular gyrus epilepsy
(2) Patients undergoing intracranial electrode placement
(3) Patients with vascular anatomy that allows EP-01 to be guided into the brain from the right and left jugular veins to vessels close to the epileptic focus, such as cavernous sinus, transverse sinus, superior sagittal sinus, and inferior pyramidal sinus. [The vessel into which the EP-01 main unit will be placed will be determined by the physician, but the EP-01 must be confirmed as able to be guided to a symmetrical position on both sides of the cerebral vein.]
(4) Patients age 15–70 years (at the time of obtaining consent)

[Rationale]
(1) (2): Defined as subject diseases.
(3): Defined in an ethical manner.
(4): Defined for safety reasons, though intracranial electrodes are indicated in children.

6.3 Exclusion Criteria

Patients who meet any of the following criteria at the time of enrollment will be excluded.

(1) Patients with epilepsy who have generalized seizures only
(2) Patients with obstruction or thrombus/thrombi in the internal jugular vein
(3) Patients with a predisposition to bleeding and coagulation abnormalities
(4) Patients with difficulty remaining still during surgeries
(5) Patients with severe allergies to contrast media
(6) Patients with metal allergies
(7) Patients who are pregnant or potentially pregnant
(8) Patients participating in other studies
(9) Patients whom the principal investigator or sub-investigator determines to be inappropriate to participate in this study

[Rationale]
(1): Defined to further clarify subject disease.
(2) to (8): Defined for consideration of safety.
(9): Defined for consideration of impact to the evaluation of safety and efficacy.

7 SPECIFIED STUDY DEVICES

As specified study devices, EP-01 (EP-01 main unit and EP-01 accessories), the investigational device, and EX-01 extension wire (Epsilon Medical Inc.) and Neurofax EEG-1200 Electroencephalograph series (Nihon Kohden Corporation), devices used to evaluate the efficacy and safety of EP-01, will be used.

7.1 Investigational Device

Only the principal investigator and sub-investigators of respective medical institutions who receive training on how to use the EP-01 main unit and EP-01 accessories conducted by the coordinating investigator or a physician designated by the coordinating investigator will be allowed to perform the study procedures using the investigational device in this study. For details on the investigational device, refer to the summary on the study devices.

7.1.1 Name or Identifier of the Investigational Device

Name of investigational device : EP-01 endovascular EEG
Identifier of investigational device : EP-01
Classification : IV

EP-01 is comprised of the EP-01 main unit and EP-01 accessories (connector and sheath set, wire winder, inserter, long sheath, wire wing, and dispenser).
Up to 6 EP-01 main units and connectors depending on the case, 2 sheath sets, and up to 2 wire winders, inserters, long sheaths, and wire wings depending on the situation will be used. The EP-01 main unit and EP-01 accessories will be individually packaged.

7.1.2 EP-01 Main Unit

The main unit of the endovascular EEG device is a medical device that measures EEG in a percutaneously transvascular manner, with electrode components attached to the tip of the lead wire. The lead wire is insulated on its surface and has a structure that allows it to connect to the EEG measurement device at the proximal end.
EEG is acquired by the electrode component, and EEG signals are transmitted through the lead wire. The rear end of the lead wire has an exposed metal portion for connection to the connector and is connected to the connector. The connector allows EEG signals acquired by the electrode components to be fed into the EEG measurement device.
Although identification markers are applied on the EP-01 main unit at the proximal side, the identification markers on the tip electrode structure do not differ and are all the same. Six types of identification markers are set to facilitate identification of the EP-01 main unit inserted into the body.

7.1.3 EP-01 Accessories

The EP-01 accessories comprise the connector, sheath set, wire winder, inserter, long sheath, and wire wing (Table 7-1. List of EP-01 Accessories).

Table 7-1. List of EP-01 Accessories

Name of Accessory	Use
Connector	Connects the EP-01 main unit and the EEG measurement device.
Sheath set	Used when inserting the EP-01 main unit into the body, and remains placed while EP-01 is placed.
Wire winder	Secures wires of the EP-01 main unit that are protruding from the patient.
Inserter	Used to facilitate insertion of the tip electrode portion of the EP-01 main unit into the microcatheter.
Long sheath	Used to extend the sheath of a sheath set when the main unit is placed in the body.
Wire wing	Used to secure lead wires while the main unit is placed in the body.
Dispenser	Packaging and protection material to contain and protect the EP-01 main unit during transportation.

7.1.4 Packaging and Labeling of the Investigational Device

The following will be indicated on the labels of the investigational device and EP-01 accessories.

The fact that they are for a clinical trial.
Name and address of the person conducting the study himself/herself
Name of raw materials or identification code
Manufacturer part number or code
Storage method, shelf life

7.2 Procedures for Delivery, Management, and Release of Investigational Device

The study device provider will deliver the investigational device to respective medical institutions after the person conducting the study himself/herself submits the clinical trial plan notification for this study. The study device administrator (or assistant study device manager) of respective medical institutions will manage and release EP-01 in accordance with the Manual on Study Device Management prepared by the person conducting the study himself/herself.

7.2.1 Precautions for Handling of Investigation Device

The storage conditions and shelf life of the investigational device are: the period during which its stability was confirmed in stability tests (3 years after manufacture), and storage with caution for water leakages and avoidance of high temperature, high humidity, and direct sunlight.

7.2.2 Disposal of the Investigation Device

Medical institutions should dispose of used investigational devices in accordance with the procedures of the respective medical institutions. For details, refer to the manual on study device management. Additionally, opened and unused devices should be disposed of in accordance with the regulations of the medical institution, and a disposal record should be prepared. All unused (and unopened) investigational devices should be returned to the study device provider at the end of the study, and a return record should be prepared at that time.

7.3 Other Specified Study Devices

Devices used to evaluate the efficacy and safety of EP-01, EX-01 extension wire (Epsilon Medical Inc.) and an electroencephalograph (Nihon Kohden Corporation), will be used. Safety information associated with EX-01 extension wire and EEG-1200 Electroencephalograph series arising from this study will be appropriately handled in accordance with 13.4 Failures and Adverse Event Case Reports of Other Specified Study Devices (EX-01 extension wire and EEG-1200 Electroencephalograph series).

7.3.1 EX-01 Extension Wire (Epsilon Medical Inc.)

An unapproved device used as needed according to the situation of each case to temporarily extend the overall length of EP-01 when removing the microcatheter from the EP-01 main unit. EX-01 extension wires ("EX-01") are packed individually in single units.

7.3.1.1 Procedures for Delivery, Management, and Release of EX-01

Alike the investigational device, the study device provider will deliver the device after the person conducting the study himself/herself submits the clinical trial plan notification for this study. The study device administrator (or assistant study device manager) of respective medical institutions will manage and release EX-01 in accordance with the Manual on Study Device Management prepared by the person conducting the study himself/herself.

7.3.1.2 Precautions for Handling EX-01

The storage conditions of the device are: storage with caution for water leakages and avoidance of high temperature, high humidity, and direct sunlight.

7.3.1.3 Disposal of EX-1

Medical institutions should dispose of used EX-01 in accordance with the procedures of the respective medical institutions. For details, refer to the manual on study device management. Additionally, opened and unused devices should be disposed of in accordance with the regulations of the medical institution, and a disposal record should be prepared. All unused (and unopened) EX-01 should be returned to the study device provider at the end of the study, and a return record should be prepared at that time.

7.3.2 Neurofax EEG-1200 Electroencephalograph series (Nihon Kohden Corporation)

Used to connect to EP-01 to measure EEG. An electroencephalograph from the EEG-1200 series, regardless of model (except model EEG-1250). For these, devices employed and used in the medical institution will be used, and although they do not need to be managed as part of this study, they should be managed for accuracy in accordance with the regulations of respective medical institutions. The principal investigator or sub-investigators should confirm that the devices are appropriately managed based on the devices' accuracy management record information.

Marketing certification number: 218AHBZX00013000
Manufactured and distributed by: Nihon Kohden Corporation

8 SUBJECT ENROLLMENT

The number of subjects that will receive intervention by EP-01 is set at 37.

8.1 Enrollment Procedure

The principal investigator or sub-investigators will enroll patients after confirming during the screening period that the patients meet the inclusion criteria and do not meet the exclusion criteria. The eligibility criteria will be described and registered on the electronic data capture.

Patients who do not meet the inclusion criteria or meet the exclusion criteria and are not enrolled will not be required to undergo tests of this study after being determined ineligible.

8.2 Definitions of Subjects Assigned in the Study

Subjects who are enrolled in the study and begin EP-01 EEG testing will be considered *EP-01 EEG test-initiated subjects*, and among EP-01 EEG test-initiated subjects, those who complete the testing period will be considered *EP-01 EEG test-completed subjects*, and those who discontinue during the EEG testing period or post-observation period for any reason will be considered *discontinued subjects*. Furthermore, subjects who proceed to partial resection and complete the evaluation 1 year after resection will be considered *epileptic seizure outcome evaluation completed subjects*, and subjects who discontinue before the evaluation 1 year after completing the testing period will be considered *discontinued subjects during epileptic seizure outcome evaluation period*.
Subjects who are not enrolled after consent is obtained will be considered *ineligible patients*, and subjects who do not start EP-01 EEG testing for any reason after enrollment will be considered *dropout subjects*.

9 STUDY METHOD

9.1 Study Design

This is a single-arm, multicenter, open-label study (validation study). An outline of the study is shown in Fig 9-1.

The study is comprised of a screening period of up to 30 days, a testing period of up to 2 weeks, and a post-observation period of 1 week. Subjects who proceed to epileptic focal resection will be evaluated for epileptic seizure outcome 1 year after surgery.

Data up to the end of the post-observation period will be subject to the primary analysis for evaluation of the investigational device. Data on epileptic seizure outcome evaluation 1 year after surgery will be subject to the secondary analysis.

Screening period	Testing Period	Post-observation Period	Epileptic Seizure Outcome Evaluation Period
(30 days to 1 day prior)	(Up to 2 week)	(1 week)	1 year after surgery

Obtaining of consent
Investigational device placement period
End of investigational device evaluation period
End of epileptic seizure outcome evaluation period

Fig 9-1. Outline of the Study

[Rationale]

The screening period (30 days to 1 day prior) was set as the period during which tests specified in the study will be completed. The testing period (maximum 2 weeks) is the duration during which epileptic seizures could occur for evaluation of the investigational device and is the duration during which the safety of the investigational device was confirmed in non-clinical studies. The post-observation period was set as the duration during which adverse events anticipated during the intracranial electrode and EP-01 placement period are anticipated to occur.

9.2 Diagnosis

Subject patients will be those who: are diagnosed with partial-onset epileptic seizure based on clinical and MRI imaging findings and scalp electrode results; have a vascular anatomy that allows EP-01 to be guided to a vessel close to the epileptic focus, such as the cavernous sinus, transverse sinus, superior sagittal sinus, and inferior petrosal sinus; and are determined by physicians to be eligible for EP-01 and intracranial electrode placement. Regarding these tests for decision-making on EP-01 placement, in consideration of the burden on subjects, the decision-making may be based on data obtained up to 90 days prior to obtaining consent.

9.3 Application and Placement Duration of the Investigational Device

The placement of the device in this study will be a single placement, and the maximum duration of placement will be 2 weeks (with an allowance +3 days).

9.3.1 Method of Investigational Device Placement

The tip of the sheath, an EP-01 accessory, will be inserted toward the brain through the right and left jugular veins. The microcatheter will be inserted through the sheath to the target cerebral vein. The EP-01 main unit will be delivered to the target site by inserting through the microcatheter that has already reached the target vessel. The inserter will be used as needed to assist the introduction of the EP-01 main unit into

the microcatheter. After confirming delivery of the EP-01 main unit to the target site, the microcatheter will be withdrawn. When doing so, extension wires will be used as needed. After removal of the microcatheter, EEG acquired at the electrode portion will be fed into the EEG measurement device via the lead wire portion. It will be confirmed that EEG is displayed on the EEG measuring device, and then, the placement of the EP-01 main unit will be complete. When ending the placement, a Tuohy Borst valve or silicone tubing will be used for appropriate securing. After placing a maximum of 6 wires, wires protruding from the subject's neck will be secured, and then, the procedure will be complete.

Although the EP-01 main unit will be placed in vessels close to the epileptic focus, such as cavernous sinus, transverse sinus, superior sagittal sinus, or inferior petrosal sinus, depending on the circumstances of the case, this will be determined by the principal investigator or sub-investigators ("principal investigator and others"). However, it must be confirmed that the EP-01 main unit can be guided to a symmetrical position on both sides of the cerebral vein. Detailed procedures should be performed according to Manual on Study Surgical Procedure.

9.3.2 Method of Intracranial Electrode Placement

As a general rule, intracranial electrodes are placed after EP-01 main unit placement. Placement methods employed in regular clinical practice at respective medical institutions will be followed. The same will apply to the selection of placement site. When placing EP-01 beforehand, as described in 10.2.2 Prohibited or Restricted Concomitant Drugs and Therapies, note that electric scalpels used for surgical procedures during the EP-01 placement period should not be monopolar.

9.3.3 Timing of Investigational Device Placement

As a general rule, the EP-01 main unit should be placed at the time of Wada testing or intracranial electrode placement. If the EP-01 main unit is placed on a separate day from the intracranial electrode placement, the interval between the placement of the intracranial electrode and the EP-01 main unit should be within 3 days. Additionally, as described in 10.2.2 Prohibited or Restricted Concomitant Drugs and Therapies, note that MRI-guided frame placement is not allowed when placing intracranial electrodes after EP 01 placement, because MRI imaging is contraindicated.

9.3.4 During the Investigational Device Placement Period

As a general rule, during the period of the EP-01 main unit placement, heparinized saline solution is continuously perfused through the side port of the Tuohy Borst valve attached to the sheath to prevent thrombosis of the sheath. If the principal investigator and others determine that continuous perfusion with heparinized saline is difficult due to the condition of the subject, flush and lock should be performed at least once a day (heparinized saline is recommended for flushing and locking).

During the period of EP-01 main unit placement, the position of the tip electrode should be confirmed by X-ray testing. If X-ray tests confirm significantly misaligned tip electrodes, the safety of the subject will be given highest priority, and measures such as removal of the EP-01 main unit or follow-up observation should be considered and taken at the discretion of the principal investigator and others. Additionally, the presence or absence of adverse events will be confirmed by CT If adverse events are confirmed, appropriate measures will be taken based on the judgment of the principal investigator and others. Detailed procedures, such as EEG recording method, should be performed according to Manual on Study Surgical Procedure.

9.3.5 Removal of Investigational Device

After confirming the occurrence of an epileptic seizure during placement, the EP-01 main unit is removed at the discretion of the principal investigator and others. If a seizure is not confirmed, the device is placed for up to 2 weeks (with an allowance of +3 days) and then removed. If no seizures occur during placement, or if EEG signals cannot be detected due to some failure, the subject is considered a discontinued subject.

9.3.6 Removal of Intracranial Electrodes

The timing and method of intracranial electrode removal will follow clinical judgment. Intracranial electrodes may be removed at the same time as the investigational device or later.

10 SUBJECT MANAGEMENT

10.1 Prior Treatment Drugs and Therapies

Drugs and therapies of prior treatment are discontinued or reduced as is usually done in regular clinical practice at the physician's discretion on an as-needed basis in line with the intracranial electrode placement. The history of prior treatment for the primary disease before enrollment (including surgical history) is recorded in case report forms as a prior treatment history.

10.2 Concomitant Drugs and Therapies

Treatment that is deemed necessary for the benefit of the subject may be performed at the discretion of the principal investigator and others.

Drugs (prescription drugs) and therapies used between enrollment up to the end of the post-observation period are recorded in case report forms as concomitant drugs and therapies.
Concomitant drugs and therapies used for intracranial electrode placement (including additional placement) and removal are excluded from information collection. Additionally, if epileptic focal resections are performed before the end of the post-observation period, concomitant drugs and therapies used for these procedures will also be excluded from information collection. However, antithrombotic therapy will be as described in 10.2.1.

10.2.1 Antithrombotic Therapy

Antithrombotic therapies are recorded in case report forms as antithrombotic therapies between 30 days prior to obtaining consent until the end of the post-observation period.

10.2.2 Prohibited or Restricted Concomitant Drugs and Therapies

MRI imaging is contraindicated during EP-01 placement. Additionally, in order to minimize the effect of electric current, electric scalpels used for surgical procedures during the EP-01 placement period may not be monopolar ones using patient plates. During the course of the study period, there are no rules other than the above on prohibited or restricted concomitant drugs or therapies that have been added to restrictions on intracranial electrode placement practiced in routine clinical practice.

10.2.3 Concomitant Drugs and Therapies Requiring Caution

During the course of the study period, there are no rules on concomitant drugs or therapies requiring caution.

11 TEST/OBSERVATION PARAMETERS AND SCHEDULE

Rules on the timing in this study are as follows. The following observations and tests will be performed on the provided day of visit. The schedule for respective tests is set with the day of device placement being the starting point (Day 0).

11.1 Observation Parameters

11.1.1 Examination of Subject Background

After obtaining consent, investigators will confirm that patients meet the inclusion criteria and do not meet the exclusion criteria during 30 days to 1 day prior to placement of the investigational device, and then the following items will be examined at the time of enrollment. Subject identifier codes and subject background information will be recorded in case report forms.
Observation parameters: Sex, age, blood pressure, pulse, current medical history (epilepsy classification, seizure type classification, duration, years, etiology), complications, other medical history, prior treatment history, smoking history, alcohol consumption history
Complications: Diseases that are complicating factors at the time consent is obtained.
Other medical history: Diseases that developed at the time consent was obtained or earlier; information up to 90 days prior to the day consent was obtained will be examined. However, the timing will be disregarded for any medical history that the principal investigator or sub-investigators deem important, including perinatal abnormality, febrile seizure, head injury, psychiatric disease, and malignant tumor.
Prior treatment history: History of prior treatment (including surgical procedures) for the primary disease prior to enrollment.

11.1.2 Height and Weight

Weight and height will be measured and recorded in medical records and case report forms.
Timing: After obtaining consent, measurements will be taken 30 days to 1 day prior to investigational device placement; however, if they have been performed within 90 days prior to obtaining consent, no measurements are required, and data prior to obtaining consent will be used.
Measurement parameters: Height and weight.

11.1.3 Cervical Ultrasonography

Cervical ultrasonographies will be performed, and diagnostic findings will be recorded in medical records and case report forms.
Timing: After obtaining consent, tests will be performed 30 days to 1 day prior to investigational device placement; however, if they have been performed within 90 days prior to obtaining consent, no testing is required, and data prior to obtaining consent will be used.
Observation parameters: Presence of clinically problematic occlusion or thrombus in the common carotid artery or internal jugular vein.

11.1.4 Blood Test

Blood tests will be performed, and the results will be recorded in medical records and case report forms.
Timing: After obtaining consent, tests will be performed 30 days to 1 day prior to investigational device placement; however, if they have been performed within 90 days prior to obtaining consent, no testing is required, and data prior to obtaining consent will be used.
Test parameters: platelet count, APTT, PT-INR

11.1.5 Non-invasive Findings

11.1.5.1 Clinical Findings of Epileptic Seizures

Clinical findings regarding epileptic seizures are recorded in case report forms. Depending on the subject's condition, only those necessary for lateralization diagnosis of epileptic foci will be examined at the discretion of the principal investigator and others.
Timing: After obtaining consent, examinations will be performed 30 days to 1 day prior to investigational device placement; however, if they have been performed within 1 year prior to obtaining consent, no re-examination is required, and data prior to obtaining consent will be used.
Observation parameters: Contents of medical questionnaires specified in guidelines on epilepsy, neuropsychological testing.

Contents of medical questionnaires in epilepsy guidelines: Frequency of seizures; condition and triggers of seizures; symptoms before and during seizures; persistence of symptoms; symptoms following seizures; presence of trauma, tongue biting, urinary incontinence; and postictal headache and muscle pain. For subjects who have experienced multiple seizures: age of onset; changes and transitions in seizure and seizure type; last seizure; relation between seizure and arousal/sleep; postictal behavior; and details of condition.
Neuropsychological testing: Presence of functional impairment in the left and right frontal, temporal, occipital, and parietal lobes.

11.1.5.2 MRI Imaging findings

MRI and other imaging data (including CT, fluorodeoxyglucose-positron emission tomography (FDG-PET), ^{123}I-iomazenil single photon emission computed tomography (IMZ-SPECT), magnetoencephalography (MEG), and others, if they are performed in regular medical care) for lateralization diagnosis of epileptic foci will be acquired. The acquired data should be submitted to the Central Evaluation Committee in accordance with the Manual on Central Evaluation Committee.
Timing: As a general rule, information on tests performed for lateralization diagnosis of epileptic foci within 1 year prior to obtaining consent is collected. Re-imaging for the purpose of the study is not required even if imaging has not been performed within 1 year. If re-imaging is not performed, the latest imaging findings are submitted to the Central Evaluation Committee.

11.1.5.3 Scalp Electrode Test

Scalp electrode test data for lateralization diagnosis of epileptic foci will be acquired. The acquired data should be submitted to the Central Evaluation Committee in accordance with the manual on the Central Evaluation Committee.
Timing: As a general rule, information on tests performed for lateralization diagnosis of epileptic foci within 1 year prior to obtaining consent will be collected. Re-testing for the purpose of the study is not required even if tests have not been performed within 1 year. If re-testing is not performed, the latest EEG findings are submitted to the Central Evaluation Committee.

11.1.6 MRI Test

Simple 3D-T1WI and either MRV or contrast-enhanced 3D-T1WI are performed to confirm that the subject has vascular anatomy that allows the EP-01 main unit to be guided to a vessel close to the epileptic focus, such as cavernous sinus, transverse sinus, superior sagittal sinus, or inferior petrosal sinus, and the diagnostic findings are recorded in medical records and case report forms.
Timing: If they have been performed within 90 days prior to obtaining consent, no re-testing is required, and data prior to obtaining consent will be used.

11.1.7 Confirmation of Location of Microcatheter Placement

The location where the microcatheter will be placed during the EP-01 placement procedure is confirmed.
Timing: During the EP-01 placement procedure.
Observation parameters: Name of vessel in which the microcatheter will be placed.

11.1.8 Confirmation of Location of EP-01 Placement

The location where the tip electrode of the EP-01 main unit will be placed will be confirmed by an X-ray test.
Timing: Immediately after placement, 1 day after placement, 7 days after placement, and immediately before removal. (However, if removal is performed within 7 days, testing 7 days after placement is omitted.)
Observation parameters: Name of vessel in which EP-01 is placed at each time period.

11.1.9 CT Scan

The presence of any adverse events at the location of EP-01 main unit placement is confirmed by CT scan. 3D data of slice thickness of 1 mm or less and 3–5mm is obtained. The acquired data should be submitted to the Safety Evaluation Committee in accordance with the Manual on Safety Evaluation Committee. Only if it is performed within 90 days prior to obtaining consent in regular medical care, it is provided to the Safety Evaluation Committee. If it is not performed, re-imaging is not required.
Timing: Immediately after placement, 1 day after placement, 7 days after placement, and immediately before removal. (However, if removal is performed within 7 days, testing 7 days after placement is omitted.)

11.1.10 EEG Test

EEG with EP-01 and intracranial electrodes are measured under video surveillance. Detailed procedures, such as EEG recording method, should be performed according to Manual on Study Surgical Procedure. The acquired data should be submitted to the Central Evaluation Committee in accordance with the Manual on Central Evaluation Committee.
Timing: From device placement to device removal (including the period when only EP-01 or both EP-01 and intracranial electrodes are placed).
Observation parameters: Information on EP-01 and intracranial electrode placement, lateralization diagnosis results based on non-invasive findings combined with EP-01-based EEG findings, and lateralization diagnostic results based on non-invasive findings combined with intracranial electrode-based EEG findings.

EEG data provided to the Central Evaluation Committee:

- EEG data when eyes are opened and closed

  EEG data when eyes are opened and closed will be acquired at the time point determined by the principal investigator and others.

- EEG data during epileptic seizures

  <Selection criteria for subject epileptic seizure EEG>
  - Seizures with clinical symptoms
  - Seizures with both EP-01 EEG and intracranial electrode EEG clearly recorded
  - Seizures that are not part of status epilepticus
  - Among seizures that meet the above, the first seizure used for diagnosis

11.1.11 Observation of Adverse Events

Adverse events are defined as various unfavorable signs, symptoms, or diseases (including abnormal fluctuations in laboratory values) observed during the testing period and post-observation period after starting the use of EP-01 (main unit and accessories) or other specified study devices (EX-01 extension wire, Neurofax EEG-1200 Electroencephalograph series), regardless of causal relationship to the specified study device. If an adverse event is observed, the principal investigator and others should take appropriate measures. Additionally, as a general rule, follow-ups will be conducted until the adverse event normalizes or returns to a level where it is no longer considered an adverse event, regardless of causal relationship to the investigational device. If the adverse event is an organic disorder and irreversible, follow-ups will be conducted until the symptoms are stabilized or fixed. Symptoms within the range expected of the primary disease will not be treated as adverse events. If an epileptic focal resection is performed during the testing period and post-observation period, symptoms attributable to the procedure will not be treated as adverse events. For all adverse events that occur during the testing period and post-observation period after the use of the EP-01 (main unit and accessories), the following items will be recorded in medical records and case report forms.

- Name of adverse event.
  Date of occurrence
- Date of outcome
- Outcome (recovered, improved, not recovered, death, lost to follow-up, unknown).
  Treatment (yes, no; if no, the details of the treatment)
- Severity (mild, condition allowing continuance of study participation without treatment; moderate, condition allowing continuance of study though some treatment is required; severe, condition for which study participation will be or should be discontinued)
- Seriousness (serious, not serious)
- Relationship (related, not related, unknown) to specified study devices (including medical acts for placement and removal) or intracranial electrodes

11.1.12 Observation of Failures

Failure is defined as any of the broad range of poor conditions of the specified study devices including malfunction, adverse reactions caused to the human body, damage, and inability to operate of specified study devices, regardless of whether it is at the stage of manufacture, delivery, storage, or use.
For all failures that occur during the testing period, the following items will be recorded in medical records and case report forms.

- Name of failure
- Possible causes of the specified study device failure
  (transportation, storage, procedure, primary disease, concomitant drugs / adjunctive therapy, other)
- Status of specified study device failure
- Relationship to EP-01 main unit, EP-01 accessories (connector and sheath set, wire winder, inserter, long sheath, wire wing, dispenser), Neurofax EEG-1200 Electroencephalograph series, and EX-01 extension wires

11.1.13 Concomitant Drugs and Therapies

As described in 10.2 Concomitant Drugs and Therapies, the drugs (prescription drugs) and therapies used from enrollment to the end of the post-observation period should be recorded in case report forms as concomitant drugs and therapies. Antithrombotic therapies should be recorded in case report forms as antithrombotic therapy from 30 days prior to obtaining consent until the end of the post-observation period. Concomitant drugs and therapies used for intracranial electrode placement (including additional placement) and removal are excluded from information collection. Additionally, if epileptic focal resections are performed before the end of the post-observation period, concomitant drugs and therapies used for these procedures will also be excluded from information collection.

However, antithrombotic therapy will be as described in 10.2.1.

11.1.14 ILAE Classification

The postoperative epileptic seizure outcome will be evaluated using the ILAE classification[14)] in accordance with the Manual on Evaluation of Postoperative Epileptic Seizure Outcome, and the results will be recorded in medical records and evaluation sheets. The evaluation results will be sent to the study coordination secretariat.
Timing (acceptable range): 365 days (±60 days) after the date of surgical treatment.
Observation parameters: Date of surgical procedure, site of surgical removal, date of evaluation, ILAE classification results.

11.1.15 Evaluation by the Central Evaluation Committee

The coordinating investigator will establish a Central Evaluation Committee for the purpose of conducting a third-party evaluation of efficacy. The Central Evaluation Committee will evaluate the lateralization diagnosis of epileptic foci based on data obtained at respective medical institutions in accordance with the Manual on Central Evaluation Committee.

Table 11-1. Evaluation Items of the Central Evaluation Committee

Purpose and timing of efficiency evaluation	The purpose is to use third-party evaluation for lateralization diagnosis of the epileptic foci in order to avoid suspicion of biased evaluation. The evaluation will be performed after the principal investigator and others finish determining lateralization diagnosis results on epileptic foci.<br>Timing: Evaluations will be performed each time after case data is sent from respective medical institutions to the central evaluation physician via the Central Evaluation Committee secretariat.

In the above operations, if the Central Evaluation Committee determines it is necessary to hear the opinions of other experts, it will contact the coordinating investigator and then request other experts to participate as observers and hear their opinions.

11.1.16 Evaluation by the Safety Evaluation Committee
The coordinating investigator will establish a Safety Evaluation Committee for the purpose of conducting a third-party safety evaluation. The Safety Evaluation Committee will evaluate safety based on the data obtained at respective medical institutions in accordance with the Manual on Safety Evaluation Committee.

Table 11-2. Evaluation Items of the Safety Evaluation Committee

Purpose and timing of safety evaluation	The purpose is to evaluate safety and evaluate the appropriateness of continuing the study.<br>(1) Periodic evaluation<br>The presence, severity, relationship to devices of adverse events* determined based on image tests taken at respective medical institutions will be evaluated.<br>*Adverse events determined by image testing: hemorrhage, midline shift, cerebral spinal fluid leak.<br>The safety evaluation will be conducted at the timing of periodic meetings specified in the protocol after CT image data is submitted by the respective medical institutions to the study coordinating office.<br>・ First periodic meeting: coordinated when data of 15 to 20 cases (note: modified according to the number of cases) is submitted by respective medical institutions to the Safety Evaluation Committee secretariat.<br>・ Second periodic meeting: when data of all cases is submitted by respective medical institutions to the Safety Evaluation Committee secretariat.<br><br>(2) Non-periodic evaluation<br>Meetings will be held as non-periodic meetings if the coordinating investigator deems it necessary based on the occurrence of adverse events, such serious adverse events caused by the investigational device, and safety information provided by the study device provider.<br>Recommendations on the continuance of the study and changes to the protocol will be provided if the coordinating investigator deems it necessary based on serious adverse events attributable to the investigational device that are reported by respective medical institutions and on the following.<br>• When new important safety information concerning the continuation of the study overall is obtained<br>• When an adverse event (or adverse reaction) or failure that substantially exceeds initial projections occur<br>• When efficacy is significantly more or less effective than initially projected<br>• When new significant information regarding safety or efficacy is obtained<br><br>(3) Other<br>Held when the coordinating investigator deems it otherwise necessary.

In the above operations, if the Safety Evaluation Committee determines it is necessary to hear the opinions of other experts, it will contact the coordinating investigator and then request other experts to participate as observers and hear their opinions.

11.2 Test/Observation Parameters and Schedule
The test/observation parameters and schedule are shown in Table 11-3.Test/Observation Parameters and Schedule.

Table 11-3. Test/Observation Parameters and Schedule

Item		Screening Period	Investigational Device Evaluation Period								Epileptic Seizure Outcome Evaluation
			Testing Period						Post-observation Period		
Timepoint		Screening		Investigational device placement[10]	1 day after investigational device placement	Intracranial electrode placement	1 week after investigational device placement	Investigational device removal[11]/ discontinuation[15]	1 day after investigational device removal	1 week investigational device removal / discontinuation[15]	1 year after surgical treatment[12]
Acceptable Range		30 days to 1 day prior to investigational device placement				Within ±3 days from investigational device placement		Within 14 days after investigational device placement[13]	+3 days	+3 days	±60 days
Obtaining of consent		●									
Enrollment			●								
Confirmation of eligibility		●									
Examination of Subject Background[1]		●									
Height/weight[2]		●									
Cervical ultrasonography[3]		●									
Blood test[4]		●									
Non-invasive findings[5]	Clinical findings on epileptic seizures	●									
	Image findings	●									
	Scalp electrode	●									
Image test	MRI test[6]	●									
	X-ray test[7]			●	●		●	●			
	CT[8]	○[14]		●	●		●	●	●	●	
EEG test[16]				←	—	—	—	→			
Adverse events				←	—	—	—	—	—	→	
Failures				←	—	—	—	→			
Primary disease treatment / antithrombotic therapy[9]		←	—	—	—	—	—	—	—	→	
Concomitant medication				←	—	—	—	—	—	→	
Epileptic seizure evaluation (ILAE classification)											●

●: Data or observation items required in this study ○: Performance not required for the purpose of this study

1) Information on age, sex, blood pressure, pulse, current medical history, complications, other medical history, smoking history, and alcohol consumption history is collected at the time consent is obtained.

2),3) If they have been performed within 90 days prior to obtaining consent, no re-testing is required, and data prior to obtaining consent is collected.

4) If they have been performed within 90 days prior to obtaining consent, no re-measurement is required, and data prior to obtaining consent will be used. Platelet count, APTT, and PT-INR tests are performed.

5) Depending on the subject's condition, only those necessary for lateralization diagnosis of the epileptic focus are investigated at the discretion of the principal investigator and others.

6) Simple 3D T1-weighted imaging (3D-T1WI) and either magnetic resonance venography (MRV) or contrast-enhanced 3D-T1WI are performed. If they have been performed within 90 days prior to obtaining consent, no re-measurement is required, and data prior to obtaining consent will be used.

7) X-ray tests (frontal and lateral) are performed. The tests are performed immediately after placement of the investigational device, the day after placement of the investigational device, 1 week after placement of the investigational device (unnecessary if seizures occur within 1 week from placement of the investigational device), and immediately before removal of the investigational device.

8) Imaging methods immediately after EP-01 placement are not limited to multislice computed tomography (CT) and also include cone-beam computed tomography (CBCT). The CT tests are performed immediately after placement of the investigational device, the day after placement of the investigational device, 1 week after placement of the investigational device (unnecessary if seizures occur within 1 week from placement of the investigational device), and immediately before removal of the investigational device. 3D data of slice thicknesses of 1 mm or less and 3–5mm is submitted to the Safety Evaluation Committee.

9) Information on treatment (including surgical procedures) and antithrombotic therapy for the primary disease is collected from before consent is obtained and until the end of the post-observation period.

10) As a general rule, the investigational device is placed during the same procedure as the Wada test or intracranial electrode placement.

11) The investigational device is placed until the occurrence of an epileptic seizure or for a maximum of 14 days (+3 days). The investigational device may be removed on the same day as the intracranial electrode removal or surgical treatment. If a shift in the tip position of the investigational device is suspected, an x-ray test is promptly performed to determine whether or not to remove.

12) Performed only on subjects who underwent epileptic resection.

13) If it is difficult to remove within up to 2 weeks due to the system of respective medical institutions, an allowance of +3 days may be set.

14) Only if it is performed within 90 days prior to the placement of the investigational device in regular medical care, it is provided to the Safety Evaluation Committee. If it is not performed, re-imaging is not required.

15) In the case of discontinuation during the testing period, depending on the subject's situation, the investigational device is removed and the subject is moved to the post-observation period after performing, as much as possible, tests specified at investigational device removal.
In the case of discontinuation during the post-observation period, depending on the subject's situation, the subject is withdrawn after performing, as much as possible, tests specified at 1 week after investigational device removal.

16) During the period when only EP-01 or both EP-01 and intracranial electrodes are placed, EEG with EP-01 and the intracranial electrodes are measured under video surveillance. EEG data when the eyes are opened and closed (the timing of acquiring EEG data when the eyes are opened and closed is determined by the principal investigator and others) and EEG data during epileptic seizures are submitted to the Central Evaluation Committee.

12 ENDPOINTS

12.1 Primary Endpoint

- Diagnostic performance of EP-01 for lateralization diagnosis of epileptic focus: third-party evaluation by the Central Evaluation Committee.

Diagnostic results on epileptic focus lateralization ("lateralization"), based on clinical findings, imaging findings obtained within 1 year prior to the date of obtaining consent, and scalp electrode-based EEG findings ("non-invasive findings") combined with EP-01-based EEG findings, compared with the diagnostic results on the lateralization based on non-invasive findings combined with intracranial electrode EEG findings, and the percentage agreement.

[Rationale]

In actual clinical practice, lateralization is diagnosed based on the three non-invasive findings (clinical, imaging, and scalp electrode findings) combined with intracranial electrode results to decide the site of focal resection for epilepsy. To verify whether EP-01 can substitute intracranial electrodes in actual clinical practice, third-party evaluation of the correctness of the following two results was employed as the evaluation method for the primary endpoint.

1. Lateralization diagnosis results based on the above 3 non-invasive findings and EP-01 EEG findings
2. Lateralization diagnosis results based on the above 3 non-invasive findings and intracranial electrode EEG findings

Additionally, to avoid bias, the lateralization diagnoses in 1 and 2 above will be performed by separate evaluators, and then similarity in the diagnosis results will be confirmed.

Since postoperative Engel Class I/II after focal resection for mesial temporal lobe epilepsy is seen in 90% or more cases[8)], it can be inferred that seizure resolution after intracranial electrode-based lateralization diagnosis followed by resection with surgical procedures has been confirmed in 90% or more cases. Accordingly, the diagnostic performance of intracranial electrodes for lateralization diagnosis is considered to be at least more than 90%. Consequently, it was deemed reasonable to use the intracranial electrode lateralization diagnosis results as a control for the primary endpoint of this study.

12.2 Secondary Endpoints

12.2.1 Diagnostic Performance of EP-01 Alone for Lateralization Diagnosis of Epileptic Focus: Third-party Evaluation by the Central Evaluation Committee

Lateralization diagnosis results based on EP-01 EEG findings alone compared with the lateralization diagnosis results based on intracranial electrode EEG findings alone, and the percentage agreement.

[Rationale]

This was set to also evaluate the correctness of the intracranial electrode and EP-01 results alone without the 3 non-invasive findings, among the primary endpoints. To avoid bias, a third-party will perform evaluations.

12.2.2 EEG Identification of EP-01 Waveforms

12.2.2.1 Comparative Evaluation with EEG Findings When Eyes are Opened and Closed: Third-party Evaluation by the Central Evaluation Committee

It will be confirmed if waveforms detected by EP-01 are due to EEG.

[Rationale]

To confirm whether waveforms detected by EP-01 are due to EEG without any invasiveness for subjects, EEG findings when the eyes are opened and closed will be confirmed.

12.2.2.2 Comparative Evaluation of EEG Measurement Performance When EP-01 Alone and EP-01 Combined with Intracranial Electrodes are Inserted: Evaluation by the Principal Investigator and Others

If there is a period of time when only EP-01 is placed, a comparative evaluation will be performed on EEG measurement performance when EP-01 alone and EP-01 combined with intracranial electrodes are inserted to determine if there is any mutual effect.

[Rationale]
Simultaneous placement of intracranial electrodes and EP-01 may affect EEG measurement performance. Therefore, for subjects in whom EP-01 alone have been placed for a period, a comparative evaluation will be performed on EEG measurement performance when EP-01 alone and EP-01 combined with intracranial electrodes are inserted to confirm the presence of changes in EP-01 EEG performance.

12.2.3 Diagnostic Performance of EP-01 for Lateralization Diagnosis of Epileptic Focus (Percentage Agreement with Intracranial Electrode-based Diagnosis Results): Third-party Evaluation by the Central Evaluation Committee

- Lateralization diagnosis results based on EP-01 EEG findings and non-invasive findings compared with the lateralization diagnosis results based on bilaterally-placed intracranial electrode EEG findings and non-invasive findings, and the percentage agreement.
- Lateralization diagnosis results based on EP-01 EEG findings alone compared with the lateralization diagnosis results based on bilaterally-placed intracranial electrode EEG findings alone, and the percentage agreement.

[Rationale]
This was set to also evaluate the correctness of the EP-01 lateralization diagnosis results compared with the bilaterally-placed intracranial electrode lateralization diagnosis, among the primary endpoint and secondary endpoint 12.2.1.

12.2.4 Diagnostic Performance of EP-01 for Lateralization Diagnosis of Epileptic Focus: Evaluation by the Principal Investigator and Others

Lateralization diagnosis results based non-invasive findings combined with EP-01 EEG findings compared with lateralization diagnosis results based on non-invasive findings combined with intracranial electrode EEG findings, and the percentage agreement.

[Rationale]
Refer to rationale for the primary endpoint. The lateralization diagnosis by the physician in charge was set because this is done in actual clinical practice.

12.2.5 Seizure Resolution Rate 1 year After Focal Resection

In accordance with regular medical care, evaluation of epileptic seizure outcome 1 year after focal resection based on focal lateralization diagnosis using non-invasive findings and intracranial electrode EEG findings. The distribution rates will be evaluated using the ILAE classification.

[Rationale]
One way to determine the effectiveness of epileptic focal resection is to evaluate the outcome of epileptic seizures. Epileptic focal resections will be performed based on intracranial electrode diagnosis results; this was set to evaluate the outcome. Additionally, the study will employ indicators publicly announced by International League Against Epilepsy (ILAE) which are commonly used internationally.

12.2.6 Success Rate of EP-01 Placement (%)

The number of placed EP-01 main units immediately after placement / number of placed microcatheters multiplied by 100 will be calculated and evaluated as success rate.

Any migration of the EP-01 main unit from the intended placement vessel will be assessed immediately prior to removal.

[Rationale]
This was set to confirm that EP-01 can be placed in the planned vessel to perform EEG tests without problems.
It was also set to confirm how far the location of the tip electrodes migrate from the intended placement vessel during the EP-01 placement period.

12.2.7 Safety Evaluation

- Safety evaluation: third-party evaluation by the Safety Evaluation Committee

  The presence, severity, relationship to devices of adverse events* determined based on image tests taken at respective medical institutions will be evaluated.

  *Adverse events determined by imaging testing: hemorrhage, midline shift, cerebral spinal fluid leak

  Furthermore, the incidence rate of adverse events attributable to the investigational device and adverse events attributable to the intracranial electrodes (including medical acts for placement and removal of both devices) will be compared. [Incidence period: from the time EP-01 (main unit and accessories) is used up to the end of the post-observation period evaluation.]

- Occurrence of adverse events: evaluation by principal investigator and others

  [Incidence period: From the start of the use of the investigational device to the end of the post-observation period. Relation to all investigational devices (EP-01 main unit and EP-01 accessories), relation to medical acts for EP-01 placement and removal, relation to other specified study devices, severity, seriousness, treatment, and outcome].

[Rationale]

In addition to collecting information on the occurrence of common adverse events, the incidence rate of adverse events attributable to the investigational device and adverse events attributable to the intracranial electrodes will be compared to evaluate the safety and invasiveness of the investigational device.

12.2.8 Other Evaluations

- Failure of the investigational device EP-01 (failure of EP-01 main unit and EP-01 accessories).
- Failure of other specified study devices (EX-01 extension wires, electroencephalograph)

[Rationale]

The above items will be evaluated to evaluate the performance of devices used in this study, including EP-01.

13 HANDLING OF ADVERSE EVENTS AND FAILURES

13.1 Response to Subjects at Occurrence of Adverse Events

When adverse events are observed, the principal investigator and others will immediately take appropriate measures and record it in medical records and case report forms without any discrepancy. If EP-01 placement is discontinued or if treatment is required for the adverse event, subjects should be informed as such.

13.2 Reporting of Serious Adverse Events and Failures

If the principal investigator and others learn of the occurrence of adverse events classified as "serious" in seriousness described in 11.1.11 Observation of Adverse Events and, among failures provided in 11.1.12 Observation of Failures, failures that may cause death and other serious adverse events (including cases where serious adverse events actually occur), they will take necessary measures, such as explaining to the subject, in accordance with the Manual on Handling of Safety Information.

[Handling of information on adverse events determined to be serious]
If adverse events occur in subjects during the study and the principal investigator and others determine that the event is serious, information on the adverse events will be handled in accordance with the following procedures.

(1) Report from the principal investigator to the head of the medical institution, coordinating investigator, study device provider, and manufacturer of specified study devices

The principal investigator (the person conducting the study himself/herself) will promptly report information on the adverse event (as a general rule, within 24 hours after obtaining the information) to the head of the medical institution and the coordinating investigator, as well as report to the study device provider, regardless of the causal relationship.

If the principal investigator observes the occurrence of adverse events whose causal relationship to other specified study devices (EX-01 extension wire or EEG-1200 Electroencephalograph series) cannot be ruled out, the principal investigator will also report the event to the manufacturer of the specified study devices.

(2) Report to the Minister of Health, Labour and Welfare

If the coordinating investigator (the person conducting the study himself/herself) determines, upon agreement with the principal investigator of respective medical institutions, that the adverse events should be reported to the Minister of Health, Labour and Welfare (discussing with the principal investigator of respective medical institutions as needed), the coordinating investigator (the person conducting the study himself/herself) will prepare "Form 1" and "Adverse Event Sorting Form" and report to the Pharmaceuticals and Medical Devices Agency ("PMDA").

(3) Handling of additional information obtained

If the principal investigator (the person conducting the study himself/herself) obtains additional information on adverse events, the principal investigator will make an additional report to the head of the medical institution and the coordinating investigator as soon as possible, as well as report to the study device provider. The handling of such additional information will be subject to the procedures described in 1 to 2 above, and reports will be made to PMDA as needed.

13.2.1 Definition of Serious Adverse Events and Failures That May Cause Serious Health Hazards

Serious adverse events are defined as any of the following among adverse events. Additionally, failures that may cause serious health hazards are failures that the principal investigator and others deem to pose risks of causing such serious health hazards even if no death or disability described below has occurred in reality.

(1) Death
(2) Life-threatening
(3) Hospitalization or prolongation of hospitalization
(4) Disability
(5) Risk of disability
(6) Serious according to the above
(7) Congenital disease or anomaly in later generations

Among the hospitalizations in 3, hospitalizations for the conduction of this study or the following hospitalizations are not considered as serious adverse events.

- Hospitalization or prolonged hospitalization for therapies that were planned prior to study participation (e.g., surgeries or tests that were planned prior to study participation)
- Hospitalization or prolonged hospitalization to perform tests
- Hospitalization or prolonged hospitalization for surgeries or other treatments not due to the exacerbation of complications or associated symptoms but due to changes in treatment plans for the complications or associated symptoms
- Hospitalization or prolonged hospitalization for follow-ups despite cure or improvement of conditions
- Hospitalizations for adverse events that would normally be treated by outpatient medical care
- Hospitalization or prolonged hospitalization for social reasons (e.g., recuperation of caregiver, absence of family)

13.2.2 Serious Adverse Events and Failures Subject to Reporting

(1) All serious adverse events occurring from the start of use of the investigational device to the end of the post-investigational period for which a causal relationship to the investigational device cannot be ruled out
(2) Serious adverse events suspected to be related to specified study devices after the termination (discontinuation) of the study
(3) Failures of specified study devices that result in or may result in events described in 13.2.1
(4) Infections suspected of being related to specified study devices that result in or may result in events described in 13.2.1.

13.2.3 Procedures for Serious Adverse Event and Failure Reporting

13.2.3.1 Investigational Device (EP-01)

The principal investigator will report serious adverse events and failures for which a causal relationship to the investigational device EP-01 (main unit and accessories) cannot be ruled out in accordance with Reporting of Failures of Machinery and Equipment in Clinical Trials (No. 0831-9; Pharmaceutical and Environmental Health Bureau, Ministry of Health, Labour and Welfare; August 31, 2020) and Considerations in Reporting Failures of Machinery and Equipment in Clinical Trials (No. 0831-10; Pharmaceutical and Environmental Health Bureau, Ministry of Health, Labour and Welfare; August 31, 2020). Reports should be in the form of first report (emergency report) and second report (detailed report), use the GCP unified form (Form 14), and reported between the date the event was learned of up to the date indicated below. For details, follow the Manual on Handling Safety Information.

(1) Unknown; adverse events that result in or may lead to death: within 7 days
(2) Unknown; other serious adverse events: within 15 days
(3) Known; adverse events that result in or may lead to death: within 15 days
(4) Failures that may cause 1 or 2 above: within 30 days

13.2.4 Reporting by the Coordinating Investigator to the Study Device Provider

The coordinating investigator will report serious adverse events and failures to the study device provider in accordance with the Manual on Handling of Safety Information.

13.3 Reporting of Other Adverse Events

Other adverse events will be described in case report forms in accordance with procedures described in 11.1.11 Observation of Adverse Events, 11 Test/Observation Parameters and Schedule of this protocol.

13.4 Case Reports on Failures and Adverse Events of Other Specified Study Devices (EX-01 extension wire and EEG-1200 Electroencephalograph series)

With regard to other specified study devices used in this study (EX-01 extension wire and EEG-1200 Electroencephalograph series), among the failures and adverse events reported in this study, the following should be reported to PMDA as case reports of failures and adverse events of clinical trial devices: failures and adverse events that result in or may lead to death and for which a causal relationship to EX-01 extension wire or EEG-1200 Electroencephalograph series cannot be ruled out, and other serious failures and adverse events for which a causal relationship to EX-01 extension wire and EEG-1200 Electroencephalograph series cannot be ruled out and that could not be predicted. The predictability will be determined based on the summary on the study devices for EX-01 extension wire and the package insert for EEG-1200 Electroencephalograph series; failures and adverse events not described in these will be considered unpredictable failures and adverse events. Additionally, if serious adverse events or failures for which a causal relationship to EX-01 extension wire and EEG-1200 Electroencephalograph series cannot be ruled out occur, the principal investigator will provide any information learned to the head of medical institution and the coordinating investigator.

14 HANDLING OF DEVIATIONS FROM THE PROTOCOL

(1) The principal investigator and sub-investigators must not deviate from or change the protocol prior to obtaining approval from the head of medical institution based on prior review by the Investigational Review Board.

(2) The principal investigator and sub-investigators may deviate from or change the protocol before obtaining prior approval from the Investigational Review Board for inevitable reasons such as the avoidance of emergencies. In such cases, the principal investigator or sub-investigators should promptly submit to the Investigational Review Board the details and reasons of the deviation or change and, if the protocol needs to be revised, a draft of the revision, and obtain approval of the Investigational Review Board and the head of the medical institution.

(3) If there are any deviations from the protocol, the principal investigator or sub-investigators must record all deviations from the protocol along with the reasons for the deviations.

(4) If the principal investigator or sub-investigators learn that the study does not conform (limited to significant degree of non-conformity) with Ministerial Ordinance on Standards for Conducting Clinical Studies on Medical Devices (No. 36; Ministerial Ordinance by the Ministry of Health, Labour and Welfare; March 23, 2005), the principal investigator or sub-investigators must promptly report to the head of the medical institution, take necessary measures, and then cooperate with the head of the medical institution in reporting and publicly announcing the status and results of such measures to the Minister of Health, Labour and Welfare.

15 DISCONTINUATION CRITERIA AND PROCEDURE

15.1 Termination of the Study

At the end of the study, the principal investigator will promptly submit a study termination report to the head of the medical institution. Additionally, the principal investigator will publicly announce results in accordance with 22 Arrangements on Public Disclosure.

15.2 Discontinuation or Suspension of the Study

If the following applies, the principal investigator will consider whether or not to continue the study.

(1) When significant information regarding the quality, safety, or efficacy of the investigational device is obtained
(2) When facts or information that undermine the appropriateness or reliability of the conduct of the study are obtained
(3) When it is difficult to recruit subjects and it is deemed difficult to achieve the planned number of subjects
(4) When the objective of the study is achieved or when it is determined that the expected results cannot be achieved before reaching the planned number of subjects or the planned period
(5) When the Investigational Review Board instructs change to the study plan and it is determined to be difficult to accept such instructions

Additionally, if 1 or 2 above applies, the principal investigator will promptly report to the head of the medical institution and modify the protocol as necessary. If the Investigational Review Board recommends or instructs discontinuation, the study will be discontinued. When the discontinuation or suspension of the study is decided, the event and its reasons will be promptly reported in written form to the head of the medical institution.

15.3 Policy on Measures for Subjects After Study Discontinuation and Termination

The principal investigator and sub-investigators will use their effort to ensure that subjects can continue receiving the best treatment after the termination of the study.

15.4 Study Dropout and Discontinuation Criteria for Individual Subjects

In any of the following cases, subjects will discontinue the study and receive appropriate measures/treatment. If subjects discontinue the study after placement of the investigational device, testing planned at the time of investigational device removal and during the post-observation period should be performed as much as possible. If an individual subject discontinues the study, an evaluation will be performed in a manner that takes the subject's safety into account at the time of discontinuation and documented in the case report form.
Additionally, if situations such as the inability to contact a subject continues, the principal investigator or sub-investigators will consider the subject as "lost to follow-up" and consider the subject's discontinuation of the study. The principal investigator or sub-investigators will use their best effort to re-contact the subject before deeming the subject as “lost to follow-up." Measures taken to contact the subject and potential reasons for the loss to follow-up will be recorded in source documents.

(1) If the principal investigator and sub-investigators determine that the discontinuation of the study is necessary
(2) If the subject requests to discontinue use of the investigational device
(3) If the subject withdraws consent
(4) If the subject develops a significantly severe adverse event
(5) If it is found after the start of the study that there is a violation of the inclusion and exclusion criteria and determined that compliance with the protocol is not possible
(6) If it is determined to be difficult to continue the study due to failures of specified study devices
(7) If serious deviations from the protocol are found

The main reasons for the discontinuation of the study should be documented in applicable case report forms. For subjects who do not receive surgical procedures, the seizure resolution rate 1 year after focal resection will not be evaluated.

15.5 Discontinuation of the Study

If the coordinating investigator learns, in reference to the following criteria, of any information concerning the quality, efficacy, or safety of specified study devices or any other important information on events that would interfere with the proper conduct of the study, the coordinating investigator will decide on the discontinuation of the study and notify the discontinuation and its reasons to the principal investigator and the head of the medical institution in written form.

(1) If it is determined to be difficult to continue the study due to new safety information (serious adverse events or failures)
(2) If the Central Evaluation Committee or Safety Evaluation Committee advises the discontinuation of the study
(3) If the medical institution, principal investigator, or sub-investigators commit a material violation of GCP or the protocol, and the event is deemed to interfere with proper continuation of the study
(4) If it is determined to be difficult to continue the study due to changes in the system for the execution of the study (e.g., personnel change of the coordinating investigator/principal investigator)
(5) Otherwise, if it is determined that the continuation of the study is inappropriate due to new information or changes in circumstances during the conduct of the study

16 STATISTICAL ANALYSIS

16.1 Determination of Target Sample Size

The planned number of enrollment for subjects who will use the investigational device is 37.

[Rationale]
For the p value of percentage agreement between the lateralization diagnosis of EP-01 and intracranial electrode placement, the null hypothesis will be $p = 0.8$, and the alternative hypothesis, $p > 0.8$. The hypothesis testing will be performed in the form of accurate one-tailed test of binomial distribution. For the anticipated percentage agreement of 0.97, a significance level of 0.025, and statistical power of 90%, the required sample size would be 34. In consideration of a 10% dropout, the target sample size was determined to be 37.

16.2 Data Handling Standards

Details regarding the handling of subjects are shown below.

(1) Non-intervention subjects
Of the enrolled subjects, those who do not use the investigational device will be considered non-intervention subjects

(2) Ineligible subjects
Subjects who do not meet the inclusion criteria or meet the exclusion criteria will be considered ineligible subjects.

(3) GCP non-compliant subjects
Subjects who have not been reviewed by the Investigational Review Board, with whom study agreements have not been entered into, or from whom appropriate informed consents have not been obtained will be considered GCP non-compliant subjects.

For unanticipated problems other than the above, the principal investigator will review the details and decide on analytical handling before the data is fixed.

16.3 Population Subject to Analysis

16.3.1 Safety Analysis Set (SAF)

The safety analysis set population is defined as the subject population that excludes from enrolled subjects non-intervention subjects.

16.3.2 Efficacy Analysis Set

(1) Full Analysis Set (FAS)
The full analysis set is defined as the population that excludes from enrolled subjects those who fall under the below.

- Non-intervention subjects
- Subjects with no efficacy data after use of the investigational device at all
- GCP non-compliant subjects

(2) Per Protocol Set (PPS)
The per protocol set is defined as the population that excludes from the full analysis set subjects who meet the following conditions.

- Ineligible subjects

16.4 Analysis of Subject Background

Continuous variables will be calculated with summary statistics and categorical variables with frequencies and rates.

16.5 Analysis of Primary Endpoint

The percentage agreement between the lateralization diagnosis of epileptic foci using EP-01 and that using intracranial electrodes, and the 95% confidence interval of the percentage of agreement by the Clopper-Pearson method will be calculated. The main subject population of the analysis will be the full analysis set.

16.6 Analysis of Secondary Endpoints

16.6.1 Efficacy Secondary Endpoints

- The percentage agreement between the lateralization diagnosis based on EP-01 EEG findings alone compared with lateralization diagnosis based on intracranial electrode EEG findings alone will be calculated.
- The rate of waveforms detected by EP-01 that are EEG will be calculated.
- The percentage agreement between lateralization diagnosis results based on EP-01 EEG findings combined with non-invasive findings and lateralization diagnosis results based on bilaterally-placed intracranial electrode EEG findings combined with non-invasive findings will be calculated.
- The percentage agreement between lateralization diagnosis results based on EP-01 EEG findings alone and lateralization diagnosis results based on bilaterally-placed intracranial electrode EEG findings alone will be calculated.
- The percentage agreement between lateralization diagnosis based on EP-01 EEG findings added to invasive findings and lateralization diagnosis results based on intracranial electrode EEG findings added to non-invasive findings will be calculated (evaluated by the principal investigator and others).
- The seizure resolution rate 1 year after focal resection will be calculated.
- The success rate of EP-01 placement will be calculated.

16.6.2 Safety Secondary Endpoints

For the presence of adverse events, adverse events attributable to the investigational device, serious adverse events, and serious adverse events attributable to the investigational device, the number of occurred cases, the number of affected subjects, and their rates will be calculated.

16.7 Interim Analysis

No interim analysis will be performed in this study.

17 COMPLIANCE WITH AND REVISION OF THE PROTOCOL

17.1 Compliance With and Deviation or Change From the Protocol

(1) The principal investigator and sub-investigators will not deviate or change from the protocol without obtaining written approval based on prior review by the Investigational Review Board.
(2) The principal investigator and sub-investigators will document all acts that deviate from the protocol.
(3) The principal investigator and sub-investigators may deviate or change from the protocol without prior approval of the Investigational Review Board for medically inevitable circumstances, such as avoidance of urgent risks to a subject. In such cases, the principal investigator will submit to the head of the medical institution and Investigational Review Board the details and reasons of the deviation or change and, if revising the protocol is appropriate, a draft of the revision, and obtain approval as soon as possible. The principal investigator will also obtain written approval from the head of the medical institution.
(4) The principal investigator will promptly submit a report to the head of the medical institution and the Investigational Review Board on various changes in the study that may materially affect the conduct of the study or increase risks to subjects.

17.2 Revision of the Protocol

If the principal investigator learns of any matters concerning the quality, efficacy, or safety of the investigational device or any other important information for the proper conduct of the study, the principal investigator will revise the protocol or explanatory documents as needed. After obtaining written approval based on prior review by the Investigational Review Board, the principal investigator will conduct the study in compliance with the revised protocol. However, this does not apply if the change is medically inevitable or if the change concerns administrative matters of the study (e.g., change of affiliation, job title, address, or telephone number).

18 QUALITY CONTROL AND QUALITY ASSURANCE OF THE STUDY

18.1 Quality Control

18.1.1 Direct Viewing of Source Documents

The principal investigator and medical institution must accept monitoring, audits, and investigations by the Investigational Review Board and regulatory authorities. In such cases, all study-related records, including source documents, must be made available for direct viewing upon request by monitors, persons in charge of auditing, Investigational Review Board, and regulatory authorities. When source documents are viewed, sufficient attention should be paid to protect the privacy of subjects.

18.1.2 Identification of Source Documents

In this study, the following source documents will be collated with entries in case report forms. These source documents should contain records of information (source data) that serve as the basis of case report forms.

(1) Medical records and records attached to them
(2) Test slips and worksheets
(3) Consent forms
(4) Tables for investigational device management, records of investigational device receipt and delivery

For comments in case report forms, the contents recorded in case report forms will be considered source data.

18.1.3 Data Management

The data management manager will manage the quality of accumulated data. Specifically, the data management manager will check for any inconsistencies within case report forms compared with data that medical institutions accumulated based on medical records and other source documents. If any doubts arise, inquiries on the data will be made to the principal investigator / sub-investigators as necessary. After finishing quality checks of data, the data will be fixed. Details should follow the Data Management Plan.

18.1.4 Monitoring

The principal investigator will facilitate monitoring to ensure that the human rights, safety, and welfare of subjects are protected; ensure that the study is conducted in compliance with the latest protocol, GCP, and ISO 14155; and confirm that the study data are accurate, complete, and verifiable when collated with source documents and other study-related records. The principal investigator will appoint a monitor to ensure that monitoring is properly conducted. The monitors will conduct monitoring in accordance with monitoring manuals prepared in advance.

18.2 Quality Assurance

Quality assurance is a planned and systematic activity established to ensure that the conduct, data preparation, documentation, and reporting of studies comply with GCP and related regulations. Independent of regular monitoring or the study's quality control operations, persons in charge of auditing appointed by the principal investigator will prepare, in accordance with the Manual on Auditing, audit reports and an audit certificates in which confirmed points concerning obtained data and study-related operations will be recorded, and then submit these to the principal investigator and the head of the medical institution. This operation will include external audits and confirmation of source documents. Additionally, direct viewing of source documents is necessary for the purpose of auditing.

19 ETHICS

19.1 Ethical Conduct of the Study

This study will be conducted in accordance with the ethical principles based on the Declaration of Helsinki; Act on Pharmaceutical and Medical Device; Ministerial Ordinance on Good Clinical Practice for Pharmaceuticals (Ministerial Ordinance No. 28; Ministry of Health, Labour and Welfare; March 27, 1997); and Ministerial Ordinance on Good Clinical Practice for Medical Devices (Ministerial Ordinance No. 36; Ministry of Health, Labour and Welfare; March 23, 2005); and in compliance with this protocol.

Consideration for the human rights, safety, and welfare of the subjects is of most importance and should be prioritized over scientific and social benefits.

The Investigational Review Board will sufficiently deliberate on the conduct and continuation of the study from ethical, scientific, and medical perspectives.

The head of the medical institution will take necessary measures to ensure that study at the medical institution is conducted properly and smoothly in accordance with the GCP ministerial ordinance, protocol, study agreements, and manuals of the medical institution. The principal investigator will conduct the study in accordance with the instructions and decisions of the head of the medical institution, which will be based on the approval of the Investigational Review Board, and in compliance with the protocol. In selecting subjects, the principal investigator and sub-investigators will consider the subject's health condition, symptoms, age, ability to consent, and dependency on the principal investigator or sub-investigators from the viewpoint of human rights protection and in accordance with the protocol. Persons who lack the capacity to consent will not be subjects, as a general rule, except when inevitable for the purpose of this study. Additionally, particularly careful consideration will be given when selecting as subjects persons considered to be in socially vulnerable positions.

The principal investigator will be responsible of making study-related medical decisions for subjects. Before the subjects participate in the study, the principal investigator and sub-investigators will sufficiently explain the contents of the study and other study-related matters to the subjects in advance in a manner that the subjects can understand, using the informational form and consent form, and obtain written voluntary consent on study participation. If information that may influence a subject's intention to continue or discontinue study participation is obtained, the principal investigator and sub-investigators will promptly inform the subject of such information and confirm the subject's intention to continue or discontinue study participation.

Records that may reveal the identity of subjects will be protected in a manner that gives regard to the protection of the subject's privacy and confidentiality.

19.2 Protection of Subject Confidentiality

The head of the medical institution will take necessary measures to ensure the protection of the subjects' confidentiality. Those involved in this study must pay the utmost attention to protect the privacy of subjects in subject-related information by, for example, prohibiting the use of information that could identify subjects. Additionally, persons involved in this study, including monitors and persons in charge of auditing, may not disclose to any third-party matters pertaining to the privacy of subjects, even if they learn of such information through direct viewing of source documents and other documents.

19.3 Explanation to and Consent of Subjects and Legal Representatives

19.3.1 Consent Form and Other Informational Forms

Prior to conducting the study, the principal investigator or sub-investigators will sufficiently explain, either orally or in written form, to subject patients or their legal representatives*, using the consent form and informational form approved by the Investigational Review Board, and obtain the patient's voluntary consent. When efficacy or safety-related information that may affect the consent of subjects or their legal representatives* is obtained, or when changes to the protocol that may affect the consent of subjects or their legal representatives* are made, the information will be promptly provided to the subjects or their legal representatives*, and the intention of the subjects or legal representatives* to continue or discontinue study participation will be confirmed in advance. In addition, the informational form and consent form will be revised after obtaining prior approval of the Investigational Review Board, and the re-consent of the subjects or legal representatives* will be obtained.

*Legal representatives are persons deemed justifiable in giving consent to study participation with or on behalf of a subject when the subject lacks sufficient capacity to give consent or when the subject is a patient under 18 years of age. They include persons who exercise parental authority over the patient, spouse of the patient, guardian of the patient, or other similar persons.

19.3.2 Informational Form for Assent and Other Informational Forms

Prior to conducting a study, the principal investigator or sub-investigators will sufficiently explain, either orally or in written form, to those among subjects patients who are underage or require assistance in understanding informational forms, using the informational form for assent approved by the Investigational Review Board, and obtain the patient's voluntary assent. Whenever possible, informed assents will be obtained in written form. When efficacy or safety-related information that may affect the intention of subjects is obtained, or when changes to the protocol that may affect the intention of subjects are made, the information will be promptly provided to the subjects and the intention of the subjects to continue or discontinue study participation will be confirmed in advance. In addition, the informational form for assent will be revised after obtaining prior approval of the Investigational Review Board, and the assent of the subjects will be obtained again.

19.3.3 Contents of Informational Form and Assent Form

The informed consent form should give consideration to expressions that are easy to understand for patients and their legal representatives. Descriptions should include the following explanation points. The informational form for assent should describe the explanation points in simple expressions that subject persons can understand.

(1) That the study is accompanied by research
(2) Purpose of the study
(3) Name, job title, and contact information of the principal investigator
(4) Method of the study
(5) Anticipated clinical benefits and risks or disadvantages (if there are no anticipated benefits to the subject, such fact should be notified to the subjects)
(6) Matters related to other treatment methods
(7) Duration of study participation
(8) Ability to withdraw from the study at any time
(9) That subjects will not be treated disadvantageously by not participating in or by withdrawing from the study
(10) Matters related to the handling of the investigational device when withdrawing from study participation
(11) That monitors, persons in charge of auditing, and the Investigational Review Board will be able to view source documents, provided that the confidentiality of subjects is protected
(12) That confidentiality pertaining to subjects will be protected
(13) Contact information of the medical institution in the event of health hazards
(14) That necessary treatment will be provided in the event of health hazards
(15) Matters related to compensation for health hazards
(16) Matters related to the Investigational Review Board, which will investigate and deliberate on the appropriateness of the study
(17) Matters related to study costs that subjects will bear
(18) Matters that subjects should comply with

19.3.4 Timing and Method of Obtaining Consent and Assent

Written consent and assent for participation in this study must be obtained prior to conducting the provided screening tests or evaluations of this study. Written voluntary consent of a subject patient himself/herself or the legal representative on study participation will be obtained after sufficiently explaining to the subject patient himself/herself or the legal representative using written forms and confirming that the patient or legal representative adequately understood the contents.

The principal investigator or sub-investigators who provided the explanation and the patient himself/herself will sign the consent form and assent form and respectively indicate dates. If persons cooperating in the study provide supplementary explanations, they will also sign the consent form and indicate dates. The

original consent form should be retained as source document and a copy should be handed to the patient along with the informational form, except where there are special arrangements at the medical institution.

19.3.5 Considerations Concerning Consent

Patients who have difficulty writing should also sign the consent form and indicate dates themselves as much as possible. The consent form and informational form will be handed to patient, the contents will be explained orally or by other means of communication, the patient will orally consent to study participation, and further, the legal representative or witness* will sign the consent form and indicate the date himself/herself. The field for the name of the patient himself/herself will be left blank, and the person writing on behalf of the patient (witness) or otherwise, the principal investigator or a sub-investigator will indicate the name of the patient, the background of the patient's inability to sign the form in his/her own handwriting, and the relationship between the patient and the witness in the margin or elsewhere.

*: A person who is independent of the conduct of the study and will not be unduly affected by those involved in the study that is present during the informed consent process. The principal investigator, sub-investigators, and persons cooperating in the study do not apply.

19.3.6 Revision of Informational Form for Consent and Assent

If new important information that may affect a subject's intention on study participation is obtained (information considered important for subjects in general that requires revision of the consent form, informational form, and informational form for assent), the principal investigator will promptly revise the consent form and informational form and obtain a notification on the decision of the head of the medical institution, which will be based on the approval of the Investigational Review Board. Additionally, for subjects already participating in the study as well, the principal investigator will promptly explain again using the revised consent form and informational form, and obtain consent in written form on the continuance of study participation. However, consent is not required for subjects who have already completed observations at the time of revision.

If information that may affect a subject's intention to continue study participation is obtained (information concerning the progress of the study for individual subjects participating in the study, such as fluctuations in laboratory values), the principal investigator or sub-investigators will promptly inform the subject of the information and confirm the subject's intention to continue study participation. Additionally, the date the information was communicated to the subject, the contents of information communicated, and the results of the confirmation should be recorded in medical records and other source health care records.

19.4 Investigational Review Board

Prior to conducting this study, the human rights, safety, and welfare of subjects will be ensured by having the Investigational Review Board of the medical institution deliberate and approve the protocol and various materials used to obtain the subjects' consent.

19.5 Central Evaluation Committee

The principal investigator will establish a Central Evaluation Committee for the purpose of third-party evaluation of efficacy. The Central Evaluation Committee will evaluate the lateralization diagnosis of epileptic foci in accordance with the Manual on Central Evaluation Committee.

Purpose and timing of efficacy evaluation	The purpose is to use third-party evaluation for lateralization diagnosis of epileptic foci in order to avoid suspicion of biased evaluation.<br>The evaluation will be performed after the principal investigator and others finish determining lateralization diagnosis results on epileptic foci.<br>Timing: Evaluations will be performed each time after case data is sent from respective medical institutions to the central evaluation physician via the Central Evaluation Committee secretariat.

In the above operations, if the Central Evaluation Committee determines it is necessary to hear the opinions of other experts, it will contact the coordinating investigator and then request other experts to participate as observers and hear their opinions.

19.6 Safety Evaluation Committee

The coordinating investigator will establish a Safety Evaluation Committee for the purpose of conducting a third-party safety evaluation. The Safety Evaluation Committee will evaluate safety based on data obtained at respective medical institutions in accordance with the Manual on Safety Evaluation Committee.

Table 19-1. Evaluation Items of the Safety Evaluation Committee

Purpose and timing of safety evaluation	The purpose is to evaluate safety and the appropriateness of continuing the study.<br>(1) Periodic evaluation<br>The presence, severity, relationship to devices of adverse events* determined based on image tests taken at respective medical institutions will be evaluated.<br>*Adverse events determined by imaging testing: hemorrhage, midline shift, cerebral spinal fluid leak<br>The safety evaluation will be conducted at the timing of periodic meetings specified in the protocol after CT image data is submitted by the respective medical institutions to the study coordinating office.<br>・ First periodic meeting: coordinated when data of 15 to 20 cases (note: modified according to the number of cases) is submitted by respective medical institutions to the Safety Evaluation Committee secretariat.<br>・ Second periodic meeting: when data of all cases is submitted by respective medical institutions to the Safety Evaluation Committee secretariat.<br><br>(2) Non-periodic evaluation<br>Meetings will be held as non-periodic meetings if the coordinating investigator deems it necessary based on the occurrence of adverse events, such serious adverse events caused by the investigational device, and safety information provided by the study device provider.<br>Recommendations on the continuance of the study and changes to the protocol will be provided if the coordinating investigator deems it necessary based on serious adverse events attributable to the investigational device that are reported by respective medical institutions and on the following.<br>• When new important safety information concerning the continuation of the study overall is obtained<br>• When an adverse event (or adverse reaction) or failure that substantially exceeds initial projections occur<br>• When efficacy is significantly more or less effective than initially projected<br>• When new significant information regarding safety or efficacy is obtained<br><br>(3) Other<br>Held when the coordinating investigator deems it otherwise necessary.

In the above operations, if the Safety Evaluation Committee determines it is necessary to hear the opinions of other experts, it will contact the coordinating investigator and then request other experts to participate as observers and hear their opinions.

19.7 Conflict of Interest

19.7.1 Provision of Research Funds

This study will be conducted with aid received from Epsilon Medical, Inc. and the program for the investigator initiated multicenter clinical trial of endovascular EEG devices (principal investigator: Yuji Matsumaru, University of Tsukuba) supported by Research on Development of New Medical Devices, Japan Agency for Medical Research and Development.
In conducting this study, the principal investigator and sub-investigators will voluntarily report conflicts of interest in accordance with the regulations on conflicts of interest separately set forth by medical institutions.

19.7.2 Provision of Investigational Device

This study will be conducted by receiving for free of charge the investigational device of the study (EP-01) and other specified study devices (EX-01 extension wire) from Epsilon Medical, Inc. based on agreements with Epsilon Medical, Inc. for the investigator-initiated clinical trial. Epsilon Medical, Inc. will also provide safety information on the investigational device (EP-01).

19.7.3 Provision of Data Acquired in the Study

Regarding data obtained in this study, those for which the subject's voluntary consent is obtained will be provided to Epsilon Medical, Inc. pursuant to the agreement between University of Tsukuba and Epsilon Medical, Inc..

Epsilon Medical Inc. will use the study data for the purposes of investigating adverse events and failures, confirming the safety and efficacy of Epsilon Medical, Inc. device products, and improving Epsilon Medical, Inc. research and development, limited to when the coordinating investigator provides instructions and permission.

20 RECORDS RELATED TO THE STUDY AND RETENTION

20.1 Case Report Form

20.1.1 Format of the Study's Case Report Form

For all subjects who have consented to participation in this study, the principal investigator and sub-investigators will promptly record data on case report forms after each observation of subjects in a manner that is consistent with medical records and other source documents. If the data is recorded by persons cooperating in the study, the recording will be limited to transcription from source documents. The principal investigator will confirm that all contents recorded in case report forms are accurate and complete, and will attest to them by signing the case report forms.

20.2 Retention of Records

20.2.1 Principal Investigator

The principal investigator will retain the following records in accordance with the Manual on Record Retention.

(1) Protocol, clinical study report, case report forms, and other documents prepared by the principal investigator or sub-investigators in accordance with the provisions of the GCP Ministerial Ordinance
(2) Documents on the opinions of the Investigational Review Board notified by the head of the medical institution, and other records obtained from the head of the medical institution in accordance with GCP and ISO 14155
(3) Records of monitoring, audits, and other operations related to the preparation and management of the study
(4) Data obtained by conducting the study
(5) Records on the investigational device
(6) Records on audit operations

20.2.2 Medical Institution

The head of the medical institution will appropriately retain study-related records (including documents) that should be retained at the medical institution until either the date when 10 years have passed since the date marketing approvals for the investigational device is obtained or since the discontinuation or termination of the study, or the date set forth by the medical institution, whichever arrives later. In the event that the retention period is extended, arrangements will be separately made.

The main records that must be retained at medical institutions include the following.

(1) Source documents (documents, data, and records on which case report forms are based)
Hospital records, medical records, laboratory notes, memos, records of use of the investigational device, records of use of specified study devices, record data of automated instruments, copies or transcriptions that have been verified as being accurate copies, photographic negatives, microfilms or magnetic media, x-ray photographs, subject files and study-related records retained in respective laboratory rooms, test slips including outsourced measurements, worksheets, clinical research coordinator records, records verified with other departments/hospitals
(2) Agreements, consent forms and informational forms, assent forms, and other documents prepared by persons working for the medical institution in accordance with GCP provisions or their copies; study agreements; discontinuation and termination reports; informational forms and consent forms (including revised versions); and signed and dated consent forms (including revised versions)
(3) Protocol (including revised versions), documents obtained from the Investigational Review Board, and other documents obtained in accordance with the GCP provisions; investigator's brochure (including revised versions); investigator's standard operating procedures (SOP); documents and information provided by the principal investigator; items reported by the principal investigator (e.g., changes in the protocol, adverse event reports); and the Investigational Review Board's SOP, committee registry, review documents, deliberation records, and reports on review results
(4) Records of investigational device management and other study-related operations
(5) Procedures for collection of raw material tissue, Manual on Study Device Management, investigational device management table, investigational device delivery request forms, and investigational device receipt and collection slips

21 COMPENSATION FOR HEALTH DAMAGE IN SUBJECTS AND REDUCTION OF BURDEN OF STUDY PARTICIPATION

21.1 Compensation for Health Hazards

If any health hazards occur to subjects as a result of this study, appropriate compensation will be provided to the subjects according to the extent and duration. However, in the case of willful misconduct or gross negligence on the part of the medical institution, the handling of such cases will be discussed based on agreements with the medical institution. Compensations will be processed in the following procedures.

(1) If a subject experiences any health hazards as a result of this study, the medical institution will take necessary measures including treatment and inform the principal investigator.

(2) If a subject claims or may claim compensation or indemnification to the medical institution for the subject's health hazards caused by this study, the medical institution will immediately contact the principal investigator, and both parties will cooperate in resolving the event.

(3) If a subject experiences health hazards attributable to the study, the principal investigator will compensate in accordance with the Manual on Compensation for Health Damage in Subjects set forth by the principal investigator.

(4) The principal investigator will bear compensations or indemnifications for health hazards and other costs incurred for the resolution in accordance with the provisions in study agreements.

(5) If a subject experiences health hazards as a result of this study and liabilities subsequently arise, indemnifications will be provided under the responsibility and at the expense of the party responsible for such liabilities.

(6) The principal investigator will take necessary measures, including insurance, for any compensations or indemnifications described above.

21.2 Reduction of Subject Burden of Study Participation

The investigational device used in this study will be provided for free of charge by the study device provider, Epsilon Medical Inc. This study will be conducted primarily within the scope of hospitalization, testing, and diagnostic imaging performed in regular clinical practice. However, any costs for investigational device placement and removal procedures and costs for testing and diagnostic imaging that additionally arise due to study participation will be borne by the medical institution. Other medical care expenses will be borne by subjects based on the subjects' health insurance. Payment to reduce the subjects' burden of study participation will be paid to subjects whose consent has been obtained, limited to the date of tests provided in this protocol as a general rule.

22 ARRANGEMENTS ON PUBLIC DISCLOSURE

Information regarding this study will be registered on the system for clinical research submission and public disclosure (Japan Registry of Clinical Trials) before initiating subject recruitment.
The results of this study will be organized by the coordinating investigator as a clinical study report. The clinical study report will be prepared in phases, with the evaluation results up to the post-observation period set as primary analysis results and the evaluation results up to the evaluation of epileptic seizure outcomes set as secondary analysis results. Additionally, the results will be publicly disclosed as needed as journal articles or presentations at academic society meetings. The University of Tsukuba will own the rights to the results of the study conducted in accordance with this protocol. Public disclosure of study results by the principal investigator, sub-investigators, and medical institution will be conducted under agreement with the coordinating investigator. Additionally, when publicly disclosing study results, sufficient consideration will be given to the protection of the subjects' privacy, including not directly disclosing the subjects' names.

23 PLANNED STUDY PERIOD

Investigational device evaluation period
March 2024 to August 2025
Period including evaluation period of epileptic seizure outcomes following epilepsy focal resection
March 2024 to August 2026

24 REFERENCE

1) 日本神経学会. てんかん診療ガイドライン 2018
2) 大槻泰介. 平成 25 年てんかんの有病率等に関する疫学研究及び診療実態の分析と治療体制の整備に関する研究. 厚生労働省科学研究費補助金 総合研究報告書.
3) Dario J. Englot et al. A Modern Epilepsy Surgery Treatment Algorithm: Incorporating Traditional and Emerging Technologies. Epilepsy Behav. 2018 March ; 80: 68–74.
4) 岩崎真樹.てんかん外科における頭蓋内脳波の適応. 脳神経外科, 47(1) : 5 - 14, 2019
5) Khoo HM, Kishima H et al. Technical Aspects of SEEG and Its Interpretation in the Delineation of the Epileptogenic Zone. Neurol Med Chir (Tokyo). 2020 Dec 15;60(12):565-580.
6) 前澤聡ら.てんかん外科の現状と展望.現代医学 69 巻 2 号.令和 4 年 12 月(2022)
7) 渡辺英寿.内側側頭葉てんかんの診断と手術適応に関するガイドライン.日本てんかん学会
8) 渡辺英寿.わが国におけるてんかん外科の現状.脳神経外科速報 17(1) :74-81,2007.
9) Crandall Phet al. Clinical applications of studies on stereotactically implanted electrodes in temporallobe epilepsy. J Neurosurg. 1963 Oct;20:827-40.
10) Fujimoto A et al. False lateralization of mesial temporal lobe epilepsy by noninvasive neurophysiological examinations. Neurol Med Chir (Tokyo). 2006 Oct;46(10):518-21.
11) Engel J Jr, Wiebe S et al. Practice parameter: temporal lobe and localized neocortical resections for epilepsy. Epilepsia. 2003 Jun;44(6):741-51.
12) Wiebe S, Blume WT, Girvin JP, Eliasziw M; Effectiveness and Efficiency of Surgery for Temporal Lobe Epilepsy Study Group. A randomized, controlled trial of surgery for temporal-lobe epilepsy. N Engl J Med. 2001 Aug 2;345(5):311-8.
13) 中瀬裕之ら.脳血管内脳波にて棘波をとらえた側頭葉てんかんの 2 例.脳神経 45(10):973-977,1993.
14) H. G. Wieser, W. T. Blume et al. Commission Report Commission on Neurosurgery of the International League Against Epilepsy (ILAE) 1997–2001

25 APPENDIX

25.1 Appendix 1. ILAE Classification

The following shows the ILAE classification for evaluation of epileptic seizure outcomes after surgical treatment.

ILAE Classification

ClassI	Completely seizure free; no auras
ClassII	Only auras; no other seizures
ClassIII	One to three seizure days per year; ± auras
ClassIV	Four seizure days per year to 50% reduction of baseline seizure days; ± auras
ClassV	Less than 50% reduction of baseline seizure days to 100% increase of baseline seizure days; ± auras
ClassVI	More than 100% increase of baseline seizure days; ± auras

Reference:

14）ILAE Commission Report

HUMAN SCIENCE http://www.science.co.jp

TRANSLATION CERTIFICATE

**Protocol Number：**	**Source Language**	**Target Language**
HS_MED_20241004-01	Japanese	English

Name/Description of source document(s)
Source Document：
240523_EPSILON IE_CIP_2.1.pdf

Outcome Document：
240523_1_EPSILON IE_CIP_2.1.docx

Certificate Statement
Translation：Our company translated the source file(s) from the source language into the target language listed above. As the translation company, we are qualified to translate from the source language into the target language on the subject matter contained within the source file. To the best of our knowledge and ability, the translation is a true and accurate translation of source file.

Representative Name：
Tomoyuki Sano
Project Manager
Medical Group
Language Solutions Department
Human Science Co., Ltd.
2-7-1, Nishi-shinjuku, Shinjuku-ku, Tokyo 160-0023, Japan

Date of Translation： September 30, 2024

難治性てんかん患者を対象とする血管内脳波測定デバイスの有効性および安全性を確認する多施設前向き単群試験
-EPSILON IE-

(Endovascular EEG Device Prospective Multicenter Single-arm clinical trial to confirm efficacy and safety performance on Intractable Epilepsy patients)

治験実施計画書

治験調整医師：
筑波大学附属病院　脳卒中科
松丸　祐司

治験実施計画書番号：EPSILON IE
版番号：第 2.1 版
作成年月日：2024 年 5 月 23 日
情報公開番号：jRCT2032230720

【本書の取り扱いに関して】
本治験実施計画書は、治験の直接関係者（実施医療機関の長、治験責任医師、治験分担医師、治験協力者、治験機器管理者、治験使用機器管理者、治験審査委員会及び中央判定委員会等）に限定して提供するものですので、本治験実施計画書の記載内容について、第三者に漏洩しないように秘密情報としてお取り扱い頂きますようお願い致します。

本治験は、ヘルシンキ宣言に基づく倫理的原則、医薬品医療機器等法、医療機器の臨床試験の実施の基準に関する省令（平成 17 年 3 月 23 日付厚生労働省令第 36 号）、ISO 14155 に従い、本治験実施計画書を遵守して実施する。
本治験は、治験に関わる法律及び規制並びにガイドラインにも準拠する。

また、本治験を安全に実施するために必要と判断される場合には、治験実施計画書の改訂を行うものとする。

作成・改訂履歴

作成日	版数	改訂内容
2023 年 12 月 26 日	第 1.0 版	初版作成
2024 年 1 月 26 日	第 1.1 版	誤記修正<br>中央判定委員会への提出データの詳細追記
2024 年 3 月 22 日	第 2.0 版	誤記修正、記載整備<br>資金提供者の追加、症例取り扱い記載整備<br>併用薬・併用療法の詳細追記<br>有害事象取り扱い詳細追記<br>中央判定委員会および安全性評価委員会への提出データの詳細追記
2024 年 5 月 23 日	第 2.1 版	誤記修正、記載整備<br>ISO14155 に従った記載整備<br>ISO14155 に従った追記（治験参加に伴うリスク・ベネフィット、治験使用機器の精度管理確認方法、評価項目設定根拠）

1　治験実施計画書の要約

治験課題名	難治性てんかん患者を対象とする血管内脳波測定デバイスの有効性および安全性を確認する多施設前向き単群試験
治験の目的	血管内脳波測定デバイス EP-01 を用いた難治性てんかん患者のてんかん焦点の側方性診断に対する有効性について検討する。また、EP-01 を使用した際の安全性について評価する。
治験デザイン	多施設共同前向き単一群試験<br>本治験は複数施設で実施する。最長 30 日のスクリーニング期間、最大 2 週間の観察期間及び 1 週間の後観察期間で構成され、被験機器に関する評価を行う。更に、てんかん焦点切除術を行った患者はてんかん切除術より約 1 年後にてんかん発作消失率を評価する。
開発ステージ	検証的試験
被験機器<br>識別記号	血管内脳波測定デバイス<br>被験機器識別記号：EP-01<br>被験機器 EP-01 は EP-01 本体に加えて EP-01 付属品 (接続コネクターとシースセット、ワイヤー固定具、インサーター、ロングシース、ワイヤー用ウィング、ディスペンサー) より構成される。被験者一名につき、EP-01 本体および接続コネクターは症例に応じて最大 6 本、シースセットは 2 個、ワイヤー固定具、インサーター、ロングシース、ワイヤー用ウィングは必要に応じて最大 2 個使用する。
その他の治験<br>使用機器	・EX-01 エクステンションワイヤー<br>EP-01 本体からマイクロカテーテルを抜去する際に EP-01 本体の全長を一時的に延長するために使用する。<br>機器提供者：株式会社 Epsilon Medical<br>・脳波計　EEG1200 シリーズ　ニューロファックス<br>認証番号：218AHBZX00013000<br>製造販売業者：日本光電株式会社<br>EP-01 と接続して脳波測定を行う
対象被験者	頭蓋内電極留置術を施行する難治性てんかん患者
選択基準	登録時に、以下の基準すべてを満たす患者を選択する。<br>(1) 難治性てんかん患者のうち、側頭葉てんかん、前頭葉てんかん、頭頂葉てんかん、後頭葉てんかん、島回てんかん患者<br>(2) 頭蓋内電極を留置する患者<br>(3) 海綿静脈洞・横静脈洞・上矢状静脈洞・下錐体静脈洞などてんかん焦点に近い血管に対し、左右の頸静脈から EP-01 を脳内に誘導できる血管解剖を有する患者［EP-01 本体の留置血管は医師の判断とする。ただし脳静脈の両側の対称位置へ誘導出来る事が確認できている事は必須とする］<br>(4) 15 歳以上 70 歳未満（同意取得時）

除外基準	登録時に、以下の基準いずれかに該当する患者を除外する。<br>(1) 全般発作のみを有するてんかん患者<br>(2) 内頸静脈に閉塞・血栓がある患者<br>(3) 出血素因および凝固異常がある患者<br>(4) 手術時に安静を保つことが困難である患者<br>(5) 重篤な造影剤アレルギーを有する患者<br>(6) 金属アレルギーを有する患者<br>(7) 妊娠あるいは妊娠の可能性のある患者<br>(8) 他の治験に参加している患者<br>(9) 責任医師または分担医師が本試験への参加を不適切と判断した患者
評価項目	【主要評価項目】<br>(1) EP-01 によるてんかん焦点の側方性の診断能（頭蓋内電極による診断結果との一致割合）：中央判定委員会による第 3 者評価<br>・ 臨床所見および同意取得日から過去 1 年以内に得た画像所見、頭皮電極による脳波所見（以下、非侵襲的所見）に、EP-01 による脳波所見を加えたてんかん焦点の側方性（以下、側方性）診断結果と、非侵襲的所見に頭蓋内電極による脳波所見を加えた側方性診断結果とを比較し、一致する割合を算出する。<br><br>【副次評価項目】<br>(1) EP-01 のみによるてんかん焦点の側方性の診断能：中央判定委員会による第 3 者評価<br>・ EP-01 脳波所見のみによるてんかん焦点の側方性診断結果と、頭蓋内電極脳波所見のみによる側方性診断結果とを比較し、一致する割合<br>(2) EP-01 による波形の脳波同定<br>・ EP-01 で検出された波形が脳波によるものであるか、開閉眼時の脳波所見との比較評価：中央判定委員会による第 3 者評価<br>・ EP-01 のみを留置した期間がある場合は、EP-01 のみと、EP-01 および頭蓋内電極を入れた状態での脳波測定性能について比較評価：治験責任医師等による評価<br>(3) EP-01 によるてんかん焦点の側方性の診断能（両側に留置した頭蓋内電極による診断結果との一致割合）：中央判定委員会による第 3 者評価<br>・ EP-01 脳波所見および非侵襲的所見によるてんかん焦点の側方性診断結果と、両側に留置した頭蓋内電極脳波所見および非侵襲的所見による側方性診断結果とを比較し、一致する割合<br>・ EP-01 脳波所見のみによるてんかん焦点の側方性診断結果と、両側に留置した頭蓋内電極脳波所見のみによる側方性診断結果とを比較し、一致する割合<br>(4) EP-01 によるてんかん焦点の側方性の診断能（頭蓋内電極による診断結果との一致割合）：治験責任医師等による評価<br>・ 非侵襲的所見に、EP-01 の脳波所見を加えた側方性診断結果と、非侵襲的所見に頭蓋内電極の脳波所見を加えた側方性診断結果とを比較し、一致する割合を算出する。<br>(5) 焦点切除後 1 年後のてんかん発作消失率<br>・ ILAE 分類の分類を用いて評価を行う<br>(6) EP-01 留置成功率（%）<br>・ 留置直後の EP-01 本体留置本数／マイクロカテーテル留置本数×

	100 を算出する<br>・　抜去直前に EP-01 が留置目的血管からの移動有無評価を行う<br>(7)　安全性の評価<br>・　被験機器を起因とする重篤な有害事象（EP-01 留置および抜去のための医学的行為を含む）および画像検査等より判断する有害事象*：安全性評価委員会による第 3 者評価<br>*画像検査等より判断する有害事象例：出血、正中偏移、髄液漏<br>・　有害事象の発現［発現期間：被験機器使用開始から後観察期間終了まで。被験機器全て（EP-01 本体および EP-01 付属品）との関連性、EP-01 留置術、抜去術のための医学的行為との関連性、その他の治験使用機器との関連性、程度、重篤性、処置、転帰］<br>(8)　その他の評価<br>・　被験機器 EP-01 の不具合（EP-01 本体および EP-01 付属品の不具合）<br>・　その他の治験使用機器の不具合（EX-01 エクステンションワイヤー/脳波計）
試験方法	原則として WADA テストまたは頭蓋内電極留置術と同時に EP-01 を留置する。なお、EP-01 本体の脳静脈への留置と、頭蓋内電極留置術を別日で実施する場合は、3 日間以内に行う。発作症状が得られるまで最大 2 週間観察し、焦点の側方性が確認された後、EP-01 本体を抜去する。頭蓋内電極および EP-01 から得られた脳波波形を基に、てんかん焦点の側方性診断を実施する。
併用制限、併用禁止薬・療法	頭蓋内電極留置術に合わせ、必要に応じて医師判断にて通常臨床で実施される断薬および減薬を行う。
予定被験者数	37 例
治験実施予定期間	被験機器評価期間<br>2024 年 3 月～2025 年 8 月<br>てんかん焦点切除術によるてんかん発作転帰評価期間を含めた期間<br>2024 年 3 月～2026 年 8 月

スクリーニング期間	治験検査期間	後観察期間	てんかん発作転帰評価期間
（30 日～1 日前）	（2 週間）	（1 週間）	外科手術から 1 年

同意取得　　被験機器留置期間　　被験機器評価期間終了　　てんかん発作転帰評価期間終了

図　治験のアウトライン

表　検査・観察項目及びスケジュール

項目	スクリーニング期間	被験機器評価期間								てんかん発作転帰評価
		治験検査期間						後観察期間		
時期	スクリーニング		被験機器留置[10]	被験機器留置1日後	頭蓋内電極留置	被験機器留置1週間後	被験機器抜去[11]/中止時[15]	被験機器抜去1日後	被験機器抜去1週間後/中止時[15]	外科的治療から1年後[12]
許容範囲	被験機器留置前30日～1日				被験機器留置±3日以内		被験機器留置から14日以内[13]	＋3日	＋3日	±60日
同意取得	●									
登録		●								
適格性確認	●									
被験者背景調査[1]	●									
身長・体重[2]	●									
頸部エコー[3]	●									
血液検査[4]	●									
非侵襲的所見[5]: てんかん発作臨床所見	●									
非侵襲的所見[5]: 画像所見	●									
非侵襲的所見[5]: 頭皮電極	●									
画像検査: MRI検査[6]	●									
画像検査: X線検査[7]			●	●		●	●			
画像検査: CT[8]	○[14]		●	●		●	●	●	●	
脳波検査[16]			←―	―――	―――	―――	―→			
有害事象			←―	―――	―――	―――	―――	―――	―→	
不具合			←―	―――	―――	―――	―→			
原疾患治療・抗血栓療法[9]	←―	―――	―――	―――	―――	―――	―――	―――	―→	
併用薬			←―	―――	―――	―――	―――	―――	―→	
てんかん発作評価（ILAE分類）										●

●：本治験で必須となるデータまたは観察項目　○：本治験目的での実施は不要

1) 同意取得時年齢、性別、血圧、脈拍、原病歴、合併症、既往歴、喫煙歴、飲酒歴を収集する。
2,3) 同意取得前 90 日以内に実施していれば、再測定不要とし、同意取得前のデータを収集する。
4) 同意取得前 90 日以内に実施していれば、再測定不要とし、同意取得前のデータを収集する。血小板数、APTT、PT-INR　の検査を実施する。
5) 被験者状態に応じ、治験責任医師等の判断によりてんかん焦点側方性診断に必要なもののみ調査する。
6)単純 3D-T1WI および、MRV または造影 3D-T1WI を実施する。同意取得前 90 日以内に実施していれば、再測定不要とし、同意取得前のデータを収集する。
7) X 線検査（正面・側面）を実施する。検査のタイミングは、被験機器留置直後・被験機器留置翌日・被験機器留置 1 週間後（被験機器留置 1 週間以内にてんかん発作発現していれば不要）・被験機器抜去直前に実施する。
8) EP-01 留置直後の撮像方法としてマルチスライス CT に限らずコーンビーム CT（CBCT）での撮像でも可。CT 検査のタイミングは、被験機器留置直後・被験機器留置翌日・被験機器留置 1 週間後（被験機器留置 1 週間以内にてんかん発作発現していれば不要）・被験機器抜去直前に実施する。スライス厚 1mm 以下および 3~5mm の 3D データを安全性評価委員会へ提出する。
9) 原疾患に対する治療（外科的手術歴含む）および抗血栓療法に関しては、同意取得以前 から後観察期間終了までを収集する。
10) 原則、WADA テストまたは、頭蓋内電極留置と同手術内で被験機器の留置を行う。
11) てんかん発作が発現するまで、または、最大 14 日間（＋3 日）被験機器を留置する。被験機器と頭蓋内電極抜去、外科的治療を同日にて実施することも可能。被験機器の先端位置の移動が疑われた場合は、被験機器の抜去判断のためすみやかに X 線検査を実施する。
12) てんかん切除術を行った被験者のみ実施。
13) 各実施医療機関の体制上、最大 2 週間の抜去が難しい場合は、+3 日間のアロワンスを設けることを可能とする。
14) 通常診療で被験機器留置前 90 日以内に実施している場合のみ、安全性評価委員へ提供する。実施していない場合の再撮像は不要。
15) 治験検査期間中に中止する場合、被験者の状況に応じて被験機器抜去時に定められた検査を可能な限り実施した後、被験機器の抜去を実施し、後観察期間へ移行する。後観察期間中に中止する場合、被験者の状況に応じて被験機器抜去後 1 週間後に定められた検査を可能な限り実施した後、治験終了とする。
16) EP-01 のみまたは EP-01 と頭蓋内電極の両方が留置されている期間、EP-01 および頭蓋内電極による脳波をビデオ監視下で測定する。開閉眼時の脳波データ（開閉眼時の脳波データ取得タイミングは治験責任医師等の判断とする）およびてんかん発作時脳波データを中央判定委員会へ提出する。

2 目次

1 治験実施計画書の要約 3
2 目次 8
3 略号及び用語の定義一覧 12
4 開発の経緯 13
4.1 開発の背景 13
4.2 デバイスの臨床的位置づけ 13
4.3 臨床試験成績 15
4.4 被験者に対する既知および可能性のある危険と利益の要約 15
4.4.1 被験製品 EP-01 を使用する際のリスク 15
5 本治験の目的 16
6 対象被験者の選択 16
6.1 対象被験者 16
6.2 選択基準 16
6.3 除外基準 17
7 治験使用機器 18
7.1 被験機器 18
7.1.1 被験機器の名称又は識別記号 18
7.1.2 EP-01 本体 18
7.1.3 EP-01 付属品 18
7.1.4 被験機器包装及び表示 19
7.2 被験機器の交付・管理・出庫手順 19
7.2.1 被験機器取扱い上の注意 19
7.2.2 被験機器の廃棄 19
7.3 その他の治験使用機器 19
7.3.1 EX-01 エクステンションワイヤー／株式会社 Epsilon Medical 19
7.3.2 脳波計　EEG1200 シリーズ　ニューロファックス／日本光電工業株式会社 20
8 被験者の登録 21
8.1 登録手順 21
8.2 治験に組み入れられた被験者の定義 21
9 治験の方法 22
9.1 治験デザイン 22
9.2 診断 22
9.3 治験使用機器の用法および留置期間 22
9.3.1 被験機器の留置方法 22
9.3.2 頭蓋内電極の留置方法 23
9.3.3 被験機器留置のタイミング 23
9.3.4 被験機器の留置期間中 23
9.3.5 被験機器の抜去 23
9.3.6 頭蓋内電極の抜去 23
10 被験者の管理 23
10.1 前治療薬剤及び療法 23
10.2 併用薬及び療法 23
10.2.1 抗血栓療法 24
10.2.2 併用禁止および制限薬及び療法 24
10.2.3 併用注意薬および療法 24
11 検査・観察項目及びスケジュール 25
11.1 観察項目 25
11.1.1 被験者患者背景 25

11.1.2 身長・体重 25
11.1.3 頸部エコー 25
11.1.4 血液検査 25
11.1.5 非侵襲的所見 25
11.1.6 MRI 検査 26
11.1.7 マイクロカテーテル留置場所の確認 26
11.1.8 EP-01 留置場所の確認 26
11.1.9 CT 検査 26
11.1.10 脳波検査 26
11.1.11 有害事象の観察 27
11.1.12 不具合の観察 27
11.1.13 併用薬及び療法 27
11.1.14 ILAE の分類 28
11.1.15 中央判定委員会による評価 28
11.1.16 安全性評価委員会による評価 28
11.2 検査・観察項目及びスケジュール 29
12 評価項目 32
12.1 主要評価項目 32
12.2 副次評価項目 32
12.2.1 EP-01 のみによるてんかん焦点の側方性の診断能：中央判定委員会による第三者評価 32
12.2.2 EP-01 による波形の脳波同定 32
12.2.3 EP-01 によるてんかん焦点の側方性の診断能（頭蓋内電極による診断結果との一致割合）：中央判定委員会による第 3 者評価 32
12.2.4 EP-01 によるてんかん焦点の側方性の診断能：治験責任医師等による評価 33
12.2.5 焦点切除後 1 年後の発作消失率 33
12.2.6 EP-01 留置成功率（%） 33
12.2.7 安全性の評価 33
12.2.8 その他の評価 34
13 有害事象及び不具合発生時の取扱い 35
13.1 有害事象発生時の被験者への対応 35
13.2 重篤な有害事象及び不具合の報告 35
13.2.1 重篤な有害事象および重篤な健康被害発生のおそれがある不具合の定義 35
13.2.2 重篤な有害事象及び不具合の報告対象 36
13.2.3 重篤な有害事象及び不具合の報告手順 36
13.2.4 治験調整医師の治験機器提供者への報告 36
13.3 その他の有害事象の報告 36
13.4 その他の治験使用機器（EX-01 エクステンションワイヤー／脳波計 EEG-1200 シリーズ）の不具合・有害事象症例報告 36
14 治験実施計画書からの逸脱の取扱い 38
15 中止基準と手順 39
15.1 治験の終了 39
15.2 治験の中止・中断 39
15.3 治験中止・終了後の被験者への対応方針 39
15.4 個々の被験者における治験脱落・中止基準 39
15.5 治験の中止 40
16 統計解析 41
16.1 目標症例数の設定 41
16.2 データの取扱い基準 41
16.3 解析対象集団 41
16.3.1 安全性解析対象（Safety Analysis Set：SAF） 41

16.3.2 有効性解析対象 41
16.4 被験者背景の解析 41
16.5 主要評価項目の解析 41
16.6 副次評価項目の解析 42
16.6.1 有効性副次評価項目 42
16.6.2 安全性副次評価項目 42
16.7 中間解析 42
17 治験実施計画書の遵守及び改訂 43
17.1 治験実施計画書の遵守及び逸脱又は変更 43
17.2 治験実施計画書の改訂 43
18 治験の品質管理及び品質保証 44
18.1 品質管理 44
18.1.1 原資料の直接閲覧 44
18.1.2 原資料の特定 44
18.1.3 データマネジメント 44
18.1.4 モニタリング 44
18.2 品質保証 44
19 倫理 45
19.1 治験の倫理的実施 45
19.2 被験者の秘密保全 45
19.3 被験者・代諾者への説明と同意 45
19.3.1 同意文書及びその他の説明文書 45
19.3.2 アセント説明文書およびその他の説明文書 46
19.3.3 説明文書およびアセント文書の内容 46
19.3.4 同意およびアセント取得の時期と方法 46
19.3.5 同意に関する留意事項 47
19.3.6 同意およびアセント説明文書の改訂 47
19.4 治験審査委員会 47
19.5 中央判定委員会 47
19.6 安全性評価委員会 47
19.7 利益相反 48
19.7.1 研究資金の提供 48
19.7.2 被験機器の提供 48
19.7.3 治験取得データの提供 49
20 治験に関する記録及びその保存 50
20.1 症例報告書 50
20.1.1 本治験で用いる症例報告書の様式 50
20.2 記録の保存 50
20.2.1 治験責任医師 50
20.2.2 実施医療機関 50
21 被験者の健康被害補償及び治験参加に伴う負担軽減 51
21.1 健康被害補償 51
21.2 被験者の治験参加に伴う負担の軽減 51
22 公表に関する取決め 51
23 治験実施予定期間 51
24 参考文献 52
25 別添 53
25.1 別添 1 ILAE の分類 53

＜表目次＞

表 7-1 EP-01 付属品一覧 18

表 11-1 中央判定委員会評価事項..28
表 11-2 安全性委員会評価事項..28
表 11-3　検査・観察項目及びスケジュール..30
表 19-1 安全性委員会評価事項..48

＜図目次＞

図 9-1　治験のアウトライン..22

3 略号及び用語の定義一覧

略号／用語	英文	和文
CBCT	Cone Beam Computed Tomography	コーンビームコンピュータ断層撮影
CT	Computed Tomography Scan	コンピュータ断層撮影
JIS	Japanese Industrial Standards	日本産業規格
MEG	Magneto Encephalo Graphy	脳磁図記録
MRI	Magnetic Resonance Imaging	磁気共鳴画像診断
MRV	Magnetic Resonance venography	磁気共鳴静脈撮像検査
PET	Positron Emission Tomography	陽電子放出断層撮影
SDE	Subdural Electrodes	硬膜下電極
SEEG	Stereo-Electroencephalography	定位的頭蓋内脳波
IMZ SPECT	^{123}I-iomazenil single photon emission computed tomography	イオマゼニル単一光子放射型コンピュータ断層撮影

4 開発の経緯

4.1 開発の背景

血管内脳波測定デバイス EP-01 の対象疾患は難治性てんかん(※1)である。EP-01 は脳波を検出するために脳静脈内に挿入され、難治性てんかん患者に施行される焦点切除術に重要な焦点側方性を診断することが可能な単回使用医療機器である。

※1 難治性てんかんの定義

難治性てんかんとは、医学的な意味において、主な抗てんかん薬単剤あるいは 2〜3 種類以上の多剤併用で、かつ十分量で、2 年以上治療しても、発作が 1 年以上抑制されず日常生活に支障をきたす状態と定義される。ただし、難治性てんかんの定義は絶対的なものではなく、使用される目的や場面によって異なり、例えば社会的難治てんかんとしては、成人においては道路交通法に準拠すれば「適切な薬物治療によっても 2 年以上の発作抑制を得られない場合」を言う [1)]。

てんかん患者は本邦に 100 万人程度存在する [2)] と推測されるが、そのうち 20%程度は薬物治療が奏功しない難治性てんかんである [1)]。難治性てんかんでは発作症状の緩和を抗てんかん薬投与にて行うことができないため、日常生活に影響を及ぼす発作症状を持つ患者の場合、外科的なてんかん焦点部位の切除が検討される。現在では、まず MRI・CT・SPECT および頭皮電極などを用いて焦点の同定を行う [3)] が、これらでは焦点の同定が困難な場合が多く、焦点同定の確定診断には、開頭術等による頭蓋内電極留置術が用いられる [3) 4)]。てんかん焦点が同定された後に、焦点部位を外科的に切除することで難治性てんかんを治療する。

切除範囲の決定に際しては、切除すべき領域と切除してはならない領域を鑑別することが重要であるため、頭蓋内電極による脳波からの焦点部位診断が重要である。頭蓋内電極の目的には、①てんかん原性領域の推定と②機能マッピングがあり [4)]、これらの頭蓋内電極の特性を活かして、焦点切除範囲を適切に医師が判断する。また、頭蓋内電極には「硬膜下電極(SDE)」と「深部電極による定位的頭蓋内脳波(SEEG)」の 2 種類が存在する [3) 4)]。SDE は脳表を「面として」カバーするもので開頭によって硬膜下に留置される。一方で SEEG は定位的手法により脳深部に至るまで電極を刺入し、「点として」脳波を記録する。SDE は機能マッピングが容易に可能であるが、SEEG と比較して高侵襲である。このことから、SEEG は SDE の留置が困難な再手術例やてんかん焦点の側方性を決定するに両側半球への電極留置が望ましい例などに適用しやすい [3) 4)]。これらの頭蓋内電極はてんかん焦点や切除範囲を決定するためのゴールデンスタンダードとされており [1)]、てんかん焦点切除術における切除範囲を決定するための有用な手法である。

てんかん焦点切除術の中でも、側頭葉てんかんに対する海馬・扁桃核切除術は高率に軽快することが知られており、90%以上が奏功する [7) 11) 12)]。前述の通り、切除術を実施するにあたって術前の焦点診断は極めて重要であり、頭蓋内脳波記録は発作開始時の発作波の先行起始部位が焦点として確度の高い焦点診断法である [9)]。側方性診断においては SEEG が好適であるが、本邦において SEEG に使用する専用細径電極やアンカーボルトが薬事未承認であり [5) 6)]、臨床的な運用に遅れが生じているため、本邦において側方性診断のための SDE が行われることもあるが、一方で頭蓋内電極は侵襲的であるため回避する努力も続けられており [7)]、焦点部位が不明で開頭する領域があまりにも大きい症例の場合、施行することが困難である。

また、頭皮電極で得られた脳波から得られた側方性診断結果と頭蓋内電極から得られた側方性診断結果が異なることがあることは知られており [10)]、より脳実質に近い部位で取得される脳波を用いて側方性の確定診断を行うことは非常に重要である。

4.2 デバイスの臨床的位置づけ

EP-01 は血管内からアプローチし、脳静脈内から脳波を計測するため、頭蓋内電極と比較して極めて低侵襲に脳波取得が可能となることを目指して、開発を実施している。現時点では単極であるため、頭蓋内電極の 2 つの目的のうちのひとつ、「機能マッピング」を実施することは現実的ではないが、もう一方の「てんかん原性領域の側方性診断」は可能であろうと期待される。側方性の診断が特に効果を発揮する症例が側頭葉てんかんである。頭蓋内電極が側方性の診断を行

うためにこれまでにも留置されており、より低侵襲といわれる SEEG の好適とされている 4)。1960 年代から SEEG による側方性診断が行われており 9)、少ない電極数であったとしても側方性の診断が可能なことから、EP-01 でも側方性の診断は可能と考えている。また、過去に本邦で実施された脳動脈にワイヤー状血管内脳波電極を留置した症例報告 13)において、側頭葉てんかん症例に対して頭蓋内電極と血管内脳波電極を同時に留置して、両者から同様のてんかん発作棘波を取得することが可能であった。この際の血管内脳波電極は 1 本（電極数 1 個）のみであった。さらに、本治験の研究責任医師らが実施した EP-01 の特定臨床研究によるヒトでの急性期有効性確認において、脳波取得が可能であることを確認しており、てんかん発作棘波を頭蓋内電極と同様に検出し、側方性の診断を実施できる蓋然性は高いと判断している。

EP-01 の有無による治療アルゴリズムの予想される変化について、現在のてんかん治療フロー 3)を参考にして下記に示す。

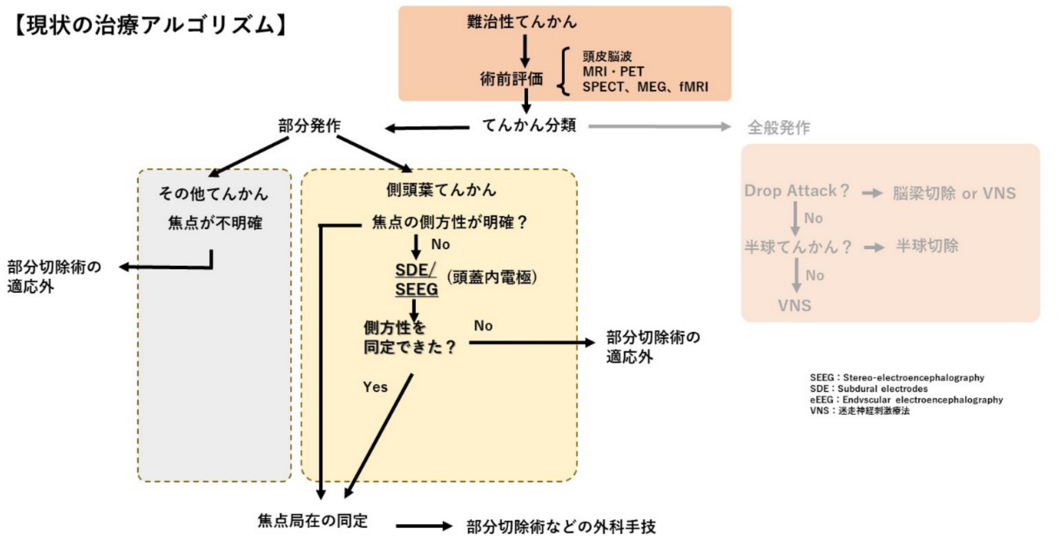

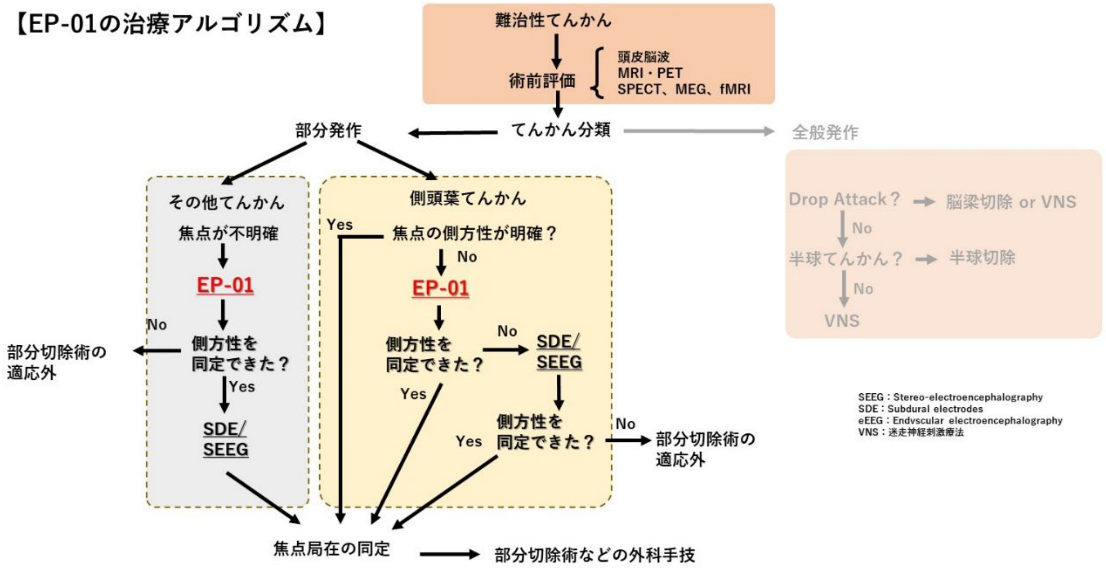

難治性てんかんのうち、焦点の部分切除術が検討されるものは「部分発作」と呼ばれる病態であり、さらに部分発作は、その焦点の位置によって「側頭葉てんかん」と「その他のてんかん」の 2 種類に分けられる。

「側頭葉てんかん」は焦点の側方性が診断されれば、切除範囲が予め術式として定められて おり 14)、側方性の診断をもって部分切除術を実施可能である。EP-01 を用いて、側頭葉てんかんの焦点側方性が診断できれば、頭蓋内電極の置き換えとなり得る。側方性の診断に至らない場合、従来通りの頭蓋内電極による側方性診断も可能である。

一方で、「その他のてんかん（前頭葉てんかんや後頭葉てんかん頭頂葉てんかん、島回てんかん）」では焦点の場所を何らかの方法で特定して、その部位を切除する必要がある。（側頭葉てんかんのように予め切除範囲が決められているわけではない）。従って、現状の頭蓋内電極を用いて焦点を特定しようとすると、極めて大規模な開頭が必要となったり、多くの本数の SEEG を実施したりする必要がある。これは非常に侵襲が高く、実臨床において実施することが躊躇されることが多い。そこで、EP-01 を用いて焦点の側方性が診断されれば、当該半球のみに頭蓋内電極を留置すればよく、頭蓋内電極留置術に対する侵襲度を低下できる。難治性てんかんの部分切除術が困難であった症例に対して、本品の使用により部分切除術に至ることが可能となり、難治性てんかんの根治に繋がると期待される。

4.3 非臨床試験成績

被験機器が臨床仕様に適した特性を有する事の確認のため、以下の試験を実施し、全ての項目基準について適合を確認している。詳細は治験機器概要書参照すること。

・物理的・化学的特性
・電気的安全性及び電磁両立性
・生物学的安全性
・安定性
・in-vitro 性能試験（使用模擬試験）
・in-vivo 性能試験（動物試験）
・その他

4.4 臨床試験成績

被験機器は「血管内脳波測定デバイスに関する初期臨床検討（以下、「初期臨床検討」）」（jRCT：jRCTs032220543）において、急性期での有効性安全性を評価した。初期臨床検討では、5 症例に対して、留置時間を 1 時間として被験機器を患者に一時留置し、頭皮電極との脳波比較ならびに 1 週間後までの有害事象発生を確認した。5 症例すべてが終了し、いずれの症例においても取得された信号が脳波であることが確認されている。さらに、術中ならびに術後 1 週間後までにおいても機器や手技を起因とする有害事象および不具合の発生は確認されておらず、安全性への懸念は現時点で認められていない。

4.5 被験者に対する既知および可能性のある危険と利益

4.5.1 被験機器 EP-01 を使用する際のリスク

EP-01 のリスクマネジメント活動は、ISO 14971 に基づき実施されている。
EP-01 の使用により以下のような不具合及び有害事象等が起こり得るが、これらに限定されるものではない。

1) 不具合
・デバイスの破損、変形、離断
・脳波測定が不可
・目的部位まで送達できない
・腐食

2) 有害事象
・出血
・血管解離
・内膜剥離
・血管攣縮
・血管穿孔
・血栓症（肺塞栓を含む）
・被験機器の遺残
・被験機器の損傷

・抜去困難
・感染症
・金属アレルギー反応
・造影剤アレルギー反応
・穿刺部血腫
・仮性動脈瘤
・脳梗塞
・腎機能障害
・てんかん発作
・不穏
・発熱

4.5.2 被験者の治験参加に関連するリスク

治験機器の挿入の際や抜去する際に行う X 線検査により放射線被曝を伴い皮膚障害や目の障害のリスクが考えられるが、治験機器の留置によって使用する放射線被曝時間は通常診療で行っている血管内検査と同程度であるため、通常臨床で用いられる検査と同等のリスクと考えられる。

WADA テストと同タイミングで治験機器の挿入を行う場合は、麻酔薬を追加で使用する事となる。麻酔薬のリスクとして、吐き気や嘔吐、頭痛、寒気・発熱などが想定される。また、WADA テスト時は通常診療としても造影剤を使用した X 線検査を行うが、本治験に参加することにより造影剤の使用量の増加、放射線被曝時間の延長が予想される。しかし、本治験で考えうる造影剤の使用量や放射線被曝時間は通常診療で行われる血管内検査と同程度であり、それと同様のリスクといえる。

頭蓋内電極の留置と同タイミングで治験機器の挿入を行う場合は、造影剤を使用した X 線検査を追加で行う。そのため、頭蓋内電極の留置に伴うリスクに加え、血管内検査時と同様の造影剤及び放射線被曝に伴うリスクがある。なお、頭蓋内電極留置時は通常診療としても麻酔薬が使用されるため、麻酔薬に関するリスクは通常診療と同程度である。

上記より 4.5.1 に記載の被験機器の使用に関するリスクに加えて治験参加に伴うリスクは一般臨床で行われる検査によるものと同程度と考えられる。

4.5.3 被験者に対する既知および可能性のある危険と利益の要約

治験参加によって被験者本人に期待される直接的な利益はないが、4.開発の背景に記載の通り、この治験で得られる結果により、被験機器を用いた血管内脳波検査が頭蓋内電極による脳波検査に匹敵し、将来的に難治性てんかん患者において、頭蓋内電極による脳波検査を実施せずに、てんかんの外科的治療を行うことができる可能性がある。

5 本治験の目的

血管内脳波測定デバイス EP-01 を用いた難治性てんかん患者におけるてんかん焦点の側方性診断に対する有効性について検討する。また、EP-01 を使用した際の安全性について評価する。

6 対象被験者の選択

6.1 対象被験者

頭蓋内電極留置術を施行する難治性てんかん患者

【設定根拠】

てんかん焦点の側方性の診断能を頭蓋内電極と比較するため、焦点の部分切除術を実施するための頭蓋内電極留置を行う予定の被験者に設定した。

6.2 選択基準

登録時に、以下の基準すべてを満たす患者を選択する。

(1) 難治性てんかん患者のうち、側頭葉てんかん、前頭葉てんかん、頭頂葉てんかん、後頭葉てんかん、島回てんかん患者
(2) 頭蓋内電極を留置する患者
(3) 海綿静脈洞・横静脈洞・上矢状静脈洞・下錐体静脈洞などてんかん焦点に近い血管に対し、左右の頸静脈から EP-01 を脳内に誘導できる血管解剖を有する患者［EP-01 本体の留置血管は医師の判断とする。ただし脳静脈の両側の対称位置へ誘導出来る事が確認できている事は必須とする］
(4) 15 歳以上 70 歳未満（同意取得時）

【設定根拠】
（1）（2）：対象疾患として設定した。
（3）：倫理的に設定した。
（4）：頭蓋内電極は小児でも適応があるが、安全性を考慮して設定した。

6.3 除外基準

登録時に以下の基準いずれかに該当する患者を除外する。

(1) 全般発作のみを有するてんかん患者
(2) 内頸静脈に閉塞・血栓がある患者
(3) 出血素因および凝固異常がある患者
(4) 手術時に安静を保つことが困難である患者
(5) 重篤な造影剤アレルギーを有する患者
(6) 金属アレルギーを有する患者
(7) 妊娠あるいは妊娠の可能性のある患者
(8) 他の治験に参加している患者
(9) 責任医師が又は分担医師が本試験への参加を不適切と判断した患者

【設定根拠】
（1）：対象疾患をより明確化するため設定した。
（2）～（8）：安全性への配慮のため設定した。
（9）：安全性および有効性評価への影響への配慮のため設定した。

7　治験使用機器

治験使用機器として被験機器 EP-01（EP-01 本体および EP-01 付属品）および EP-01 の有効性・安全性を評価するために使用する機器である EX-01 エクステンションワイヤー/株式会社 Epsilon Medical および脳波計 EEG1200 シリーズ　ニューロファックス/日本光電工業株式会社を用いる。

7.1　被験機器

本治験における被験機器を使用した治験手技については、治験調整医師もしくは治験調整医師が指名した医師による EP-01 本体および EP-01 付属品の使用方法に関するトレーニングを受けた各実施医療機関の治験責任医師および治験分担医師に限る。なお、被験機器の詳細に関しては治験機器概要書を参照すること。

7.1.1　被験機器の名称又は識別記号

被験機器の名称　：血管内脳波測定デバイス EP-01
被験機器識別番号：EP-01
クラス分類：IV

EP-01 は EP-01 本体および EP-01 付属品（接続コネクターとシースセット、ワイヤー固定具、インサーター、ロングシース、ワイヤー用ウィング、ディスペンサー）より構成される。

EP-01 本体および接続コネクターは症例により最大 6 本、シースセットは 2 個、ワイヤー固定具、インサーター、ロングシース、ワイヤー用ウィングは状況により最大 2 個使用する。EP-01 本体および EP-01 付属品はそれぞれ個別包装される。

7.1.2　EP-01 本体

血管内脳波測定デバイス本体は、電極部がリードワイヤー部の先端に取り付けられ、経皮経血管的に脳波を測定する医療機器である。リードワイヤーは表面が絶縁され、手元側において脳波測定装置と接続可能な構造を有する。

電極部で脳波を取得し、リードワイヤーを介して脳波信号を導通させる。リードワイヤー後端部には接続コネクターと接続されるために、金属露出部分があり、接続コネクターと接続される。接続コネクターによって電極部で得られた脳波信号が脳波測定装置に入力される。

EP-01 本体は手元側に識別マーカーが敷設されるが、先端の電極構造などに識別マーカーごとの差異はなく、すべて同一である。識別マーカーは体内に挿入された EP-01 本体の識別を容易にするために、6 種類が設定されている

7.1.3　EP-01 付属品

EP-01 付属品は接続コネクターとシースセット、ワイヤー固定具、インサーター、ロングシース、ワイヤー用ウィングからなる（表 7-1　EP-01 付属品一覧）。

表 7-1　EP-01 付属品一覧

付属品名称	用途
接続コネクター	EP-01 本体と脳波測定装置を接続する
シースセット	EP-01 本体を体内に挿入する際に使用し、EP-01 留置中においてもそのまま留置される。
ワイヤー固定具	患者から出た EP-01 本体のワイヤーを固定する。
インサーター	EP-01 本体の先端電極部をマイクロカテーテル内に挿入しやすくするために使用する。
ロングシース	EP-01 本体を体内に留置する際、シースセ

	ットのシースを延長する為に使用する。
ワイヤー用ウィング	EP-01 本体を体内に留置中にリードワイヤーを固定するために使用する。
ディスペンサー	輸送時、EP-01 本体を収納し、保護する包装保護材。

7.1.4 被験機器包装及び表示

被験機器および EP-01 付属品のラベルは以下を記載する。

治験用である旨
自ら治験を実施する者の氏名及び住所
原材料名又は識別記号
製造番号又は製造記号
貯蔵方法、有効期間等

7.2 被験機器の交付・管理・出庫手順

治験機器提供者は自ら治験を実施するものが本治験の治験計画届を提出したのち、被験機器を各実施医療機関へ交付する。各実施医療機関の治験機器管理者（または治験機器管理補助者）は自ら治験を実施する者が作成する「治験機器の管理に関する手順書」に従い EP-01 を管理・出庫する。

7.2.1 被験機器取扱い上の注意

被験機器の保管条件及び使用期限は、安定性試験で安定性が確認できた期間（製造後 3 年）とし、水濡れに注意し、高温、多湿、直射日光を避けて保管する事とする。

7.2.2 被験機器の廃棄

治験実施医療機関は、使用済みの被験機器は各実施医療機関の手順に従い廃棄する。なお、詳細は治験機器の管理に関する手順書を確認すること。また、開封済み未使用機器は実施医療機関の規定に従い廃棄し、廃棄記録を作成する。未使用（未開封）の被験機器は治験終了時に全ての未使用品を治験機器提供者に返却し、その際に返却記録を作成する。

7.3 その他の治験使用機器

被験機器 EP-01 の有効性・安全性を評価するために使用する機器である EX-01 エクステンションワイヤー/株式会社 Epsilon Medical および脳波計/日本光電工業株式会社を用いる。本治験により生じた EX-01 エクステンションワイヤーおよび脳波計　EEG1200 シリーズに関連する安全性情報は、「13.4　その他の治験使用機器（EX-01 エクステンションワイヤー／脳波計　EEG-1200 シリーズ）の不具合・有害事象症例報告」に従い適切に対応する。

7.3.1 EX-01 エクステンションワイヤー／株式会社 Epsilon Medical

EP-01 本体からマイクロカテーテルを抜去する際に EP-01 の全長を一時的に延長するため症例状況に応じて必要時用いる未承認機器。EX-01 エクステンションワイヤー（以下、EX-01）は個別に 1 個単位で包装される。

7.3.1.1 EX-01 の交付・管理・出庫手順

被験機器と同様に自ら治験を実施するものが本治験の治験計画届を提出したのち、治験機器提供者より交付される。各実施医療機関の治験機器管理者（または治験機器管理補助者）は自ら治験を実施する者が作成する「治験機器の管理に関する手順書」に従いその他の治験使用機器を管理・出庫する。

7.3.1.2 EX-01 の取り扱い上の注意

EX-01 の保管条件は水濡れに注意し、高温、多湿、直射日光を避けて保管する事とする。

7.3.1.3 EX-01 の廃棄

治験実施医療機関は、使用済みの EX-01 は各実施医療機関の手順に従い廃棄する。なお、詳細は治験機器の管理に関する手順書を確認すること。また、開封済み未使用機器は実施医療機関の規定に従い廃棄し、廃棄記録を作成する。未使用（未開封）の EX-01 は治験終了時に全ての未使用品を治験機器提供者に返却し、その際に返却記録を作成する。

7.3.2 脳波計 EEG1200 シリーズ ニューロファックス／日本光電工業株式会社

EP-01 と接続して脳波測定を行うために用いる。EEG1200 シリーズの脳波計であり、型名は問わない（ただし型名 EEG-1250 は除く）。これらは、医療機関にて採用され使用されている機器を用い、本治験としての管理を必要としないが、各治験実施医療機関の規定に従い精度管理を行うこと。治験責任医師又は治験分担医師は、機器の精度管理記録情報等により適切に管理されていることを確認する。

製造販売認証番号：218AHBZX00013000
製造販売業者：日本光電株式会社

8 被験者の登録

EP-01 により介入を行う被験者数を 37 例と設定している。

8.1 登録手順

治験責任医師又は治験分担医師は、選択基準を満たし、除外基準に抵触しないことをスクリーニング期間に確認したのちに登録する。登録は EDC に適格条件を記載し、登録する。

選択基準又は除外基準を満たさず登録されない被験者で、不適格と判定された以降の本治験の検査の実施は不要とする。

8.2 治験に組み入れられた被験者の定義

本治験に登録され、EP-01 による脳波検査開始した症例を「EP-01 脳波検査開始例」とし、EP-01 脳波検査開始例の内、治験検査期間を完了した症例を「治験検査期間完了例」、なんらかの理由により脳波検査期間中または後観察期間中に中止した症例を「中止例」とする。また、部分切除術に至り、切除術から 1 年後の評価を完了した症例を「てんかん発作転帰評価完了例」、治験検査期間完了後 1 年目の評価前に中止した症例を「てんかん発作転帰評価期間中止症例」と定義する。

なお、同意取得後に登録に至らなかった症例を「不適格例」といい、登録後、なんらかの理由により EP-01 による脳波検査開始に至らなかった症例を「脱落例」と定義する。

9 治験の方法

9.1 治験デザイン

本試験は、単群多施設共同オープン試験（検証的臨床試験）である。

治験のアウトラインを図 9-1 に示す。

本治験は最長 30 日のスクリーニング期間、最大 2 週間の治験検査期間、1 週間の後観察期間で構成される。てんかん焦点切除術に至った症例は、外科手術から 1 年後にてんかん発作転帰評価を行う。

なお、後観察期間終了までのデータを一次解析対象とし、被験機器の評価とする。外科手術より 1 年後のてんかん発作転帰評価データを 2 次解析対象とする。

スクリーニング期間	治験検査期間	後観察期間	てんかん発作転帰評価期間
（30 日～1 日前）	（最大 2 週間）	（1 週間）	外科手術から 1 年

同意取得　　被験機器留置期間　　被験機器評価期間終了　　てんかん発作転帰評価期間終了

図 9-1 治験のアウトライン

【設定根拠】

スクリーニング期間（30 日～1 日間）は本治験の定められる検査完了可能期間として設定した。治験検査期間（最大 2 週間）は被験機器としての評価を行う上で、てんかん発作の発生し得る期間であり、また、被験機器の非臨床試験において安全性が確認されている期間とした。後観察期間は、頭蓋内電極および EP-01 留置期間中に想定される有害事象の発現期間とした。

9.2 診断

臨床所見および MRI 等による画像所見および頭皮電極の結果をもとにてんかん部分発作であると診断され、かつ海綿静脈洞・横静脈洞・上矢状静脈洞・下錐体静脈洞などの、てんかん焦点に近い血管へ EP-01 を誘導できる血管解剖を有し、EP-01 及び頭蓋内電極の留置が可能であると医師が判断した患者を対象とする。なお、これら EP-01 の留置判断に伴う検査は、被験者の負担を考慮し、同意取得前 90 日前までに取得されたデータをもとに判断することも可とする。

9.3 治験使用機器の用法および留置期間

本治験における機器の留置は単回とし、最大 2 週間（アロワンス+3 日）を留置期間とする。

9.3.1 被験機器の留置方法

EP-01 付属品であるシースを左右の頸静脈よりシース先端を頭側に向けて留置する。当該シースからマイクロカテーテルを目的の脳静脈血管まで挿入させる。目的血管へ到達済みのマイクロカテーテルを通じて EP-01 本体を挿入し、目的部位に送達させる。EP-01 本体をマイクロカテーテル内に導入する際の補助として、必要に応じてインサーターを使用する。EP-01 本体が目的部位に送達できたことを確認したのちに、マイクロカテーテルを抜去する。この際、必要に応じてエクステンションワイヤーを使用する。マイクロカテーテルを抜去したのちに、電極部で取得された脳波はリードワイヤー部を介して、脳波測定装置に入力される。脳波測定装置に脳波が表示された事を確認し、EP-01 本体の留置完了とする。留置を終了する際は、Tuohy Borst バルブやシリコンチューブを使用して、適切に固定する。最大 6 本を留置後、被験者頸部から出ているワイヤーを固定し、処置完了とする。

EP-01 本体を留置する血管の決定は、海綿静脈洞・横静脈洞・上矢状静脈洞・下錐体静脈洞などの、てんかん焦点に近い血管とするが、症例状況に応じて治験責任医師または治験分担医師（以下、治験責任医師等）の判断とする。ただし脳静脈の両側の対称位置へ誘導出来る事が確認できている事は必須とする。詳細手順は「治験手技に関する手順書」に従って行うこと。

9.3.2 頭蓋内電極の留置方法

原則、EP-01 本体の留置後に頭蓋内電極を留置する。各治験実施医療機関で通常臨床上行われている留置方法に従う。留置箇所の選択についても同様とする。なお、EP-01 留置を先に行う場合、10.2.2 併用禁止および制限薬及び療法に記載の通り、EP-01 留置期間中の外科的処置に用いる電気メスはモノポーラー使用不可であることに留意する。

9.3.3 被験機器留置のタイミング

EP-01 本体の留置は原則として WADA テスト実施時または頭蓋内電極留置時とする。EP-01 本体を頭蓋内電極留置と別日で実施する場合、頭蓋内電極の留置と EP-01 本体の留置間隔は 3 日以内とする。なお、10.2.2 併用禁止および制限薬及び療法に記載のとおり、MRI による撮像禁忌であることから、EP 01 留置後に頭蓋内電極留置する場合は MRI ガイド下でのフレームの設置は不可であることに留意する。

9.3.4 被験機器の留置期間中

EP-01 本体留置期間中はシースの血栓予防のため、原則としてシースに取り付けている Tuohy Borst バルブのサイドポートよりヘパリン加生理食塩液の持続灌流を行う。ヘパリン加生理食塩液の持続灌流が被験者の状況より困難と治験責任医師等が判断する場合、最低限 1 日 1 回フラッシュおよびロックを行う（フラッシュおよびロックはヘパリン加生理食塩液を用いることを推奨する)。

EP-01 本体留置期間中、X 線検査にて先端電極位置を確認する。X 線検査にて先端電極位置が著しくずれていることが確認された場合は、被験者の安全性を確保する事を最優先とし、治験責任医師等の判断をもとに、EP-01 本体の抜去や経過観察等の対処を検討し対応する。また、有害事象の有無の確認を CT 検査にて実施する。有害事象が確認された場合は治験責任医師等の判断をもとに適切に対処を行う。脳波記録方法等の詳細手順は「治験手技に関する手順書」に従って行うこと。

9.3.5 被験機器の抜去

留置中にてんかん発作の発生が確認された後、治験責任医師等の判断のもと、EP-01 本体を抜去する。発作が確認出来なかった場合、最大 2 週間（アロワンス+3 日）まで留置したのち、抜去する。留置中にてんかん発作が生じなかった、あるいは、なんらかの不具合等により脳波信号を感知できなかった場合は中止例とする。

9.3.6 頭蓋内電極の抜去

頭蓋内電極の抜去時期および方法は、臨床判断に従う。被験機器の抜去と同タイミング、またはそれ以降に抜去することも可とする。

10 被験者の管理

10.1 前治療薬剤及び療法

頭蓋内電極留置術に合わせ、必要に応じて医師判断にて通常臨床で実施される断薬および減薬を行う。

原疾患に対する登録時前の治療歴（外科的手術歴を含む）については、前治療歴として症例報告書に記載する。

10.2 併用薬及び療法

治験責任医師等の判断で、被験者の利益のために必要とされる治療は実施してもよい。

登録から後観察期間終了までに使用した薬剤（処方薬）及び療法について併用薬・併用療法として症例報告書に記載する。

なお、頭蓋内電極留置（追加留置含む）および抜去のために使用した併用薬および併用療法については収集対象外とする。また、てんかん焦点切除術が後観察期終了時までに行われる場合、それらに使用した併用薬および併用療法についても収集対象外とする。ただし、抗血栓療法については 10.2.1 に記載の通りとする。

10.2.1 抗血栓療法

抗血栓療法に関しては、同意取得前 30 日から後観察期間終了まで抗血栓療法として症例報告書に記載する。

10.2.2 併用禁止および制限薬及び療法

EP-01 留置中の MRI 撮像は禁忌とする。また、EP-01 留置期間中の外科的処置に用いる電気メスにおいて、電流の影響を最小限に抑えるため、対極板を用いるモノポーラは使用禁止とする。

上記の他、本治験期間を通して、通常臨床において頭蓋内電極留置術等で実施される制限に加えての併用禁止および制限薬・療法の規定は設けない。

10.2.3 併用注意薬および療法

本治験期間を通して、併用注意薬および療法の規定は設けない。

11 検査・観察項目及びスケジュール

本治験における時期の規定は以下のとおりとし、規定の来院日に下記の観察・検査を実施する。機器の留置日を起点（0 日）とし、各検査スケジュールを設定する。

11.1 観察項目

11.1.1 被験者背景調査

同意取得後、被験機器の留置より 30 日～1 日前までに、選択基準を満たし、除外基準に抵触しないことを確認し、以下項目を登録時点で調査する。被験者識別コード及び被験者背景情報は症例報告書に記載する。
観察項目：性別、年齢、血圧、脈拍、現病歴（てんかん分類、発作型分類、期間、年月、病因)、合併症、既往歴、前治療歴、喫煙歴、飲酒歴
合併症：同意取得時点で合併している疾患とする。
既往歴：同意取得以前に罹患した疾患とし、同意取得日の 90 日前までの情報を調査する。ただし周産期異常、熱性けいれん、頭部外傷、精神疾患、悪性腫瘍等、治験責任医師又は治験分担医師が重要と判断する既往については期間を問わない。
前治療歴：原疾患に対する登録時前の治療歴（外科的手術歴を含む）について調査する

11.1.2 身長・体重

体重および身長を測定し、診療記録及び症例報告書に記載する。
実施時期：同意取得後、被験機器の留置より 30 日～1 日前までに実施するが、同意取得前 90 日以内に実施していれば、検査不要とし、同意取得前のデータを使用する。
検査項目：身長・体重

11.1.3 頸部エコー

頸部エコーを実施し、診療記録及び症例報告書に診断所見を記載する。
実施時期：同意取得後、被験機器の留置より 30 日～1 日前までに実施するが、同意取得前 90 日以内に実施している場合は検査不要とし、同意取得前データを使用する。
観察項目：臨床的に問題となる総頸動脈・内頸静脈の閉塞・血栓の有無

11.1.4 血液検査

血液検査を実施し、その結果を診療記録及び症例報告書に記載する。
実施時期：同意取得後、被験機器の留置より 30 日～1 日前までに実施するが、同意取得前 90 日以内に実施している場合は検査不要とし、同意取得前データを使用する。
検査項目：血小板数、APTT、PT-INR

11.1.5 非侵襲的所見

11.1.5.1 てんかん発作臨床所見

てんかん発作に関する臨床所見を症例報告書に記載する。なお、被験者状態に応じ、治験責任医師等の判断によりてんかん焦点側方性診断に必要なもののみ調査する。
実施時期：同意取得後、被験機器の留置より 30 日～1 日前までに実施するが、同意取得前 1 年以内に実施している場合は再調査不要とし、同意取得前のデータを使用する。
観察項目：てんかんガイドラインに定めるてんかん問診内容、神経心理検査

てんかんガイドラインに定めるてんかん問診内容：発作頻度、発作状況・誘因、発作前及び発作中の症状、症状の持続、発作に引き続く症状、外傷・咬舌・尿失禁の有無、発作後の頭痛と筋肉痛、複数回の発作のある患者では初発年齢、発作及び発作型の変化・推移、最終発作、発作と覚醒・睡眠との関係、発作後の行動、状態の詳細
神経心理検査：左右の前頭葉・側頭葉・後頭葉・頭頂葉の機能低下の有無

11.1.5.2 MRI 等による画像所見

てんかん焦点の側方性診断のための MRI 等（通常診療にて実施している場合は CT、FDG-PET、IMZ-SPECT、MEG 等含む）画像データを取得する。取得したデータについては中央判定委員会に関する手順書に従い、中央判定委員会へ提出する。

実施時期：原則、同意取得前 1 年以内にてんかん焦点の側方性診断のために実施した検査を収集する。1 年以内に撮像していない場合においても、治験目的の再撮像は不要とする。再撮像を行わない場合は、直近の画像所見を中央判定委員会へ提出する。

11.1.5.3 頭皮電極検査

てんかん焦点の側方性診断のための頭皮電極による検査データを取得する。取得したデータについては中央判定委員会に関する手順書に従い、中央判定委員会へ提出する。

実施時期：原則、同意取得前 1 年以内にてんかん焦点の側方性診断のために実施した検査を収集する。1 年以内に検査していない場合においても、治験目的の再検査は不要とする。再検査を行わない場合は、直近の脳波所見を中央判定委員会へ提出する。

11.1.6 MRI 検査

海綿静脈洞・横静脈洞・上矢状静脈洞・下錐体静脈洞などの、てんかん焦点に近い血管へ EP-01 本体を誘導できる血管解剖を有することを確認するため単純 3D-T1WI および、MRV または造影 3D-T1WI を実施し、診療記録及び症例報告書に診断所見を記載する。

実施時期:同意取得前 90 日以内に実施していれば､再測定不要とし､同意取得前のデータを収集する。

11.1.7 マイクロカテーテル留置場所の確認

EP-01 本体の留置手技途中におけるマイクロカテーテルの留置場所を確認する。

実施時期：EP-01 留置手技途中

観察項目：マイクロカテーテルの留置血管名

11.1.8 EP-01 留置場所の確認

EP-01 本体の先端電極の留置場所を X 線検査より確認する。

実施時期：留置直後、留置 1 日後、留置 7 日後、抜去直前（ただし、7 日以前に抜去する際は留置 7 日後の実施は省略する）

観察項目：各時期における EP-01 留置血管名

11.1.9 CT 検査

EP-01 本体留置箇所からの有害事象の有無を CT 検査により確認する。スライス厚 1 mm 以下および 3~5mm の 3D データを取得する。取得したデータについては安全性評価委員会に関する手順書に従い、安全性評価委員会へ提出する。なお、通常臨床で同意取得前 90 日以内に実施している場合のみ、安全性評価委員へ提供する。実施していない場合の再撮像は不要。

実施時期： 留置直後、留置 1 日後、留置 7 日後、抜去直前（ただし、7 日以前に抜去する際は留置 7 日後の実施は省略する）、抜去 1 日後、抜去 7 日後

11.1.10 脳波検査

EP-01 および頭蓋内電極による脳波をビデオ監視下で測定する。脳波記録方法等の詳細手順は「治験手技に関する手順書」に従って行うこと。取得したデータについては中央判定委員会に関する手順書に従い、中央判定委員会へ提出する。

実施時期：機器の留置～機器の抜去（EP-01 のみまたは EP-01 と頭蓋内電極の両方が留置されている期間を含む）

観察項目：EP-01 および頭蓋内電極の留置に関する情報、非侵襲的所見に、EP-01 の脳波所見を加えた側方性診断結果と、非侵襲的所見に頭蓋内電極の脳波所見を加えた側方性診断結果

中央判定委員会への提供脳波データ：

- 開閉眼時の脳波データ

  開閉眼時の脳波データ取得タイミングは治験責任医師等の判断とする

- てんかん発作時脳波データ
  ＜対象とするてんかん発作時脳波の選択基準＞
  ・臨床症状を有する発作
  ・EP-01 脳波と頭蓋内電極脳波両方が明瞭に記録できている
  ・重積中の発作ではない
  ・上記を満たす発作のうち、診断に利用した最初の発作

11.1.11 有害事象の観察

有害事象とは、EP-01（本体および付属品）またはその他の治験使用機器（EX-01 エクステンションワイヤー／脳波計　EEG1200 シリーズ　ニューロファックス）を使用して以降、治験検査期間及び後観察期間に認められたあらゆる好ましくない徴候・症状又は病気（臨床検査値の異常変動を含む）のことであり、治験使用機器との因果関係は問わない。有害事象を認めた場合、治験責任医師等は、適切に処置を行う。また、治験使用機器との因果関係の有無に係わらず、原則として正常化又は有害事象として捉えないレベルに回復するまで追跡調査を実施する。器質的な障害で不可逆的な有害事象が認められた場合は、症状が安定又は固定するまで追跡調査を行う。原疾患で想定される範囲内の症状は有害事象として取り扱わない。また、治験検査期間及び後観察期間にてんかん焦点切除術が行われた場合、当該処置に起因した症状は有害事象として取り扱わない。EP-01（本体および付属品）を使用して以降、治験検査期間及び後観察期間に発現した全ての有害事象について、以下の項目を診療記録及び症例報告書に記載する。

・有害事象名
・発現日
・転帰日
・転帰（回復、軽快、未回復、死亡、追跡不能、不明）
・処置（あり、なし。ありの場合はその内容）
・程度（軽度：無処置で治験継続可能な状態、中等度：何らかの処置が必要であるが治験継続可能な状態、重度：治験を中止あるいは中止すべき状態）
・重篤性（重篤、非重篤）
・治験使用機器（留置および抜去の医学的行為を含む）または頭蓋内電極との関連性（関連性あり、関連性なし、不明）

11.1.12 不具合の観察

「不具合」とは、治験使用機器の機能の不全、治験使用機器が人体に及ぼす副作用、破損、作動不良等広く具合のよくないことをいい、製造、交付、保管又は使用のいずれの段階によるものであるかを問わない。

治験検査期間に発現した全ての不具合について、以下の項目を診療記録及び症例報告書に記載する。

・不具合名
・治験使用製品の不具合が発生したと考えられる原因
（運搬、保管、手技、原疾患、併用薬・併用療法、その他）
・治験使用機器の不具合状況
・EP-01 本体、EP-01 付属品（接続コネクターとシースセット、ワイヤー固定具、インサーター、ロングシース、ワイヤー用ウィング、ディスペンサー）、脳波計 EEG1200 シリーズ ニューロファックス、EX-01 エクステンションワイヤーとの関連性

11.1.13 併用薬及び療法

10.2 併用薬及び療法に記載の通り、登録から後観察期間終了までに使用した薬剤（処方薬）及び療法について併用薬・併用療法として症例報告書に記載する。なお、抗血栓療法に関しては、同意取得前 30 日から後観察期間終了まで抗血栓療法として症例報告書に記載する。なお、頭蓋内電極留置（追加留置含む）および抜去のために使用した併用薬および併用療法については収集対象外とする。また、てんかん焦点切除術が後観察期終了時までに行われる場合、それらに

使用した併用薬および併用療法についても収集対象外とする。ただし、抗血栓療法については 10.2.1 に記載の通りとする。

11.1.14 ILAE の分類

術後のてんかん発作転帰評価について「術後のてんかん発作転帰評価に関する手順書」に従い、ILAE の分類[14]を用いて評価し、その結果を診療記録及び評価シートに記載する。評価結果は治験調整事務局に送付する。
実施時期（許容範囲）: 外科的治療日より 365 日後（±60 日）
観察項目 : 外科的手術実施日、外科的手術による摘出部位、評価日、ILAE の分類結果

11.1.15 中央判定委員会による評価

治験調整医師は、第 3 者による有効性評価を行う目的として、中央判定委員会を設置する。中央判定委員会は「中央判定委員会に関する手順書」に従い、各実施医療機関で取得されたデータをもとにてんかん焦点の側方性診断等を評価する。

表 11-1 中央判定委員会評価事項

有効性に関する評価目的および時期	第 3 者によるてんかん焦点の側方性診断について、バイアス評価の疑いを避けるため、第 3 者の評価を用いることが目的。評価は、てんかん焦点の側方性診断結果に関して治験責任医師等の判定終了後に実施する。<br>実施時期 : 症例のデータを各実施医療機関から中央判定委員会事務局を通じて中央判定医へ送付された後、都度実施する。

上記業務において、中央判定委員会が他の専門家の意見聴取が必要と判断した場合は、治験調整医師へ連絡の上、他の専門家にオブザーバー参加を依頼し意見を聴取する。

11.1.16 安全性評価委員会による評価

治験調整医師は、第 3 者による安全性評価を行う目的として、安全性評価委員会を設置する。安全性評価委員会は「安全性評価委員会に関する手順書」に従い、各実施医療機関で取得されたデータをもとに安全性を評価する。

表 11-2 安全性委員会評価事項

安全性に関する評価目的および時期	安全性に関する評価を行うとともに、治験継続の適否について評価することが目的。<br>(1) 定期評価<br>各実施医療機関で撮像した画像検査等より判断する有害事象*の有無、重症度および機器との関連性を評価する。<br>*画像検査等より判断する有害事象例 : 出血、正中偏移、髄液漏<br>安全性評価判定は、各実施医療機関から治験調整事務局へ CT 画像データが提出された後、治験実施計画書に定めた定期開催時期に実施する。<br>・定期第 1 回目…15～20 症例（※症例数に応じて修正）の症例データが各実施医療機関から安全性評価委員会事務局へ提出された時点で開催調整を実施<br>・定期第 2 回目…全症例の症例データが各実施医療機関から安全性評価委員会事務局へ提出された時期<br><br>(2) 臨時評価<br>被験機器を起因とする重篤な有害事象が認められた等による有害事象の発現状況及び治験機器提供会社から提供された安全性情報等を基に、治験調整医師が必要と判断した場合、臨時開催として実施する。<br>各実施医療機関から報告された被験機器を起因とする重篤な有害事象に加え、以下を基に、治験調整医師が必要と判断した場合、当該治験の継続等及

	び治験実施計画書の変更等についての提言を行う。<br>・ 治験全体の継続等に関わる新たな重要な安全性情報の入手時<br>・ 当初の予測を大幅に上回る有害事象（又は副作用）又は不具合の発生時<br>・ 当初の予測より有効性が著しく高いあるいは低い状況の発生時<br>・ 安全性若しくは有効性に係る新たな重大な情報の入手時<br><br>(3) その他<br>その他治験調整医師が必要と判断した場合に開催する。

上記業務において、安全性評価委員会が他の専門家の意見聴取が必要と判断した場合は、治験調整医師へ連絡の上、他の専門家にオブザーバー参加を依頼し意見を聴取する。

11.2 検査・観察項目及びスケジュール

検査・観察項目及びスケジュールを表 11-3 検査・観察項目及びスケジュールに示す。

表 11-3　検査・観察項目及びスケジュール

項目	スクリーニング期間	被験機器評価期間 / 治験検査期間						被験機器評価期間 / 後観察期間		てんかん発作転帰評価
時期	スクリーニング		被験機器留置[10)]	被験機器留置1日後	頭蓋内電極留置	被験機器留置1週間後	被験機器抜去[11)] / 中止時[15)]	被験機器抜去1日後	被験機器抜去1週間後/中止時[15)]	外科的治療から1年後[12)]
許容範囲	被験機器留置前30日〜1日				被験機器留置±3日以内		被験機器留置から14日以内[13)]	＋3日	＋3日	±60日
同意取得	●									
登録		●								
適格性確認	●									
被験者背景調査[1)]	●									
身長・体重[2)]	●									
頸部エコー[3)]	●									
血液検査[4)]	●									
非侵襲的所見[5)]：てんかん発作臨床所見	●									
非侵襲的所見[5)]：画像所見	●									
非侵襲的所見[5)]：頭皮電極	●									
画像検査：MRI 検査[6)]	●									
画像検査：X 線検査[7)]			●	●		●	●			
画像検査：CT[8)]	○[14)]		●	●		●	●	●	●	
脳波検査[16)]			←	―	―	―	→			
有害事象			←	―	―	―	―	―	→	
不具合			←	―	―	―	→			
原疾患治療・抗血栓療法[9)]	←	―	―	―	―	―	―	―	→	
併用薬			←	―	―	―	―	―	→	
てんかん発作評価(ILAE 分類)										●

●：本治験で必須となるデータまたは観察項目　○：本治験目的での実施は不要

1) 同意取得時年齢、性別、血圧、脈拍、原病歴、合併症、既往歴、喫煙歴、飲酒歴を収集する。
2,3) 同意取得前 90 日以内に実施していれば、再測定不要とし、同意取得前のデータを収集する。
4) 同意取得前 90 日以内に実施していれば、再測定不要とし、同意取得前のデータを収集する。血小板数、APTT、PT-INR　の検査を実施する。
5) 被験者状態に応じ、治験責任医師等の判断によりてんかん焦点側方性診断に必要なもののみ調査する。
6) 単純 3D-T1WI および、MRV または造影 3D-T1WI を実施する。同意取得前 90 日以内に実施していれば、再測定不要とし、同意取得前のデータを収集する。
7) X 線検査（正面・側面）を実施する。検査のタイミングは、被験機器留置直後・被験機器留置翌日・被験機器留置 1 週間後（被験機器留置 1 週間以内にてんかん発作発現していれば不要）・被験機器抜去直前に実施する。
8) EP-01 留置直後の撮像方法としてマルチスライス CT に限らずコーンビーム CT（CBCT）での撮像でも可。CT 検査のタイミングは、被験機器留置直後・被験機器留置翌日・被験機器留置 1 週間後（被験機器留置 1 週間以内にてんかん発作発現していれば不要）・被験機器抜去直前に実施する。スライス厚 1mm 以下および 3~5mm の 3D データを安全性評価委員会へ提出。
9) 原疾患に対する治療（外科的手術歴含む）および抗血栓療法に関しては、同意取得以前 から後観察期間終了までを収集する。
10) 原則、WADA テストまたは、頭蓋内電極留置と同手術内で被験機器の留置を行う。
11) てんかん発作が発現するまで、または、最大 14 日間（＋3 日）被験機器を留置する。被験機器と頭蓋内電極抜去、外科的治療を同日にて実施することも可能。被験機器の先端位置の移動が疑われた場合は、被験機器の抜去判断のためすみやかに X 線検査を実施する。
12) てんかん切除術を行った被験者のみ実施。
13) 各実施医療機関の体制上、最大 2 週間の抜去が難しい場合は、+3 日間のアロワンスを設けることを可能とする。
14) 通常診療で被験機器留置前 90 日以内に実施している場合のみ、安全性評価委員へ提供する。実施していない場合の再撮像は不要。
15) 治験検査期間中に中止する場合、被験者の状況に応じて被験機器抜去時に定められた検査を可能な限り実施した後、被験機器の抜去を実施し、後観察期間へ移行する。後観察期間中に中止する場合、被験者の状況に応じて被験機器抜去後 1 週間後に定められた検査を可能な限り実施した後、治験終了とする。
16) EP-01 のみまたは EP-01 と頭蓋内電極の両方が留置されている期間、EP-01 および頭蓋内電極による脳波をビデオ監視下で測定する。開閉眼時の脳波データ（開閉眼時の脳波データ取得タイミングは治験責任医師等の判断とする）およびてんかん発作時脳波データを中央判定委員会へ提出する。

12 評価項目

12.1 主要評価項目

・EP-01 によるてんかん焦点の側方性の診断能：中央判定委員会による第 3 者評価

臨床所見および同意取得日から過去 1 年以内に得た画像所見、頭皮電極による脳波所見（以下、非侵襲的所見）に、EP-01 による脳波所見を加えたてんかん焦点の側方性（以下、側方性）診断結果と、非侵襲的所見に頭蓋内電極による脳波所見を加えた側方性診断結果とを比較し、一致する割合を算出する。

【設定根拠】

実臨床において、非侵襲的3所見（臨床所見、画像所見、頭皮電極による脳波所見）に加えて、頭蓋内電極の結果から、側方性を診断し、てんかんに対する焦点切除術を行う部位を決める。EP-01は上記実臨床での頭蓋内電極の代替となるかを検証するため、主要評価方法として下記2つの結果の正誤判定を第3者評価によって行うことと設定した。

1.上記非侵襲的3所見とEP-01の脳波所見による側方性診断結果

2.上記非侵襲的3所見と頭蓋内電極の脳波所見による側方性診断結果

また、バイアスがかからないよう、上記1と2は別の評価者によって側方性を診断し、診断結果が同様かを確認する。

内側側頭葉てんかんに対する焦点切除術の術後 Engel I/II は 90%以上である [8]ことから、頭蓋内電極による側方性診断を受けて外科手技による切除術によって 90%以上の発作消失が確認されていると推察できる。このことから頭蓋内電極の側方性診断性能は少なくとも 90%を超えるものと評価する。従って、本治験の主要評価項目に関する対照として頭蓋内電極の側方性結果を使用することは妥当と判断した。

12.2 副次評価項目

12.2.1 EP-01 のみによるてんかん焦点の側方性の診断能：中央判定委員会による第 3 者評価

EP-01 脳波所見のみによる側方性診断結果と、頭蓋内電極脳波所見のみによる側方性診断結果と比較し、一致する割合

【設定根拠】

主要評価項目のうち非侵襲的 3 所見を除く頭蓋内電極と EP-01 の結果のみにおいても結果の正誤判定を行うため設定した。なお、バイアスがかからないように第 3 者評価によって行う。

12.2.2 EP-01 による波形の脳波同定

12.2.2.1 開閉眼時の脳波所見との比較評価：中央判定委員会による第 3 者評価

EP-01 で検出された波形が脳波によるものであるかを確認する。

【設定根拠】

被験者への侵襲を伴わずに EP-01 で検出された波形が脳波によるものであるかを確認するために開閉眼時の脳波所見を確認することとした。

12.2.2.2 EP-01 のみと、EP-01 および頭蓋内電極を入れた状態での脳波測定性能について比較評価：治験責任医師等による評価

EP-01 のみを留置した期間がある場合は、EP-01 のみと、EP-01 および頭蓋内電極を入れた状態での脳波測定性能について比較評価を行うことで、相互影響がないかを確認する。

【設定根拠】

頭蓋内電極と EP-01 を同時に留置することで、脳波測定性能へ影響を及ぼす可能性がある。そこで、EP-01 のみを留置した期間がある症例については、EP-01 のみと、EP-01 および頭蓋内電極を入れた状態での脳波測定性能の比較評価を行い、EP-01 脳波性能の変化の有無を確認するため設定した。

12.2.3 EP-01 によるてんかん焦点の側方性の診断能（頭蓋内電極による診断結果との一致割

合）：中央判定委員会による第 3 者評価
- EP-01 脳波所見および非侵襲的所見による側方性診断結果と、両側に留置した頭蓋内電極脳波所見および非侵襲的所見による側方性診断結果とを比較し、一致する割合
- EP-01 脳波所見のみによる側方性診断結果と、両側に留置した頭蓋内電極脳波所見のみによる側方性診断結果とを比較し、一致する割合

【設定根拠】
主要評価項目および副次評価項目 12.2.1 のうち、EP-01 による頭蓋内電極を両側に留置した側方性診断結果に対する側方性診断結果の正誤判定を行うため設定した。

12.2.4　EP-01 によるてんかん焦点の側方性の診断能：治験責任医師等による評価

非侵襲的所見に、EP-01 の脳波所見を加えた側方性診断結果と、非侵襲的所見に頭蓋内電極の脳波所見を加えた側方性診断結果とを比較し、一致する割合を算出する。

【設定根拠】
主要評価項目の設定根拠参照。担当医師による側方性の診断は実臨床においても行われていることから設定した。

12.2.5　焦点切除後 1 年後の発作消失率

通常診療に従い、非侵襲的所見および頭蓋内電極による脳波所見を用いた焦点側方性診断により行った焦点切除術後 1 年後のてんかん発作転帰評価。ILAE の分類を用いて分布割合を評価する。

【設定根拠】
てんかん焦点切除術の効果判定として、てんかん発作の転帰を評価する方法がある。頭蓋内電極による診断結果に基づきてんかん焦点切除術を行うが、その転帰を調査するため設定した。また、国際的に広く用いられている国際抗てんかん連盟（ILAE）が公表している指標を用いることとした。

12.2.6　EP-01 留置成功率（%）

留置直後の EP-01 本体留置本数／マイクロカテーテル留置本数×100 を算出し成功率として評価する。
抜去直前に EP-01 本体が留置目的血管からの移動有無評価を行う。

【設定根拠】
EP-01 を予定していた血管に留置することができ、問題なく脳波検査を行うことができるかを確認するために設定した。
また、EP-01 留置期間中、先端の電極位置がどの程度目的の留置血管から移動したかを確認するために設定した。

12.2.7　安全性の評価

- 安全性の評価：安全性評価委員会による第 3 者評価

  各実施医療機関で撮像した画像検査等より判断する有害事象*の有無、重症度および機器との関連性を評価する。

  *画像検査等より判断する有害事象例：出血、正中偏移、髄液漏

  さらに、被験機器を起因とする有害事象と頭蓋内電極を起因とする有害事象（両機器留置および抜去のための医学的行為を含む）の発生割合の比較［発現期間：EP-01（本体及び付属品）使用時から後観察期間評価終了時まで］

- 有害事象の発現：治験責任医師等による評価

  ［発現期間：被験機器使用開始から後観察期間終了まで。被験機器全て（EP-01 本体および EP-01 付属品）との関連性、EP-01 留置術、抜去術のための医学的行為との関連性、その他の治験使用機器との関連性、程度、重篤性、処置、転帰］

【設定根拠】
一般的な有害事象の発現状況の情報収集に加えて、被験機器の安全性および侵襲性を評価するため、被験機器を起因とする有害事象と頭蓋内電極を起因とする有害事象との発生割合を比較

することとした。

12.2.8　その他の評価

・被験機器 EP-01 の不具合（EP-01 本体および EP-01 付属品の不具合）
・その他の治験使用機器の不具合（EX-01 エクステンションワイヤー/脳波計）
【設定根拠】
EP-01 を含む本治験で使用する機器の性能を評価するため、上記項目を評価することとした。

13 有害事象及び不具合発生時の取扱い

13.1 有害事象発生時の被験者への対応

治験責任医師等は、有害事象を認めた時は、直ちに適切な処置を行うとともに、診療記録ならびに症例報告書に齟齬なく記載する。また、EP-01 の留置を中止した場合や、有害事象に対する治療が必要となった場合には、被験者にその旨を伝える。

13.2 重篤な有害事象及び不具合の報告

治験責任医師等は、11.1.11 有害事象の観察で重篤性が「重篤」と分類された有害事象及び 11.1.12 不具合の観察に規定する不具合のうち死亡その他の重篤な有害事象を引き起こすおそれのある不具合（実際に重篤な有害事象が発生した場合を含む）の発生を知った場合には、「安全性情報の取扱いに関する手順書」に従い、被験者等への説明等、必要な措置を講じる。

【重篤と判断された有害事象情報の取扱い】

治験中に被験者等に有害事象が発生し、治験責任医師等が当該事象を重篤と判断した場合、次の手順に従い当該有害事象情報を取り扱う。

(1) 治験責任医師から実施医療機関の長、治験調整医師、治験機器提供者及び治験使用機器製造者への報告

治験責任医師（自ら治験を実施する者）は、因果関係に関わらず、当該有害事象情報をすみやかに（原則として情報入手後 24 時間以内）実施医療機関の長および治験調整医師に報告するとともに、治験機器提供者に報告する。

なお、その他の治験使用機器（EX-01 エクステンションワイヤーまたは脳波計 EEG-1200 シリーズ）との因果関係が否定できない当該有害事象の発生を認めた場合は、治験使用機器製造者にも併せて報告する。

(2) 厚生労働大臣への報告

各実施医療機関の治験責任医師と合意のもと、治験調整医師（自ら治験を実施する者）が厚生労働大臣への報告が必要と判断した場合には（必要に応じて各実施医療機関の治験責任医師と協議)、治験調整医師（自ら治験を実施する者）は、「様式第 1」及び「整理票」を作成し、独立行政法人医薬品医療機器総合機構（以下、「総合機構」という）に報告する。

(3) 追加情報の入手時の対応

治験責任医師（自ら治験を実施する者）は、当該事象に関する追加情報が得られた場合には、可能な限り速やかに実施医療機関の長および治験調整医師に追加報告を行うとともに、治験機器提供者に報告する。当該追加情報の取扱いは、上記(1)～(2) の手順に準ずることとし必要に応じ総合機構への報告等を行う。

13.2.1 重篤な有害事象および重篤な健康被害発生のおそれがある不具合の定義

重篤な有害事象とは、有害事象のうち、次のいずれかに該当するものとする。また、重篤な健康被害発生のおそれがある不具合とは、現実に次の死亡、障害等が発生していなくても、発生するおそれがあると治験責任医師等が判断するものとする。

(1) 死亡
(2) 死亡のおそれ
(3) 入院又は入院期間の延長
(4) 障害
(5) 障害のおそれ
(6) 上記に準じて重篤
(7) 後世代における先天性の疾病又は異常

なお、(3) の入院の中でも、本治験実施のための入院又は以下の入院の場合は重篤な有害事象の対象とはならない。

- 治験参加前より予定していた療法を目的とした入院又は入院期間の延長（治験参加前よ

り予定されていた手術や検査など）
- 検査を行うための入院又は入院期間の延長
- 合併症、随伴症状の悪化に起因しないものの、合併症、随伴症状の治療方針が変更となり、手術などを行うための入院又は入院期間の延長
- 治癒又は軽快しているものの経過観察のための入院又は入院期間の延長
- 通常、外来診療にて対処されたであろう有害事象による入院
- 社会的理由による入院又は入院期間の延長（看護人の休養、家族の不在など）

13.2.2 重篤な有害事象及び不具合の報告対象

(1) 被験機器使用開始から後観察期間終了までに発生した、治験使用機器との因果関係が否定できない全ての重篤な有害事象
(2) 治験終了(中止)後に本治験使用機器との関連性が疑われる重篤な有害事象
(3) 治験使用機器の不具合の発生であって、その不具合によって 13.2.1 に掲げる事象が発生した、又は発生するおそれがあるもの
(4) 治験使用機器によるものと疑われる感染症により 13.2.1 に掲げる事象が発生した、又は発生するおそれがあるもの

13.2.3 重篤な有害事象及び不具合の報告手順

13.2.3.1 被験機器（EP-01）

治験責任医師は、本被験機器 EP-01（本体および付属品）との因果関係が否定できない重篤な有害事象及び不具合を「機械器具等に係る治験不具合等報告について（令和 2 年 8 月 31 日薬生発第 0831 号 9 号)」及び「機械器具等に係る治験不具合等報告に関する報告上の留意点等について（令和 2 年 8 月 31 日薬生発第 0831 号 10 号)」に基づき、報告する。報告は第一報（緊急報告）及び第二報（詳細報告）とし、GCP 統一書式（書式14.）を用いることとし、また、当該事象を知りえた日から、以下に示す日までに報告する。詳細は、「安全性情報の取扱いに関する手順書」に従う。

(1) 未知／死亡・死亡につながる恐れのある有害事象：7 日以内。
(2) 未知／その他重篤な有害事象：15 日以内。
(3) 既知／死亡・死亡につながる恐れのある有害事象：15 日以内。
(4) (1)、(2)が発生する恐れがある不具合：30 日以内

13.2.4 治験調整医師の治験機器提供者への報告

治験調整医師は、「安全性情報の取扱いに関する手順書」に従い、重篤な有害事象及び不具合を治験機器提供者に報告する。

13.3 その他の有害事象の報告

その他の有害事象については、本手引きの「11 検査・観察項目及びスケジュール 11.1.11 有害事象の観察」に記載した手順により、症例報告書に記載する。

13.4 その他の治験使用機器（EX-01 エクステンションワイヤー／脳波計 EEG-1200 シリーズ）の不具合・有害事象症例報告

本治験で用いるその他の治験使用機器（EX-01 エクステンションワイヤー／脳波計 EEG-1200 シリーズ）に関しては、本治験において報告された不具合及び有害事象のうち、EX-01 エクステンションワイヤー／脳波計 EEG-1200 シリーズとの関連性が否定できないと判断される死亡及び死亡につながるおそれのあるもの、及び EX-01 エクステンションワイヤー／脳波計 EEG-1200 シリーズとの関連性が否定できない且つ予測できないものと判断されるその他の重篤な不具合・有害事象については、総合機構へ治験機器不具合・有害事象症例報告を実施することとし、予測性については、EX-01 エクステンションワイヤーは治験機器概要書、脳波計 EEG-1200 シリーズは添付文書より判断し、記載のないものを予測できない不具合・有害事象とする。また、本治験において、EX-01 エクステンションワイヤー／脳波計 EEG-1200 シリーズと

の関連性が否定できない重篤な有害事象又は不具合の発生を認めたときは、治験責任医師が把握できる情報を実施医療機関の長および治験調整医師に情報提供する。

14 治験実施計画書からの逸脱の取扱い

(1) 治験責任医師又は治験分担医師は、治験審査委員会の事前の審査に基づく実施医療機関の長の承認を得る前に、治験実施計画書からの逸脱あるいは変更を行ってはならない。

(2) 治験責任医師又は治験分担医師は、緊急回避等のやむを得ない理由により、治験審査委員会の事前の承認を得る前に、治験実施計画書からの逸脱あるいは変更を行うことができる。その際には、治験責任医師又は治験分担医師は、逸脱又は変更の内容及び理由ならびに治験実施計画書等の改訂が必要であればその案を速やかに治験審査委員会に提出し、治験審査委員会及び実施医療機関の長の承認を得るものとする。

(3) 治験責任医師又は治験分担医師は、治験実施計画書からの逸脱があった場合は、逸脱事項をその理由とともに全て記録しなければならない。

(4) 治験責任医師又は治験分担医師は、当該治験について、「医療機器の臨床試験の実施の基準に関する省令（平成 17 年 3 月 23 日厚生労働省令第 36 号）」に適合していないこと（適合していない程度が重大である場合に限る。）を知った場合には、速やかに実施医療機関の長に報告し、必要な対応をした上で、その対応の状況･結果についての実施医療機関の長による厚生労働大臣等への報告･公表に協力しなければならない。

15 中止基準と手順

15.1 治験の終了

治験の終了時に治験責任医師は、速やかに治験終了報告書を実施医療機関の長に提出する。また、「22. 公表に関する取決め」に従い、結果を公表する。

15.2 治験の中止・中断

治験責任医師は、以下の事項に該当する場合は治験実施継続の可否を検討する。

(1) 治験使用機器の品質、安全性、有効性に関する重大な情報が得られた時
(2) 治験の実施の適正性や信頼性を損なう事実又は情報等が得られた場合
(3) 被験者のリクルートが困難で、予定被験者数を達成することが困難であると判断された時
(4) 予定被験者数又は予定期間に達する前に治験の目的が達成された時、又は期待する結果が得られないと判断された時
(5) 治験審査委員会により、治験計画等の変更の指示があり、これを受け入れることが困難と判断された時

また、治験責任医師は、上記の（1）又は（2）に該当する場合は、速やかに実施医療機関の長に報告し、必要に応じて治験実施計画書を変更する。治験審査委員会により、中止の勧告あるいは指示があった場合は、治験を中止する。治験の中止又は中断を決定した時は、速やかに実施医療機関の長にその理由とともに文書で報告する。

15.3 治験中止・終了後の被験者への対応方針

治験責任医師又は治験分担医師は、被験者が治験終了後も最善の治療を受けることができるよう努める。

15.4 個々の被験者における治験脱落・中止基準

下記のいずれかの場合、当該被験者の治験を中止し、適切な処置・治療を実施する。被験機器留置後に中止する場合、被験機器抜去時の検査および後観察期間の検査を可能な限り実施する。なお、個々の被験者の治験を中止する場合、その中止時点で被験者の安全性に配慮した評価を実施し、症例報告書に記載する。

また、被験者に連絡がつかない等の状況が続いた場合、治験責任医師又は分担医師は当該被験者を「追跡不能」とみなし、当該被験者における治験中止を検討する。被験者を「追跡不能」と判断する前に治験責任医師又は分担医師は再度連絡を取るよう最大限の努力を払う。連絡をとるために講じた対応及び追跡不能となった考えられる理由等について原資料に記録する。

(1) 治験責任医師及び治験分担医師により、治験中止が必要と判定された場合
(2) 被験者が被験機器の使用中止を申し出た場合
(3) 被験者が同意を撤回した場合
(4) 被験者に著しく重大な有害事象が発現した場合
(5) 治験開始後に選択基準、除外基準に抵触し、治験実施計画書の遵守が不可能であることが判明した場合
(6) 治験使用機器不具合により、治験続行が困難と判断された場合
(7) 重大な治験実施計画書の逸脱が明らかになった場合

治験中止に至った主な理由については、該当する症例報告書に記載する。
外科的手術を行わない被験者については焦点切除後 1 年後の発作消失率の評価は行わない。

15.5 治験の中止

治験調整医師は、下記の基準を参考に、治験使用機器の品質、有効性及び安全性に関する事項、その他、治験を適正に行う上で障害となるような重要な情報を知った場合、治験の中止を決定し、治験責任医師及び治験実施医療機関の長に文書にて中止及びその理由を通知する。

(1) 新たな安全性情報（重篤な有害事象及び不具合）により、治験継続が困難と判断された場合
(2) 中央判定委員会または安全性評価委員会から治験の中止が進言された場合
(3) 実施医療機関又は治験責任医師又は治験分担医師のいずれかが重大な GCP 違反、治験実施計画書違反などを行い、適正な治験継続に支障を及ぼすと判断された場合
(4) 治験実施体制の変更（例：治験調整医師／治験責任医師の異動など）により、治験継続が困難と判断された場合
(5) その他、治験実施中の新たな情報又は情勢の変化により治験継続が不適切と判断された場合

16 統計解析

16.1 目標症例数の設定

予定登録数は被験機器使用症例として 37 例とする。

【設定根拠】

頭蓋内電極留置術に対する EP-01 の側方性診断の一致割合 p に対して、帰無仮説を p=0.8、対立仮説を p>0.8 とし、仮説検定は二項割合の正確な片側検定により行う。期待される一致割合を 0.97、有意水準を 0.025、検出力を 90%とすると、必要症例数は 34 例となる。10%の脱落を考慮して、目標症例数を 37 例とした。

16.2 データの取扱い基準

被験者の取扱いに関する詳細を以下に示す。

(1) 未介入例

登録した被験者のうち、被験機器を使用しなかった被験者を未介入例とする。

(2) 不適格例

選択基準に違反又は、除外基準に抵触する被験者を不適格例とする。

(3) GCP 不遵守例

治験審査委員会で審査されていない、治験契約が締結されていない、あるいは適切なインフォームド・コンセントが得られていない被験者などを GCP 不遵守例とする。

上記以外の予測されなかった問題について、治験責任医師は、データ固定までに内容に関し検討の上、解析上の取扱いを決定する。

16.3 解析対象集団

16.3.1 安全性解析対象（Safety Analysis Set：SAF）

登録された被験者のうち、未介入例を除外した被験者の集団を安全性解析対象集団と定義する。

16.3.2 有効性解析対象

(1) 最大の解析対象集団（Full Analysis Set、FAS）

登録された被験者のうち、以下の条件に該当する被験者を除外した集団を FAS と定義する。

- 未介入例
- 被験機器使用後の有効性データが全くない
- GCP 不遵守例

(2) 治験実施計画書に適合した解析対象集団（Per Protocol Set、PPS）

FAS のうち、以下の条件に該当する被験者を除外した集団を PPS とする。

- 不適格例

16.4 被験者背景の解析

連続変数は要約統計量、カテゴリカル変数は頻度と割合を用いて集計する。

16.5 主要評価項目の解析

EP-01 によるてんかん焦点の側方性の診断結果と頭蓋内電極による診断結果との一致割合と、Clopper-Pearson 法による一致割合の 95%信頼区間を求める。なお、主たる解析対象は FAS である。

16.6 副次評価項目の解析

16.6.1 有効性副次評価項目

- EP-01 脳波所見のみによる側方性診断結果と、頭蓋内電極脳波所見のみによる側方性診断結果との一致割合を求める。
- EP-01 で検出された波形が脳波によるものである割合を求める。
- EP-01 脳波所見および非侵襲的所見による側方性診断結果と、両側に留置した頭蓋内電極脳波所見および非侵襲的所見による側方性診断結果との一致割合を求める。
- EP-01 脳波所見のみによる側方性診断結果と、両側に留置した頭蓋内電極脳波所見のみによる側方性診断結果との一致割合を求める。
- 侵襲的所見に、EP-01 の脳波所見を加えた側方性診断結果と、非侵襲的所見に頭蓋内電極の脳波所見を加えた側方性診断結果との一致割合を求める（治験責任医師等による評価）。
- 焦点切除後 1 年後の発作消失割合を求める。
- EP-01 留置成功割合を求める。

16.6.2 安全性副次評価項目

有害事象、被験機器を起因とする有害事象、重篤な有害事象、被験機器を起因とする重篤な有害事象の有無に関して、発現件数、発現例数とその割合を集計する。

16.7 中間解析

本治験で中間解析は行わない。

17 治験実施計画書の遵守及び改訂

17.1 治験実施計画書の遵守及び逸脱又は変更

(1) 治験責任医師及び治験分担医師は、治験審査委員会の事前の審査に基づく文書による承認を得ることなく、治験実施計画書からの逸脱又は変更を行わない。

(2) 治験責任医師及び治験分担医師は、治験実施計画書から逸脱した行為を全て記録する。

(3) 治験責任医師及び治験分担医師は、被験者の緊急の危険を回避するためのものであるなど医療上やむを得ない事情のために、治験審査委員会の事前の承認なしに治験実施計画書からの逸脱又は変更を行うことができる。その際には、治験責任医師は、逸脱又は変更の内容及び理由並びに治験実施計画書の改訂が適切である場合にはその案を可能な限り早急に実施医療機関の長及び治験審査委員会に提出してその承認を得るとともに、実施医療機関の長の了承を文書で得る。

(4) 治験責任医師は、治験の実施に重大な影響を与え、又は被験者の危険を増大させるような治験のあらゆる変更について、実施医療機関の長及び治験審査委員会に速やかに報告書を提出する。

17.2 治験実施計画書の改訂

治験責任医師は、治験使用機器の品質、有効性及び安全性に関する事項、その他の治験を適正に行うために重要な情報を知った場合、必要に応じ治験実施計画書あるいは説明文書を改訂する。

治験責任医師は、治験審査委員会の事前の審査に基づく文書による承認を得た後に、改訂された治験実施計画書に従って治験を行う。但し、医療上やむを得ないものである場合や治験の事務的事項（所属、職名、住所、電話番号の変更等）に関する変更である場合には、この限りではない。

18 治験の品質管理及び品質保証

18.1 品質管理

18.1.1 原資料の直接閲覧

治験責任医師及び治験実施医療機関は、モニタリング、監査、ならびに治験審査委員会及び規制当局による調査を受け入れなければならない。これらの場合には、モニター、監査担当者、治験審査委員会及び規制当局の求めに応じて、原資料等の全ての治験関連記録を直接閲覧に供しなければならない。なお、原資料の閲覧に際しては被験者のプライバシー保護に十分留意する。

18.1.2 原資料の特定

本治験では、以下の原資料と症例報告書の入力内容を照合する。これらの原資料には、症例報告書の元となる情報（原データ）が記録されているものとする。

(1) 診療録及びこれに貼付された記録類
(2) 検査伝票、ワークシート
(3) 同意書
(4) 被験機器管理表、被験機器受払の記録

なお、症例報告書のコメントについては、症例報告書の記載内容を原データとする。

18.1.3 データマネジメント

データマネジメント責任者は、集積するデータの品質管理を実施する。具体的には、実施医療機関がカルテなどの原資料に基づき収集したデータの、症例報告書内不整合をチェックする。疑義が発生した場合には、必要に応じて治験責任医師／分担医師にデータの照会をする。データの品質確認終了後にデータを固定する。詳細はデータマネジメント計画書に従う。

18.1.4 モニタリング

治験責任医師は、被験者の人権、安全及び福祉が保護されていること、治験が最新の治験実施計画書及び GCP および ISO14155 を遵守して実施されていること、及び治験データが正確かつ完全で、原資料等の治験関連記録に照らして検証できることを確認するためモニタリングを実施させる。治験責任医師は、モニタリングが適正に実施されるよう、モニターを指名する。モニターは、あらかじめ作成されたモニタリング手順書に従ってモニタリングを実施する。

18.2 品質保証

品質保証とは、治験の実施、データ作成、文書化及び報告が GCP 及び関連する規制を遵守していることを保証するために設定された計画的かつ体系的な活動である。

通常のモニタリング又は治験の品質管理業務とは独立して、治験責任医師が指名する監査担当者は、得られたデータ及び治験に係る業務について、別途に定める「監査に関する手順書」に従い、確認した事項を記録した監査報告書及び監査証明書を作成し、治験責任医師及び実施医療機関の長に提出する。この業務には、外部監査及び原資料の確認が含まれる。また、原資料の直接閲覧は、監査の目的上、必要なものである。

19 倫理

19.1 治験の倫理的実施

本治験は、ヘルシンキ宣言に基づく倫理的原則、医薬品医療機器等法、医薬品の臨床試験の実施の基準に関する省令（平成 9 年 3 月 27 日厚生省令第 28 号）、医療機器の臨床試験の実施の基準に関する省令（平成 17 年 3 月 23 日付厚生労働省令第 36 号）に従い、本治験実施計画書を遵守して実施する。

被験者の人権、安全及び福祉に対する配慮が最も重要であり、科学と社会のための利益よりも優先されるべきである。

治験審査委員会は、治験について倫理的、科学的及び医学的観点から治験の実施及び継続等について十分に審議する。

治験実施医療機関の長は、当該医療機関における治験が GCP 省令、治験実施計画書、治験契約書及び当該医療機関の手順書に従って適正にかつ円滑に行われるよう必要な措置を講じる。治験責任医師は、治験審査委員会の承認に基づく当該医療機関の長の指示、決定に従い、治験実施計画書を遵守して治験を実施する。治験責任医師及び治験分担医師は、被験者の選定にあたって、人権保護の観点から及び治験実施計画書に基づき、被験者の健康状態、症状、年齢、同意能力、治験責任医師又は治験分担医師との依存関係等を考慮する。同意能力を欠く者については、当該治験の目的上やむを得ない場合を除き、原則として被験者としない。また、社会的に弱い立場にあると考えられる者を被験者とする場合には特に慎重な配慮をする。

治験責任医師は、被験者の治験に関連する医療上の判断の責任を負うものである。治験責任医師及び治験分担医師は、被験者が治験に参加する前に、あらかじめ治験の内容、その他の治験に関する事項について、理解を得るように被験者に対して説明文書・同意文書を用いて十分に説明し、治験への参加について自由意思による同意を文書により得る。治験に継続して参加するか否かについて、被験者の意思に影響を与える可能性のある情報が得られた場合、治験責任医師及び治験分担医師は、当該情報を速やかに被験者に伝え、治験に継続して参加するか否か、被験者の意思を確認する。

被験者の身元を明らかにする可能性のある記録は、被験者のプライバシーと秘密の保全に配慮して保護する。

19.2 被験者の秘密保全

治験実施医療機関の長は、被験者の秘密の保全が担保されるよう必要な措置を講じる。被験者に関わる情報は、被験者を特定し得るものの使用を禁止するなど、本治験に関わる者は、被験者のプライバシー保護に最大限の注意を払わなければならない。また、モニター、監査担当者を含めて本治験に関与した者は、原資料等の直接閲覧などにより被験者のプライバシーに関わる事項を知り得た場合にも、その内容をいかなる第三者にも漏洩してはならない。

19.3 被験者・代諾者への説明と同意

19.3.1 同意文書及びその他の説明文書

治験責任医師又は治験分担医師は、治験実施に先立ち、治験審査委員会にて承認を得た同意・説明文書を用い、対象となる患者または代諾者*に対して、口頭又は文書による十分な説明を行い、患者の自由意思による同意を取得する。また、被験者または代諾者*の同意に影響を及ぼすと考えられる有効性や安全性等の情報が得られた時や、被験者または代諾者*の同意に影響を及ぼすような実施計画等の変更が行われる時は、速やかに被験者または代諾者*に情報提供し、本治験に継続して参加するか否かについて被験者または代諾者*の意思を予め確認するとともに、事前に治験審査委員会の承認を得て説明文書・同意書等の改訂を行い、被験者または代諾者*の再同意を取得する。

*：代諾者は、被験者に十分な同意の能力がない場合または被験者が 18 歳未満の患者を対象とする場合、治験への参加について、被験者とともに、又は被験者に代わって同意をすることが正当なものと認められる者である。患者の親権を行う者、配偶者、後見人、その他これらに準じる者とする。

19.3.2 アセント説明文書およびその他の説明文書

治験責任医師又は治験分担医師は、治験実施に先立ち、治験審査委員会にて承認を得たアセント説明文書を用い、対象となる患者のうち未成年や、説明文書の理解の助けが必要な被験者に対して、口頭又は文書による十分な説明を行い、患者の自由意思によるインフォームド・アセントを取得する。なお、可能な限り文書によるインフォームド・アセントを取得するまた、被験者の意思に影響を及ぼすと考えられる有効性や安全性等の情報が得られた時や、被験者の意思に影響を及ぼすような実施計画等の変更が行われる時は、速やかに被験者に情報提供し、本治験に継続して参加するか否かについて被験者の意思を予め確認するとともに、事前に治験審査委員会の承認を得てアセント文書等の改訂を行い、被験者のインフォームド・アセントを再び取得する。

19.3.3 説明文書およびアセント文書の内容

同意説明文書には、患者または代諾者が理解しやすい表現に配慮し、以下の説明事項を含めて記載する。アセント説明文書は当該項目を対象者が理解できる平易な表現で記載する。

(1) 治験が研究と伴うこと
(2) 治験の目的
(3) 治験責任医師の氏名、職名及び連絡先
(4) 治験の方法
(5) 予期される臨床上の利益及び危険性又は不利益（被験者にとって予期される利益がない場合には、被験者にその旨を知らせること）
(6) 他の治療方法に関する事項
(7) 治験に参加する期間
(8) 治験の参加をいつでも取りやめることができること
(9) 治験に参加しないこと又は参加を取りやめることにより被験者が不利益な取扱いを受けないこと
(10) 治験の参加を取りやめる場合の被験機器の取扱いに関する事項
(11) 被験者の秘密が保全されることを条件に、モニター、監査担当者及び治験審査委員会等が原資料を閲覧できること
(12) 被験者に係る秘密が保全されること
(13) 健康被害が発生した場合における実施医療機関の連絡先
(14) 健康被害が発生した場合に必要な治療が行われること
(15) 健康被害の補償に関する事項
(16) 当該治験の適否等について調査審議を行う治験審査委員会に関する事項
(17) 被験者が負担する治験の費用に関する事項
(18) 被験者が守るべき事項

19.3.4 同意およびアセント取得の時期と方法

本治験への参加に対する文書同意およびアセントは、本治験の規定のスクリーニング検査又は評価を実施する前に取得しなければならない。対象となる患者本人または代諾者に文書を用いて十分に説明し、患者または代諾者が内容をよく理解したことを確認した上で、本治験への参加について患者本人または代諾者の自由意思による同意を文書にて得ることとする。

同意文書およびアセンド文書には説明を行った治験責任医師又は治験分担医師及び患者本人が署名し、各自日付を記入する。治験協力者が補足的な説明を行った場合には、当該治験協力者も同意文書に署名し、日付を記入する。実施医療機関に特別の取り決めがある場合を除いて、同意文書原本は原資料として保存し、写しを説明文書とともに患者へ手渡す。

19.3.5 同意に関する留意事項

書字が困難な患者でも、可能な限り同意文書に署名し、自ら日付を記入する。患者に対して同意文書及び説明文書が渡され、その内容が口頭又は他の伝達方法により説明され、患者が治験への参加に口頭で同意し、更に、代諾者又は立会人*が同意文書に署名し、自ら日付を記入する。なお、患者本人の氏名欄は空欄とし、代筆者（立会人）又は治験責任医師あるいは治験分担医師は余白などに患者氏名、患者が自筆による署名ができない背景、患者と立会人との関係を記載する。

*：治験の実施から独立し、治験に関与する者から不当に影響を受けない者で、インフォームド・コンセントの過程に立ち会う者である。治験責任医師、治験分担医師及び治験協力者は該当しない。

19.3.6 同意およびアセント説明文書の改訂

新たに治験参加の継続について被験者の意思に影響を与える可能性のある重要な情報（同意文書、説明文書及びアセント説明文書の改訂を必要とするような被験者一般にとって重要と考えられる情報）が得られた場合、治験責任医師は速やかに同意文書及び説明文書を改訂し、治験審査委員会の承認に基づく実施医療機関の長の決定の通知を得る。また、既に治験に参加している被験者に対しても速やかに、改訂された同意文書及び説明文書を用いて改めて説明し、治験への参加の継続についての同意を文書により得る。ただし、改訂時点で既に観察が終了した被験者については、同意を不要とする。

治験の継続参加について、被験者の意思に影響を与える可能性のある情報（治験に参加している被験者個人における臨床検査値の変動などの治験経過に関する情報）が得られた場合には、治験責任医師又は治験分担医師は当該情報を速やかに被験者に伝え、治験への参加の継続についての意思を確認する。また、被験者に対して情報が伝えられた日、伝えられた情報の内容及びその確認結果を診療録などの原医療記録に記録する。

19.4 治験審査委員会

本治験の実施に先立ち、治験実施医療機関の治験審査委員会において、治験実施計画書、被験者の同意を得るのに使用される各種資料の審議・承認を受けることによって、被験者の人権、安全性及び福祉の保護を保障する。

19.5 中央判定委員会

治験責任医師は、第 3 者による有効性に関する評価を目的として、中央判定委員会を設置する。中央判定委員会は「中央判定委員会に関する手順書」に従い、てんかん焦点の側方性診断等を評価する。

有効性評価目的および時期	第 3 者によるてんかん焦点の側方性診断について、バイアス評価の疑いを避けるため、第 3 者の評価を用いることが目的。評価は、てんかん焦点の側方性診断結果に関して治験責任医師等の判定終了後に実施する。<br>実施時期：症例のデータを各実施医療機関から中央判定委員会事務局を通じて中央判定医へ送付された後、都度実施する。

上記業務において、中央判定委員会が他の専門家の意見聴取が必要と判断した場合は、治験調整医師へ連絡の上、他の専門家にオブザーバー参加を依頼し意見を聴取する。

19.6 安全性評価委員会

治験調整医師は、第 3 者による安全性評価を行う目的として、安全性評価委員会を設置する。安全性評価委員会は「安全性評価委員会に関する手順書」に従い、各実施医療機関で取得されたデータをもとに安全性を評価する。

表 19-1 安全性委員会評価事項

安全性に関する評価目的および時期	安全性に関する評価を行うとともに、治験継続の適否について評価することが目的。<br>(1) 定期評価<br>各実施医療機関で撮像した画像検査等により判断する有害事象*の有無、重症度および機器との関連性を評価する。<br>*画像検査等により判断する有害事象例：出血、正中偏移、髄液漏<br>安全性評価判定は、各実施医療機関から治験調整事務局へ CT 画像データが提出された後、治験実施計画書に定めた定期開催時期に実施する。<br>・定期第 1 回目…15～20 症例（※症例数に応じて修正）の症例データが各実施医療機関から安全性評価委員会事務局へ提出された時点で開催調整を実施<br>・定期第 2 回目…全症例の症例データが各実施医療機関から安全性評価委員会事務局へ提出された時期<br><br>(2) 臨時評価<br>被験機器を起因とする重篤な有害事象が認められた等による有害事象の発現状況及び治験機器提供会社から提供された安全性情報等を基に、治験調整医師が必要と判断した場合、臨時開催として実施する。<br>各実施医療機関から報告された被験機器を起因とする重篤な有害事象に加え、以下を基に、治験調整医師が必要と判断した場合、当該治験の継続等及び治験実施計画書の変更等についての提言を行う。<br>・ 治験全体の継続等に関わる新たな重要な安全性情報の入手時<br>・ 当初の予測を大幅に上回る有害事象（又は副作用）又は不具合の発生時<br>・ 当初の予測より有効性が著しく高いあるいは低い状況の発生時<br>・ 安全性若しくは有効性に係る新たな重大な情報の入手時<br><br>(3) その他<br>その他治験調整医師が必要と判断した場合に開催する。

上記業務において、安全性評価委員会が他の専門家の意見聴取が必要と判断した場合は、治験調整医師へ連絡の上、他の専門家にオブザーバー参加を依頼し意見を聴取する。

19.7 利益相反

19.7.1 研究資金の提供

本治験は、株式会社 Epsilon Medical 及び日本医療研究開発機構　医療機器開発推進研究開発事業「血管内脳波測定デバイスの多施設共同医師主導治験」（研究開発代表者：筑波大学 松丸祐司）からの支援を受け実施する。

本治験の実施にあたり、治験責任医師及び治験分担医師は、実施医療機関が別途定める利益相反に関する規定に従って自己申告をするものとする。

19.7.2 被験機器の提供

本治験は、株式会社 Epsilon Medical との医師主導治験に関する契約に基づき、株式会社 Epsilon Medical より本治験に係る被験機器（EP-01）およびその他の治験使用機器（EX-01 エクステンションワイヤー）の無償提供を受け実施する。また、被験機器（EP-01）の安全性情報等も株式会社 Epsilon Medical から提供される。

19.7.3 治験取得データの提供

本治験において取得されたデータは、任意の同意が得られた被験者のみ、筑波大学と株式会社 Epsilon Medical の契約に従って株式会社 Epsilon Medical へ提供される。

なお、株式会社 Epsilon Medical による治験データの利用は、有害事象や不具合のための調査、Epsilon Medical 社製品の機器の安全性・有効性の確認と改善のための研究開発を目的とし、治験調整医師の指示及び許可を受けたときのみとする。

20 治験に関する記録及びその保存

20.1 症例報告書

20.1.1 本治験で用いる症例報告書の様式

治験責任医師及び治験分担医師は、本治験への参加を同意取得した全ての被験者に対して、被験者の各観察終了後、速やかにデータを症例報告書へ、診療録等の原資料と矛盾しないよう記載する。治験協力者が記録する場合、原資料からの転記にとどめる。治験責任医師は症例報告書の記載内容の全てが正確、完全であることを確認し、症例報告書に署名を行うことにより保証する。

20.2 記録の保存

20.2.1 治験責任医師

治験責任医師は、以下に掲げる記録を「記録の保存に関する手順書」に従って保存する。

(1) 治験実施計画書、総括報告書、症例報告書、その他 GCP 省令の規定により治験責任医師又は治験分担医師が作成した文書
(2) 治験実施医療機関の長から通知された治験審査委員会の意見に関する文書、その他 GCP および ISO14155 の規定により実施医療機関の長から入手した記録
(3) モニタリング、監査その他治験の実施の準備及び管理に係る業務の記録
(4) 治験を行うことにより得られたデータ
(5) 治験使用機器に関する記録
(6) 監査業務に関する記録

20.2.2 実施医療機関

治験実施医療機関の長は、治験実施医療機関において保管すべき治験に関する記録（文書を含む）を当該被験機器に係る製造販売の承認を受ける日又は治験の中止若しくは終了の後 10 年を経過した日、又は、実施医療機関において規定された日のうちいずれか遅い日までの期間、適切に保存するものとする。なお、保存期間延長をする場合には、別途取り決めをする。

治験実施医療機関で保存しなければならない主な記録は下記のものである。

(1) 原資料（症例報告書の元となる文書、データ及び記録）
病院記録、診療録、検査ノート、メモ、被験機器使用記録、治験使用機器使用記録、自動計器の記録データ、正確な複写であることが検証によって保証された複写物又は転写物、写真のネガ、マイクロフィルム又は磁気媒体、エックス線写真、被験者ファイル及び治験に関与する各検査室等に保存されている記録、外注測定を含む検査伝票、ワークシート、CRC 記録、他科・他院に確認した記録等
(2) 契約書、同意文書及び説明文書、アセント文書、その他 GCP の規定により実施医療機関に従事するものが作成した文書又はその写し、治験契約書、中止・終了報告書、説明文書・同意文書（改訂版を含む）、署名・日付を記入した同意文書（改訂版を含む）
(3) 治験実施計画書（改訂版を含む）、治験審査委員会等から入手した文書その他 GCP の規定により入手した文書、治験機器概要書（改訂版を含む）、治験責任医師業務手順書、治験責任医師から提供された資料・情報、治験責任医師からの報告事項（治験実施計画書変更、有害事象報告等）、治験審査委員会の業務手順書、委員名簿、審査資料、審議記録、審査結果報告書等
(4) 治験使用機器の管理その他の治験に係る業務の記録
(5) 原料組織採取手順、治験機器の管理に関する手順書、治験機器管理表、治験機器納品依頼書、治験機器受領・回収伝票等

21 被験者の健康被害補償及び治験参加に伴う負担軽減

21.1 健康被害補償

本治験により、被験者に何らかの健康被害が生じた場合、その程度、期間に応じた適切な補償を被験者に行う。ただし、治験実施医療機関に故意又は重大な過失がある場合などにおいては、治験実施医療機関との契約に基づき、その取扱いにつき協議する。健康被害の補償については以下の手順で実施する。

(1) 本治験に起因して被験者に何らかの健康被害が生じた場合は、治験実施医療機関は治療その他必要な措置を講ずるとともに治験責任医師へ連絡する。
(2) 本治験による被験者の健康被害に関し、被験者から実施医療機関に対し補償又は賠償の請求があった場合、又はなされる可能性がある場合、治験実施医療機関は直ちに治験責任医師へ連絡し、両者協力してその解決に当たる。
(3) 治験責任医師は、治験に起因して被験者に健康被害があった場合は、治験責任医師が定める「被験者の健康被害補償に関する手順書」に従って補償を行う。
(4) 治験責任医師は、治験契約書の定めに従い、健康被害に関する補償金又は賠償金及びその解決に要した費用を負担する。
(5) 本治験による被験者の健康被害であって、後に賠償責任が生じた場合には、当該賠償責任のある者の責任と負担において賠償を行う。
(6) 治験責任医師は、上記補償又は賠償のために、保険その他必要な措置を講ずる。

21.2 被験者の治験参加に伴う負担の軽減

本治験で使用する被験機器は、治験機器提供者である株式会社 Epsilon Medical から無償で提供される。本治験は通常臨床で行われる入院および検査、画像診断の範囲内で主に行われるが、治験参加に伴い追加で発生する被験機器留置および抜去手技に伴う費用、検査・画像診断費用は治験実施医療機関が負担する。その他の診療費用は、被験者の健康保険に基づき被験者が負担する。治験参加に伴う被験者負担軽減費として、同意取得された被験者へ原則として本治験実施計画書で規定した検査日に限り支給する。

22 公表に関する取決め

本治験に関する情報は被験者募集を開始する前に臨床研究等提出・公開システム（Japan Registry of Clinical Trials）へ登録する。

本治験結果は、治験総括報告書として治験調整医師がまとめる。後観察期間までの評価結果を 1 次解析結果、てんかん発作転帰評価までの評価結果を 2 次解析結果とし段階を分けて総括報告書を作成し、また、必要に応じ、論文又は学会発表として公表する。なお、本治験実施計画書に従って実施された治験成績に対する権利は、筑波大学が所有するものとする。治験責任医師及び治験分担医師及び治験実施医療機関による治験成績の公表は、治験調整医師との合意の下に実施する。また、治験成績公表の際、被験者の名前が直接公表されることがない等、被験者のプライバシーの保護については十分に配慮する。

23 治験実施予定期間

被験機器評価期間
2024 年 3 月～2025 年 8 月
てんかん焦点切除術によるてんかん発作転帰評価期間を含めた期間
2024 年 3 月～2026 年 8 月

24 参考文献

1) 日本神経学会. てんかん診療ガイドライン 2018
2) 大槻泰介. 平成 25 年てんかんの有病率等に関する疫学研究及び診療実態の分析と治療体制の整備に関する研究. 厚生労働省科学研究費補助金 総合研究報告書.
3) Dario J. Englot et al. A Modern Epilepsy Surgery Treatment Algorithm: Incorporating Traditional and Emerging Technologies. Epilepsy Behav. 2018 March ; 80: 68–74.
4) 岩崎真樹.てんかん外科における頭蓋内脳波の適応. 脳神経外科, 47(1) : 5 - 14, 2019
5) Khoo HM, Kishima H et al. Technical Aspects of SEEG and Its Interpretation in the Delineation of the Epileptogenic Zone. Neurol Med Chir (Tokyo). 2020 Dec 15;60(12):565-580.
6) 前澤聡ら.てんかん外科の現状と展望.現代医学 69 巻 2 号.令和 4 年 12 月(2022)
7) 渡辺英寿.内側側頭葉てんかんの診断と手術適応に関するガイドライン.日本てんかん学会
8) 渡辺英寿.わが国におけるてんかん外科の現状.脳神経外科速報 17(1) :74-81,2007.
9) Crandall Phet al. Clinical applications of studies on stereotactically implanted electrodes in temporallobe epilepsy. J Neurosurg. 1963 Oct;20:827-40.
10) Fujimoto A et al. False lateralization of mesial temporal lobe epilepsy by noninvasive neurophysiological examinations. Neurol Med Chir (Tokyo). 2006 Oct;46(10):518-21.
11) Engel J Jr, Wiebe S et al. Practice parameter: temporal lobe and localized neocortical resections for epilepsy. Epilepsia. 2003 Jun;44(6):741-51.
12) Wiebe S, Blume WT, Girvin JP, Eliasziw M; Effectiveness and Efficiency of Surgery for Temporal Lobe Epilepsy Study Group. A randomized, controlled trial of surgery for temporal-lobe epilepsy. N Engl J Med. 2001 Aug 2;345(5):311-8.
13) 中瀬裕之ら.脳血管内脳波にて棘波をとらえた側頭葉てんかんの 2 例.脳神経 45(10):973-977,1993.
14) H. G. Wieser, W. T. Blume et al. Commission Report Commission on Neurosurgery of the International League Against Epilepsy (ILAE) 1997–2001

25 別添

25.1 別添 1 ILAE の分類

外科的治療後のてんかん発作転帰評価を ILAE の分類を以下に示す。

ILAE 分類

ClassI	Completely seizure free; no auras
ClassII	Only auras; no other seizures
ClassIII	One to three seizure days per year; ± auras
ClassIV	Four seizure days per year to 50% reduction of baseline seizure days; ± auras
ClassV	Less than 50% reduction of baseline seizure days to 100% increase of baseline seizure days; ± auras
ClassVI	More than 100% increase of baseline seizure days; ± auras

参考文献

14）ILAE Commission Report
