# Supplementary material for: Endovascular EEG device prospective multicenter single-arm clinical trial to confirm efficacy and safety performance on patients with Intractable Epilepsy: The EPSILON IE trial protocol
Source: PLoS One. 2025 Oct 16;20(10):e0332387. doi: 10.1371/journal.pone.0332387 (PMC12530528; doi:10.1371/journal.pone.0332387)
Supplement: S3 File — (DOCX) [file pone.0332387.s003.docx]

Consent is obtained for patients enrolled in the EPSILON IE study using a patient information document previously approved by the Ethics Review Committee.

The following is a summary of the patient information document. The risks of the device are detailed in section 5 of the patient information document and are described below.

**Summary of Informed Consent**

This document is an "Explanation and Consent Form" for a clinical trial involving an intracranial EEG measurement device for patients with refractory epilepsy. Below is the summary:

**Overview**

- **Institution**: University of Tsukuba Hospital
- **Trial Title**: Multicenter Prospective Single-Arm Trial to Confirm the Efficacy and Safety of an Intracranial EEG Measurement Device for Patients with Refractory Epilepsy
- **Version**: 2.2
- **Date**: May 24, 2024

**A. Summary of the Clinical Trial**

1. **Purpose**: To confirm the efficacy and safety of the intracranial EEG measurement device in diagnosing the location of epilepsy foci in patients with refractory epilepsy.
2. **Device**: A metal wire approximately 0.25mm in diameter, placed in the brain's blood vessels to measure EEG.
3. **Duration**: Screening (up to 30 days), trial period (about 2 weeks), post-observation (about 1 week), and follow-up (1 year after surgical treatment).
4. **Participants**: Approximately 37 patients across multiple medical institutions in Japan.
5. **Costs**: No cost for the trial device; some costs covered by insurance.

**B. General Information about Clinical Trials**

1. **Definition**: Clinical trials are conducted to confirm the efficacy and safety of new drugs or medical devices.
2. **Voluntary Participation**: Participation is voluntary, and participants can withdraw at any time without any disadvantage.
3. **Contact Information**: Provided for trial physicians and clinical research coordinators.
4. **Costs and Compensation**: Details on costs covered by the trial and insurance, and compensation for participation.

**C. Specific Information about This Trial**

1. **Disease and Treatment**: For patients with refractory epilepsy who are scheduled for intracranial EEG testing.
2. **Trial Device**: Developed by Epsilon Medical, used to measure EEG from within blood vessels.
3. **Trial Method**: Includes criteria for participation, trial procedures, and schedule.
4. **Expected Benefits and Risks**: No direct benefits to participants; potential future benefits for other patients. Risks include those associated with the device and procedures.
5. **Alternative Treatments**: Standard pre-surgical tests like WADA test and intracranial EEG.
6. **Trial Termination**: Conditions under which the trial may be terminated.
7. **Participant Responsibilities**: Follow instructions, report any health changes, avoid MRI during the trial, etc.
8. **Conflict of Interest**: Disclosure of any financial interests related to the trial.
9. **Intellectual Property**: Rights belong to the University of Tsukuba Hospital.

**D. Additional and Detailed Information**

1. **Investigator-Initiated Trials**: Explanation of trials planned and conducted by physicians.
2. **Data Handling**: Information on data storage, participant rights, cross-border data transfer, and data provision to external parties.

**Consent Form**

- **Details**: Participants must sign the consent form after understanding the trial details.
- **Compensation**: Participants can choose to receive compensation for participation-related expenses.
- **Data Provision**: Participants can consent to provide data to Epsilon Medical.

**Summary of Section5**

**Chapter 5: Expected Benefits and Risks**

**5-1. Expected Benefits**

- **Direct Benefits**: There are no direct benefits expected for the participants.
- **Future Benefits**: The results of this trial may confirm that intracranial EEG measurement using the trial device is comparable to traditional intracranial electrode EEG. This could potentially eliminate the need for invasive intracranial electrode EEG, reducing the physical burden on patients and shortening the time to surgical treatment. This advancement could benefit future patients with refractory epilepsy.

**5-2. Expected Side Effects or Adverse Events**

- **Risks Associated with the Trial Device**:
  - **Insertion Site of Microcatheters**: Redness, swelling, bruising, bleeding, infection, discomfort, or pain.
  - **Use of Contrast Agents**: Allergic reactions (itching, rash, difficulty breathing, severe drop in blood pressure).
  - **Radiation Exposure**: Skin damage, eye damage.
  - **Use of Anesthetics**: Nausea, vomiting, headache, chills, fever.
  - **Other Risks**: Bleeding, vascular dissection, vascular spasm, thrombosis, infection, metal allergy reactions, stroke, kidney dysfunction, epileptic seizures, agitation, fever.
- **Risks Associated with Trial Procedures**:
  - **CT and X-ray Examinations**: Radiation exposure.
  - **Use of Contrast Agents**: Potential for allergic reactions.

**5-3. Expected Disadvantages**

- **Increased Number of Tests and Visits**: In addition to standard tests (WADA test and intracranial electrode EEG), the trial device tests will be added, potentially increasing the number of tests and visits required.

This chapter details the expected benefits and risks of participating in the trial, including potential side effects and adverse events. It is crucial for participants to fully understand these risks and benefits before deciding to participate. If you have any questions, please consult the trial physician or clinical research coordinator.
