# Supplementary material for: Endovascular EEG device prospective multicenter single-arm clinical trial to confirm efficacy and safety performance on patients with Intractable Epilepsy: The EPSILON IE trial protocol
Source: PLoS One. 2025 Oct 16;20(10):e0332387. doi: 10.1371/journal.pone.0332387 (PMC12530528; doi:10.1371/journal.pone.0332387)
Supplement: S4 File — (DOCX) [file pone.0332387.s004.docx]

**Summarized Instructions for Primary Outcome Measure Assessment**

The primary endpoints of the EPSILON IE study are assessed by an independent third-party committee. The procedure document outlining the procedure is summarized below.

**Purpose and Scope**

- **Purpose**: To establish procedures for the Central Review Committee to conduct appropriate evaluations.
- **Scope**: Procedures and necessary matters for trial sites, coordinating investigators, and the committee.

**Definitions**

- **Coordinating Investigator**: A physician responsible for coordinating the details of the trial protocol at the trial site.

**Committee Establishment and Evaluation Purpose**

- **Purpose**: To evaluate epilepsy focus in EEG data from an independent standpoint and ensure trial integrity.
- **Evaluation Content**: Diagnosis of epilepsy focus laterality and identification of EEG waveforms by EP-01.

**Committee Composition**

- **Composition**: Four independent epilepsy specialists not directly involved in the trial, including two EEG evaluators for EP-01 and two for intracranial electrodes.
- **Chairperson**: Appointed by the coordinating investigator from among the committee members.

**Requirements and Term of Office for Members**

- **Requirements**: Independence from conflicts of interest, necessary knowledge and experience, and availability throughout the trial.
- **Term**: Until the trial's final report is completed.

**Secretariat Establishment**

- **Role**: Organize and manage committee meetings, prepare and store evaluation materials.

**Central Review Target Cases**

- **Target**: Subjects who have consented to participate in the trial, with clinical findings, imaging findings, and EEG findings obtained within one year of consent.

**Evaluation Items**

- **Content**: Evaluation of epilepsy focus laterality using EP-01 EEG findings and intracranial electrode EEG findings.

**Confidentiality**

- **Obligation**: Committee members and secretariat staff must maintain confidentiality regarding subject information.

**Document Storage**

- **Storage**: Documents created according to the SOP are transferred to and stored by the responsible investigator.

This SOP outlines detailed procedures and requirements to ensure proper evaluation and reliability of the trial, ensuring the integrity and trustworthiness of the study.
